# Supplementary material for: RNA structure prediction using positive and negative evolutionary information
Source: PLoS Comput Biol. 2020 Oct 30;16(10):e1008387. doi: 10.1371/journal.pcbi.1008387 (PMC7657543; doi:10.1371/journal.pcbi.1008387)
Supplement: S1 File — Includes alignments, and source code. (GZ) [file pcbi.1008387.s008.tar.gz › supplemental_material/src/rscape_v1.5.4.tar.gz!/rscape_v1.5.4/lib/R2R/R2R-current/R2R-manual.pdf]

R2R version 1.0.6.1-49-g7bb81fb user manual

Software to speed the depiction of aesthetic consensus RNA secondary structures

Zasha Weinberg

November 6, 2019

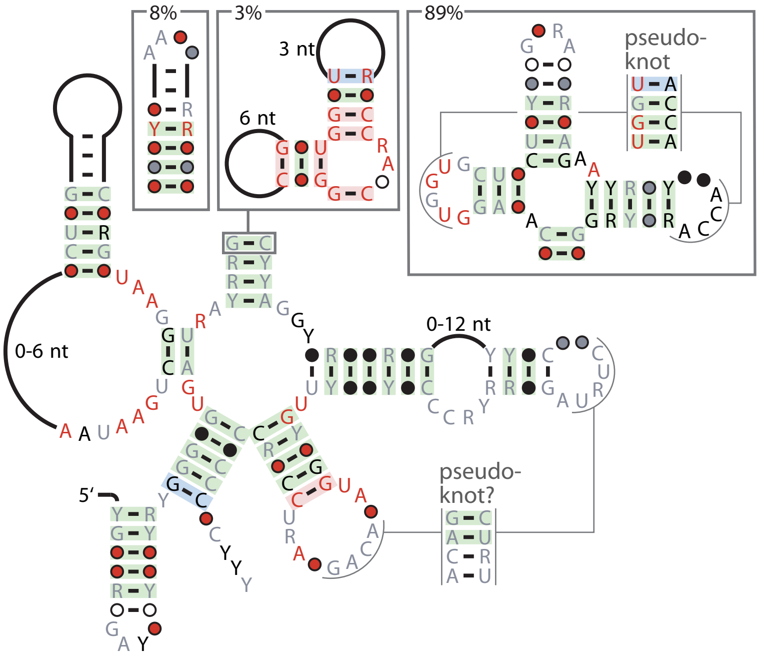

Copyright © 2009-2019 by Zasha Weinberg. All rights reserved.

This software package, including source code and this manual, is distributed freely but without any warranty under the terms of the GNU General Public License as published by the Free Software Foundation. This program is distributed in the hope that it will be useful, but WITHOUT ANY WARRANTY; without even the implied warranty of MERCHANTABILITY or FITNESS FOR A PARTICULAR PURPOSE. For a copy of the full text of the GNU General Public License, see www.gnu.org/licenses.

This package includes the following material from other sources:

- The "Squid" library from Infernal version 0.7 by Sean Eddy.
- Free code that is part of the CFSQP package [5], distributed by AEM Design.
- Previously published layouts of RNAs and R2R markup to generate those layouts [13, 17, 15, 16, 12, 9, 11, 6].

1 Introduction **5**
1.1 What does this software do? . . . 5
1.2 What does this software *not* do? . . . 5
1.3 Credit . . . 6
1.4 Licensing . . . 6
1.5 Changes from previous versions . . . 6

2 Installation **10**
2.1 Platforms on which R2R is known to work . . . 10
2.2 Decide what NLP solver to use (if any) . . . 10
2.3 Build R2R . . . 11
2.3.1 Extract files from the R2R .tgz archive file . . . 11
2.3.2 Optionally choose an SVG font (very unusual) . . . 11
2.3.3 Optionally install NLOPT . . . 12
2.3.4 Optionally get CFSQP . . . 12
2.3.5 configure and make . . . 12
2.4 Adobe Reader glitch . . . 13
2.5 Alignment editor software . . . 13

3 Tutorial: a guide to R2R with examples **14**
3.1 About the demo files . . . 14
3.1.1 Demo output is already provided . . . 14
3.1.2 Drawing programs: Adobe Illustrator or Inkscape . . . 14
3.1.2.1 More technical information . . . 15
3.1.3 Where the demo files come from . . . 15
3.2 Example legend to help you with finished drawings . . . 16
3.3 A hypothetical motif to illustrate the basics . . . 16
3.3.1 Default output of R2R . . . 16
3.3.2 Some typical customizations for consensus diagrams . . . 18
3.3.3 Other kinds of drawings: single-molecule and skeleton diagrams . . . 19
3.4 Common error: “One or more pairs is getting broken by one side getting deleted” . . . 22
3.5 The place_explicit command . . . 22
3.6 Multistem junctions and turning internal loops . . . 24
3.6.1 Simple circular layout . . . 24
3.6.2 Manual layout of multistem junctions . . . 27
3.6.3 Automated multistem junction layout with direction constraints . . . 28
3.6.3.1 Solving multistem junctions can be slow . . . 32
3.6.4 Internal loops . . . 32
3.7 Modular structures . . . 32
3.8 Pseudoknots . . . 36

3.8.1 A note on the representation of pseudoknotted secondary structures within Stockholm files . . . 36
3.8.2 Pseudoknots drawn with callouts . . . 36
3.8.3 Pseudoknots drawn with in-line style . . . 38
3.9 Preparing presentations using projectors . . . 38
3.10 How to draw GOLLD RNA from R2R output . . . 40
3.11 Making the demo output files . . . 40

4 Reference: automated inference of nucleotide conservation levels **41**
4.1 Recommended command . . . 41
4.2 "Fragmentary" alignments . . . 42
4.3 Weighting sequences using your own algorithms (for calculating nucleotide frequencies) 42
4.3.1 Why R2R weights sequences . . . 42
4.3.2 How to use your own weights . . . 43
4.4 General command . . . 43
4.5 Output of R2R for the consensus . . . 45
4.6 Generating your own alignment consensus, bypassing R2R . . . 45

5 Reference: drawing **47**
5.1 Running R2R . . . 47
5.2 About the Stockholm file format, and how R2R uses it . . . 48
5.2.1 A note on R2R's representation of consensus secondary structures . . . 48
5.2.2 R2R does not conform to the Stockholm file format . . . 49
5.3 R2R "drawing units" . . . 49
5.4 Data types in R2R . . . 50
5.4.1 hitId . . . 50
5.4.2 Distances . . . 50
5.4.3 Angles . . . 50
5.4.4 Measurements (length/width/size) . . . 50
5.4.5 Colors . . . 50
5.5 .r2r_meta file . . . 51
5.5.1 Display name . . . 51
5.5.2 Defines . . . 51
5.5.3 SetDrawingParam . . . 51
5.5.4 Oneseq mode . . . 57
5.5.5 Skeleton mode . . . 57
5.5.6 Entropy mode . . . 57
5.5.7 Cleavage diagrams . . . 57
5.5.7.1 Multiple drawings of the same RNA molecule . . . 58
5.6 The R2R solver cache . . . 58
5.7 Labels . . . 59
5.7.1 Main labels . . . 59
5.7.2 Extra named label lines . . . 59
5.7.3 Sequence-specific (optional) . . . 60
5.7.4 SS_cons . . . 60
5.7.5 Using labels and special labels . . . 60
5.8 R2R commands . . . 61
5.8.1 Conditional commands . . . 61

5.8.1.1 Define symbols . . . 61
5.8.1.2 Commands that apply to only a consensus or single-molecule drawing 62
5.8.1.3 Other kinds of conditional commands . . . 62
5.8.2 Turning and positioning . . . 63
5.8.2.1 Set_dir . . . 63
5.8.2.2 Laying out arbitrary units like a bulge . . . 63
5.8.2.3 Layout single-stranded loops along a straight line, instead of circle . 63
5.8.2.4 turn_ss . . . 63
5.8.2.5 turn_stem_at_internal . . . 64
5.8.2.6 disconnect_from_5prime . . . 64
5.8.2.7 split_ss . . . 64
5.8.2.8 place_explicit . . . 64
5.8.3 Layout of multi-stem junctions . . . 65
5.8.3.1 Manual layout . . . 65
5.8.3.2 Multi-stem junctions: automatic circular layout . . . 67
5.8.4 Changing layout of secondary structure . . . 72
5.8.4.1 depair . . . 72
5.8.4.2 make_pair . . . 72
5.8.4.3 Internal_loop_to_bulges . . . 72
5.8.4.4 Ignore pseudoknots entirely . . . 72
5.8.4.5 Ignoring pseudoknots for the purposes of layout . . . 73
5.8.4.6 subst_ss . . . 73
5.8.4.7 merge_ss . . . 73
5.8.5 variable-length regions . . . 74
5.8.5.1 var_hairpin . . . 74
5.8.5.2 var_term_loop . . . 74
5.8.5.3 Variable-length backbone . . . 74
5.8.5.4 var_stem . . . 75
5.8.6 Annotation . . . 75
5.8.6.1 Tick labels (like the nucleotide numbering in cleavage diagrams) . . 75
5.8.6.2 nobpannot . . . 76
5.8.6.3 Pseudo-bold fonts . . . 76
5.8.6.4 Changing nucleotide colors . . . 76
5.8.6.5 Circling nucleotides . . . 76
5.8.6.6 Boxing nucleotides . . . 77
5.8.6.7 Outlining/inlining nucleotides . . . 77
5.8.6.8 Boxing a set of nucleotides . . . 77
5.8.6.9 Shading nucleotides along the backbone . . . 77
5.8.6.10 Outlining a stretch of nucleotides around both ends . . . 78
5.8.6.11 Shading the backbone in skeleton drawings . . . 78
5.8.6.12 Drawing circles associated with loops . . . 78
5.8.6.13 Drawing direct lines between consecutive nucleotides. . . . 79
5.8.7 Miscellaneous . . . 79
5.8.7.1 Override default parameters for drawing . . . 79
5.8.7.2 No 5 prime label . . . 79
5.8.7.3 Adding G for transcription . . . 79
5.8.7.4 Keeping gap columns in the drawing . . . 79
5.8.7.5 Breaking pairs . . . 79

5.8.7.6 Explicitly setting/overriding the covariation shading 80
5.8.7.7 Deleting columns using an explicit command 80
5.9 Troubleshooting 80
5.10 Text output of r2r useful in debugging 81
5.10.1 Start/end of file 81
5.10.2 consensus lines and “raw” coordinates 81
5.10.3 Preliminary secondary structure drawing units 81
5.10.4 place_explicit links 82
5.10.5 Selecting the place explicit commands 83
5.11 Interacting with R2R using scripts 83
5.11.1 Generating R2R input using scripts 83
5.11.2 Accessing layout information from R2R using scripts 84

6 Reference: modular structures **86**
6.1 Sub-families 86
6.2 Using `SelectSubFamilyFromStockholm.pl` 86
6.2.1 Command line 86
6.2.2 How to define predicates 87
6.2.2.1 Regex predicates 87
6.2.2.2 Perl predicates 87
6.2.3 How the file is modified by applying a predicate 89

7 Meta-Makefile **90**
7.1 Running the script 90
7.1.1 Script options 90
7.1.2 Makefile targets 92
7.2 Additional markup for .sto files 92
7.2.1 Implicit rules 92
7.2.2 Explicit rules 92
7.2.2.1 Oneseq 93
7.2.2.2 Skeleton 93
7.2.2.3 Defines for consensus diagrams 93
7.2.2.4 Using define directive with modular structure 93
7.2.2.5 Oneseq for a predicate defining a modular structure 93
7.2.2.6 Using a different sequence-weighting algorithm 93
7.2.2.7 Weighting sequences using your own algorithms with MetamakeDemos 94

8 R2R source code **95**
8.1 Summary of C++ and Perl source code files 95
8.2 Other information 97
8.2.1 Overall layout of RNA 97
8.2.2 Inferring a path for the backbone 97

Chapter 1

Introduction

1.1 What does this software do?

For a full description of this software, please read the paper titled "R2R—software to speed the depiction of aesthetic consensus RNA secondary structures" [14].

Briefly, this software is designed to speed the drawing of RNA secondary structure consensus diagrams, which show the conserved features within a set of related RNAs. The software also supports drawing of single RNA molecules, although this is not the emphasis. To make RNA drawings, many biologists use general-purpose software such as Adobe Illustrator, at great cost in time and risk of errors. However, this strategy produces the highest-quality of drawings. R2R is designed to allow the user to achieve this highest-quality drawing, while taking much less time than the manual solution. Because of this goal, R2R imposes more work on the user than highly automated solutions, and the current version of R2R is aimed at bioinformaticians, or biologists with some familiarity with UNIX-like command line tools.

I have used R2R to draw over 100 RNA consensus diagrams. These drawings are made available as "demo" files (see Chapter 3).

1.2 What does this software *not* do?

R2R has some important limitations:

- R2R does not fully automate determination of a layout. Although its default layouts will get you closer to an ideal layout and it has functions to assist in determining layouts of multistem junctions, R2R does not solve the problem of determining an overall layout. No currently available computer algorithm can determine an ideal layout that is comparable in quality to the best layouts found by a person. Therefore, R2R assumes that a user will optimize the layout and tell R2R what to do.

- R2R does not have a graphical user interface. As noted above, R2R is aimed at bioinformaticians. Successful use of R2R will likely require some general familiarity with the UNIX command line, and comfort with command-driven programs. Because of the lack of a graphical user interface, R2R might have a significant learning curve. So, if you just want to draw one RNA, it might be most efficient to go straight to Adobe Illustrator, Inkscape or CorelDRAW.

- R2R is not designed to produce drawings that illustrate many elements of tertiary structure, or whose layouts are based on atomic-resolution 3-D structures. R2R is only intended to create more abstract layouts of secondary structure that reflect Watson-Crick base pairing.

- R2R is not a fully general drawing program, or even a fully general RNA-drawing program. R2R should be used in combination with general-purpose drawing programs like Adobe Illustrator or Inkscape.
- R2R does not quite respect the Stockholm file format. See section 5.2.2 for details.

1.3 Credit

If you use R2R, please cite the paper [14].

If you distribute R2R or derivatives of it, please continue to credit Infernal, as on the first page of this manual.

1.4 Licensing

The files in the main directory (documentation), the `src` and the `demo` subdirectories are copyright 2009-2019 by Zasha Weinberg, except as noted. The entire package is distributed freely but without any warranty whatsoever under the GNU General Public License. For details, see `http://www.gnu.org/licenses`.

1.5 Changes from previous versions

- Version 1.0.7 (anticipated)
  - Added the ability to change the names of the individual drawings that R2R outputs into PDF of SVG files. This is for use by R-scape. See Section 5.5.1.
  - Added ability to change the name of the font that R2R puts in SVG files, so that Inkscape is happier. This has no effect on generated PDF files. See Section 5.1 and Section 7.1.1.
  - When ignoring secondary structures line (usually for pseudoknots), you can now get R2R to draw an outline around the paired nucleotides, without drawing colored rectangles (for covariation) that relate to that secondary structure line. See the `outline-no-bpannot` option in section 5.8.4.5.
  - Added ability to use different sequence-weighting algorithm with the Makefile functionality. See Section 7.2.2.6.
  - With the the Makefile functionality, added ability to set a global set of drawing parameter values (See Section 5.5.3 for information on drawing parameters) that apply to all drawings, effectively setting new default values for parameters, in case you don't like the default values I selected. (See Section 7.1.1.)
  - R2R will now attempt to draw the text marking the 5′ end, as well as a line connecting to the first nucleotide, even if the first nucleotide runs at a weird angle (i.e., not a multiple of 90). It seems to mostly look reasonable. This is influenced by the new `backboneConnectorCircleRadius` drawing param (See Section 5.5.3.), but the default value seems to work pretty reasonably.
  - Added an ability to control what R2R calls the various pseudoknots in callout-style drawings of pseudoknots. See the `prefixSsWithPkInDrawings` parameter in Section 5.5.3. This feature is for R-scape.

- Added various drawing parameters (See Section 5.5.3.):
  * `nucTickLabel_tickColor`, which determines the color of all lines that are part of tick labels. There is no way to set the color of individual tick lines.
  * `varBackboneFontSize` : font size for text with `var_backbone` and related commands.
  * `varTermLoopFontSize` : font size for text with `var_term_loop` command.
  * `backboneAnnotTextOffset` : is now, by default, set based on `varBackboneFontSize`. (And it's now documented.)
  * `backboneConnectorCircleRadius` :the radius of the arc that R2R uses to bend the backbone, esp. when drawing the 5′ end of the molecule.
- The script `SelectSubFamilyFromStockholm.pl` is now installed into the `bin` directory in `make install`.
- added `--cutEmptyLines` flag for `r2r`, to supress blank lines added in some circumstances by the `--GSC-weighted-consensus` flag. These blank lines are intended to preserve line numbers, so that error messages make sense with the original file, but they violate Stockholm format, which is a hassle in some cases. (See Section 4.4.)
- Fixed a bug where an input file without a `#=GC SS_cons` line caused a segmentation fault.
- Documented incompatibilities between R2R and the Stockholm format. See section 5.2.2.
- Documented other flags for `r2r` with the `--GSC-weighted-consensus` flag. See Section 4.4.
- Added ability to explicitly set the covariation shading of base pairs. See Section 5.8.7.6.

- Version 1.0.6
  - Integrated NLOPT optimization library as an alternative to CFSQP. NLOPT does not seem to work quite as well as CFSQP for some problems posed by R2R. But, it mostly works comparably and is free software, whereas CFSQP is annoying to obtain. Note: NLOPT has a lot of solvers and parameters I didn't try out, so it's possible that it could work better if set up differently.
  - Added ability to have secondary structure lines for drawing individual sequences (i.e., in `oneseq` mode). Any line beginning `#=GR` *hitId* `SS_cons_`*name* will be addressable as a secondary structure under the name "_*name*".
  - R2R now has an option to change the meaning of red shading of base pairs. Red shading means there is no variation in the base pair. In this case, covariation could arise simply due to an absence of variation, and there is no evidence against base pairing. However, due to a detail in implementation, R2R previously allowed for rare occurrences (by default up to 10%) of non-canonical base pairs. This remains the default behavior, but there is an option to avoid the red shading in cases where there are non-canonical pairs.
  - Other minor changes / bug fixes.

- Version 1.0.5
  - R2R now has a `./configure` script. The installation instructions have changed accordingly.

- The manual has been corrected as follows. R2R does not work with the interleaved format of Stockholm format alignments. R2R only works with the Pfam format, which is equivalent to Stockholm format, except that the full sequence or column annotation must be given in one line (for hit sequences, or data in #=GC or #=GR lines), and these lines cannot be split.
- R2R now prints a warning about inappropriate use of R2R, specifically against using R2R to evaluate structural conservation. R2R is not intended to evaluate evidence for covariation or RNA structure where this is in question. It is not appropriate to use R2R's covariation markings to declare that there is evidence of structural conservation within an alignment. R2R is a drawing program. As the R2R paper wrote[14]:

  > This automated R2R annotation [of covariation] does not reflect the extent or confidence of covariation. While such information can be useful, we believe that thorough evaluation of covariation evidence ultimately requires analysis of the full sequence alignment. For example, misleading covariation can result from an incorrect alignment of sequences, or from alignments of sequences that do not function as structured RNAs. Unfortunately, there is no accepted method to assign confidence that entirely eliminates the need to analyze the full alignment.

  You can disable this warning by running R2R with the flag `--disable-usage-warning`.
- When drawing a single sequence (i.e., in `oneseq` mode), R2R can highlight nucleotides that are highly conserved in the consensus. This feature makes these highly conserved nucleotides apparent when, for example, depicting an RNA molecule on which experiments were done, and where mutations might have been made. To enable this feature, see `makeRedNucsRedInOneseq` and `makeNondegenRedNucsRedInOneseq` in Section 5.5.3.
- Added the command `tick_label_disable_default_numbering` and the ability to start numbering nucleotides with an arbitrary number, by adding `firstNucNum` to the `tick_label_regular_numbering` command. See Section 5.8.6.1
- Improved miscellaneous error messages.

- Version 1.0.4
  - Fixed problem where tab characters between sequence name and nucleotides within the input alignment can cause errors. Tab characters are now treated like spaces.
  - When you use the `ignore_ss_except_for_pairs` command with the `outline` option, R2R will annotate the pseudoknot outlines with the name of the corresponding `#=GC SS_cons` line. This helps to match up pseudoknots for RNAs that contain multiple pseudoknots.
  - Changes to `SelectSubFamilyFromStockholm.pl`, allowing a predicate to easily use markup from a name other than the predicate's name. See `SUBFAM_STRING` field in `SUBFAM_REGEX_PRED` and `SUBFAM_PERL_PRED` (Section 6.2.2).
  - Fixed typo in tutorial.
- Version 1.0.3 (August, 2012)
  - R2R 1.0.3 cannot exactly reproduce older drawings of RNA structures. If you want to reproduce the old drawings, you should use R2R version 1.0.1.

The way that R2R handles degenerate nucleotides when calculating the consensus sequence has changed. Degenerate nucleotides are now ignored in the calculations, where previously they were treated as a uniform distribution of the possible nucleotides (e.g., degenerate nucleotide 'R' was treated as 50% A and 50% G). As a result, the current R2R does not exactly reproduce old drawings. In some cases, the layouts change when the differences lead to nucleotides falling above or below the 50%-present level, thus adding or removing nucleotide positions from the drawing.

  - Added ability to use alignment positions as labels. See Section 5.7.5
  - Added `dumpInfoFile` drawing parameter, which dumps information on layout to a tab-delimited file.
  - Added section on interacting with R2R using scripts. See Section 5.11.

- Version 1.0.2
  - Added citation of R2R paper in *BMC Bioinformatics* to this manual.
  - Edited some error messages to make them more clear.
  - Check for conflicting use of `bulge` and `place_explicit`.
  - Added `make_pair` command.
  - Added `DNA`, `shadeAlongBackboneWidth` and `alongBackboneMidpointGapLength` drawing parameters.
  - Added ability for user to weight sequences using an arbitrary method (instead of the GSC algorithm), while calculating frequencies otherwise as before. See Section 4.3 and Section 7.2.2.7.

- Version 1.0.1 (distributed with revised version of paper, November 2010)
  - Added demo: `demo/c-di-GMP-II/c-di-GMP-II-update.sto` (re-drawing based on new sequences, for Wikipedia article), also some files in the `demo/pedagogical` directory.
  - Added Additional files 3 and 4 from the paper to software distribution.
  - Added feature: `indicateOneseqWobblesAndNonCanonicals` added as a drawing parameter.
  - Fixed bugs:
    * Fixed a problem when internal loops are converted to bulges (explicitly using the `internal_loop_to_bulges` command, or implicitly by applying a `bulge` or `place_explicit` command to a nucleotide within the internal loop), and when the user did not specify what to do with both sides of the internal loop.
    * Fixed various issues with applying the `multistem_junction_circular` command to 3′ bulges.
    * Formatting issues with the user manual. The `--GSC-weighted-consensus` flag was explained in more detail.

- Version 1.0 (distributed with initial submission of paper, July 2010)

Chapter 2

Installation

Installation essentially means making the executable file `r2r`.

2.1 Platforms on which R2R is known to work

Building of R2R has only been tested with the gcc compiler suite (http://gcc.gnu.org). To ensure compatibility, you will need the GNU C compiler (which provides the `gcc` command) and the GNU C++ compiler (which provides the `g++` command).

R2R has been tested on the following platforms, however only the original version:

- gcc version 3.4.4 under Cygwin 1.5.25 (32-bit) on Windows XP. NOTE: you'll need to install the C/C++ compiler gcc and the make program. These are not installed by default in Cygwin.
- gcc version 4.3.4 under Cygwin 1.7.1 (32-bit) on Windows 7.
- gcc version 5.3.0 under Cygwin 64-bit on Windows 7. R2R version 1.0.5 works on this platform.
- gcc version 4.1.2 under Ret Hat Linux running Linux kernel 2.6.18 (64-bit). R2R version 1.0.5 works on this platform.
- gcc version 3.3 under MacOS's Darwin version 8.8.0 (32-bit). NOTE: you'll need to install the Xcode package to get the C/C++ compiler gcc and the make program.

I presume that R2R will work on gcc version 3 or higher on Cygwin, Linux or MacOS Darwin.

R2R is known to produce output that can be used with Adobe Illustrator CS on Windows XP, Inkscape 0.46 on Windows XP and CorelDRAW Graphics Suite X4 on Windows Vista. I presume, however, that the output will work on any version of these programs, at least with appropriate setting of fonts (see below).

2.2 Decide what NLP solver to use (if any)

R2R implements multiple methods to find a good layout for multistem junctions. Some automated solutions require software that can solve non-linear optimization problems. You have three options: don't install any NLP solver, install the NLOPT solver or install the CFSQP solver.

These options affect whether you can use the following commands that using a non-linear optimization to solve the layout of multistem junctions: `multistem_junction_circular_solver`, `multistem_junction_bulgecircley_solver` and `multistem_junction_bulgecircleynormals_solver`. If you do not use these commands, there's no advantage to installing an NLP solver.

- Option 1: Do not install a solver. (This is the default.)
  - Effort required of you: none. It's the default.
  - Ability to solve multistem junctions: none. The functions requiring NLP optimization will not work if there's no solver. If you try to use one of the related commands, R2R will report an error.
- Option 2: Install NLOPT.
  - Effort required of you: a bit. You must download & install the NLOPT library, but that's not especially hard.
  - Ability to solve multistem junctions: pretty good. In some cases, NLOPT does not work as well as CFSQP (see next option, below). NLOPT is more often than CFSQP unable to solve the optimization problem well. However, most of the time, NLOPT and CFSQP yield the same result. Note: NLOPT has a lot of solvers and parameters I didn't try out, so it's possible that it could work better if set up differently.
- Option 3: Install CFSQP.
  - Effort required of you: more. Due to its licensing requirements, CFSQP must be requested from AEM Design, which requires additional time, and has been a hassle in the past. (I am not legally allowed to distribute CFSQP with R2R.) Once you get the relevant file, however, it's pretty straightforward.
  - Ability to solve multistem junctions: good. CFSQP is not always able to do it, but it works well most of the time.

2.3 Build R2R

2.3.1 Extract files from the R2R .tgz archive file

The R2R files are contained within the .tgz file. You can extract these files using WinZip on Windows or by double-clicking on Mac OS X. In a UNIX system (including Darwin and Cygwin), you can run a command like `tar xzf /path/to/where/you/downloaded/R2R.tgz`.

2.3.2 Optionally choose an SVG font (very unusual)

If you intend to use Adobe Illustrator or CorelDRAW, you can use the PDF (Adobe Acrobat) output of R2R. In this case, you do not need to set a font.

If you intend to use Inkscape, you should use the SVG output of R2R, since Inkscape is not reliable when importing PDFs generated by R2R. By default, R2R is set up to produce SVG output using the "Bitstream Vera Sans" font, which is freely available (http://www.gnome.org/fonts/) and might already be installed on your system. If, however, Inkscape is not mapping your font correctly, you can get R2R to generate SVG files that reference a different font.

(Note: it might be easiest to skip this step initially, and change the font later if there are problems in Inkscape. To change the font later, you'll need to edit `src/Makefile.in`, and run `./configure` again, as described below.)

To change this font, open the file `src/Makefile.in` in a text editor and change the definition of the `INKSCAPE_HELVETICA_FONTNAME` define within the double quotes. (Search for "FONTNAME".) I'm not sure that anyone really has to do this, so I haven't made this part of the configure script.

Note: R2R is internally hardcoded with font geometry that is appropriate to Helvetica, Arial, Bitstream Vera or DejaVu fonts. These fonts have similar sizes to each other. If you use fonts whose symbols have significantly different sizes, nucleotides will not be positioned correctly.

2.3.3 Optionally install NLOPT

If you want to use NLOPT, you'll have to install the library. The home page for the NLOPT library is here: https://nlopt.readthedocs.io/en/latest/

When you build R2R, pass the `--enable-nlopt` flag to `./configure` (see below).

2.3.4 Optionally get CFSQP

If you want to use the CFSQP solver, you must request the proprietary file `cfsqp.c`. Go to http://www.umventures.org/technologies/cfsqp%E2%84%A2-version-23 or https://www.isr.umd.edu/news/news_story.php?id=4087 or Google for CFSQP. (The contact has changed recently.)

Once you have obtained it, put the file `cfsqp.c` into the R2R directory `NotByZasha/cfsqp`.

When you build R2R, pass the `--enable-cfsqp` flag to `./configure` (see below).

2.3.5 configure and make

Run `./configure` to configure R2R for your system.

```
./configure
```

The `./configure` script can take the following flags, in addition to the standard `./configure` script flags (run `./configure --help` for a complete list):

- `--enable-nlopt` : compile with NLOPT. Note: this requires that the compiler can find the NLOPT include and library files. You might need to adjust the CFLAGS and LDFLAGS environment variables appropriately before running `./configure` so that it can find the NLOPT files.

- `--enable-cfsqp` : compile with CFSQP. Note: this requires that you have the file `cfsqp.c` (see previous section) If you enable both NLOPT and CFSQP, then R2R will use CFSQP.

Then run

```
make
```

Note: if you include CFSQP, you might get warnings about redefinitions of the symbol `__STDC__`. These warnings are benign, and you can ignore them.

If you want to copy the executable into your binary directory, optionally run

```
make install
```

Otherwise, you can use the `r2r` executable file from within the R2R package directory.

2.4 Adobe Reader glitch

This section describes a potential problem when viewing PDF output with Adobe Reader. With recent versions of Adobe Reader and certain computer configurations, circles and arcs appear jagged—more like polygons. To prevent this, while running Adobe Reader, select “Preferences” under the “Edit” menu. Within the category “Page Display”, uncheck “Use 2D graphics acceleration” and make sure that “Smooth line art” is checked.

2.5 Alignment editor software

R2R’s input is based upon multiple-sequence alignments that are stored in Pfam format, which is a special case of Stockholm format in which interleaved lines are not permitted. Although these files can be edited in any text editor, some programs are customized for them. One example is RALEE [4], which is a set of macros for the Emacs text editor. Please see section 5.2.2 for a full discussion of how R2R does not fully implement the Stockholm file format. You can convert Stockholm-format alignments into the Pfam format using the `esl-reformat` command that comes with the HMMER and Infernal software packages in the `easel/miniapps` directory.

Chapter 3

Tutorial: a guide to R2R with examples

This chapter gives a practical introduction with examples on how to create RNA drawings with R2R. Example input files containing R2R commands to draw various RNA structures are available in the `demo` subdirectory.

Credit: the Stockholm-format input files in the `demo` directory and its subdirectories are similar or identical to supplementary data published in previous reports [13, 16, 9, 12, 17, 15, 6].

3.1 About the demo files

3.1.1 Demo output is already provided

I have created the raw output of R2R when run on the demo files, and this output is provided within the `output-pdf` (PDF format) and `output-svg` (SVG format) directories. If you just want to see the output, I recommend opening the PDF files using Adobe Reader (`http://get.adobe.com/reader`), as this should work on any platform. (Note: if circles look very unsmooth when viewed in Adobe Reader, please see Section 2.4.) If you want to try editing the output files, please see Section 3.1.2 (next) to find out which files to use.

If you want to create the output files yourself, see Section 3.11.

3.1.2 Drawing programs: Adobe Illustrator or Inkscape

Simple conclusion:

- If you're using Adobe Illustrator or CorelDRAW, use PDF output. The example commands below use PDF.

- If you're using Inkscape, use SVG output. In the commands below, wherever it says `pdf`, instead write `svg`. (R2R decides the output format based on the file extension of its output file, either `.pdf` or `.svg`.)

  If you have problem with the fonts in Inkscape (or if the letters don't show up), you might need to re-create the SVG output with a different font name. Please see the next section ("more technical information") to learn more about this, and Chapter 2 for information on how to change the font.

3.1.2.1 More technical information

R2R will generate valid PDF and SVG files as output. In principle, any program should be able to read them. However, I am exploiting the fact that the Helvetica font is built in to PDF. Some versions of Inkscape will not render any text using PDFs built by R2R because they don't have the Helvetica font.

Therefore, R2R can create SVG output, which is Inkscape's native file format. The SVG output uses the Bitstream Vera Sans font, which is similar to Helvetica and to Inkscape's default font. (Meanwhile, at least on Windows, Illustrator will substitute Arial, which is similar.)

Note: in principle you can substitute other fonts, even by creating scripts to edit SVG output. However, R2R knows the heights and width of font symbols, which is uses for its layout. Thus, for example, if you use a narrower font, the spacing might not be appropriate.

3.1.3 Where the demo files come from

Files within the `demo` directory were made to support this user manual. Most are based on previously drawn RNAs. RNA drawings derived from previous studies are organized into subdirectories. The subdirectories and relevant citations are as follows:

- `demo/22` (Weinberg *et al.*, 2007) [13] and (Sudarsan *et al.*, 2008) [11].
- `demo/104` (Weinberg *et al.*, 2010) [17].
- `demo/exceptional` (Weinberg *et al.*, 2009) [15]. Note:
  - The RNAs depicted in Supplementary Figure 11 of this paper are primarily described in the (Weinberg *et al.*, 2010) paper [17], and are therefore found within the `demo/104` directory.
  - The drawing of GOLLD RNA is somewhat complicated by different degrees of conservation of the structural elements. For instructions on reproducing the drawing of GOLLD RNA in Fig. 2a, see Section 3.10.

    Also, a careful look at Fig. 2a in *Nature* will reveal that many nucleotides are slightly misaligned. The reason is that the R2R drawings in this case were done using Adobe's Myriad font, which is narrower than Helvetica. The journal changed the font, but did not realign the nucleotide letters. The output of R2R is always aligned correctly.
  - The HEARO RNA "skeleton" drawing in Figure 3b was not drawn with R2R. However, an R2R drawing of this style is included. Figure 3b was drawn before I had implemented skeleton drawings in R2R.
- `demo/c-di-GMP-II` (Lee *et al.*, 2010) [6]
- `demo/Moco` (Regulski *et al.*, 2008) [9]. (New drawing, based on original layout.)
- `demo/SAH` (Wang *et al.*, 2008) [12]. (New drawing, based on original layout.)
- `demo/SAM-IV` (Weinberg *et al.*, 2008) [16].
- `demo/hammerhead` (Perreault *et al.*, 2010) [7]
- `demo/additional` Drawings of RNAs that were identified by other groups. The tRNA drawing is based on the standard layout.

- `demo/pedagogical` Some drawings I did to explain issues in RNA drawing to non-biologists, or for the R2R paper.

Figures within each directory are organized by motif name. Thus, for example, in (Weinberg *et al.*, 2009) [15], Figure 2a is a consensus diagram of GOLLD RNA. This corresponds to the files `demo/exceptional/GOLLD.sto` and `demo/exceptional/GOLLD.r2r_meta`. If you run `r2r` on these files, the output will be found in `output-pdf/GOLLD.pdf` or `output-svg/GOLLD.svg`. Similarly, Supplementary Figure 1 of the same previous publication contains a skeleton diagram of GOLLD RNA, Supplementary Figure 2 has an expanded consensus diagram and Supplementary Figure 3 holds a different skeleton diagram and a drawing of certain parts of the *L. brevis* GOLLD RNA. All these figures are generated from the same files, and R2R output used for this will also appear in `output-pdf/GOLLD.pdf` or `output-svg/GOLLD.svg`. However, updates to the way that consensus nucleotides are calculated with degenerate IUPAC nucleotides have slightly changed the picture. Versions of R2R up to 1.0.1 will exactly reproduce the old drawing.

3.2 Example legend to help you with finished drawings

To create a finished drawing for a published figure, a legend is necessary to explain the annotations. We have created generic annotations usable for a legend:

- Use the file `demo/Additional-file-3.pdf` for PDF format that can be imported into Adobe Illustrator or CorelDRAW.
- Use the file `demo/Additional-file-4.svg` for SVG format that can be imported into Inkscape.

3.3 A hypothetical motif to illustrate the basics

Now the actual tutorial begins.

3.3.1 Default output of R2R

Figure 3.1 shows a simple alignment, and R2R's drawing of the consensus of the alignment. Elements of the Stockholm-format alignment file are illustrated. Note that R2R cannot process "interleaved" Stockholm files, in which sequences can span multiple lines; this restricted form of the Stockholm format is called the Pfam format. Please see section 5.2.2 for a complete discussion on how R2R does not fully conform to the Stockholm file format. You can convert Stockholm-format alignments into the Pfam format using the `esl-reformat` command that comes with the HMMER and Infernal software packages in the `easel/miniapps` directory.

The drawing was made by running the following commands within the `demo` directory. (Actually, I really just ran `make`, which reads the `Makefile` in that directory to run these commands. If you're familiar with the UNIX `make` command, and you're going to make many drawings, you might want to read Chapter 7 after reading this tutorial.)

First, in order to draw the consensus structure, we must first run a command to calculate it. We calculate the alignment's consensus (Figure 3.1D) using the following command line (note: the following command should be one line, but was split into two lines for typesetting):

```
commandprompt$ ../src/r2r --GSC-weighted-consensus demo1.sto    (continued)
    intermediate/demo1.cons.sto 3 0.97 0.9 0.75 4 0.97 0.9 0.75 0.5 0.1
```

Figure 3.1 demo1: A simple alignment and R2R drawing

A

```
# STOCKHOLM 1.0
human         ACACGCGAAA.GCGCAA.CAAACGUGCACGG
chimp         GAAUGUGAAAAACACCA.CUCUUGAGGACCU
bigfoot       UUGAG.UUCG..CUCGUUUUCUCGAGUACAC
#=GC SS_cons  ...<<<.....>>>....<<.....>>.....
//
```

B

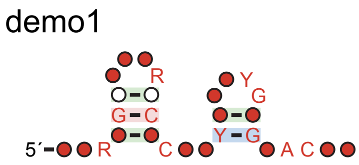

C

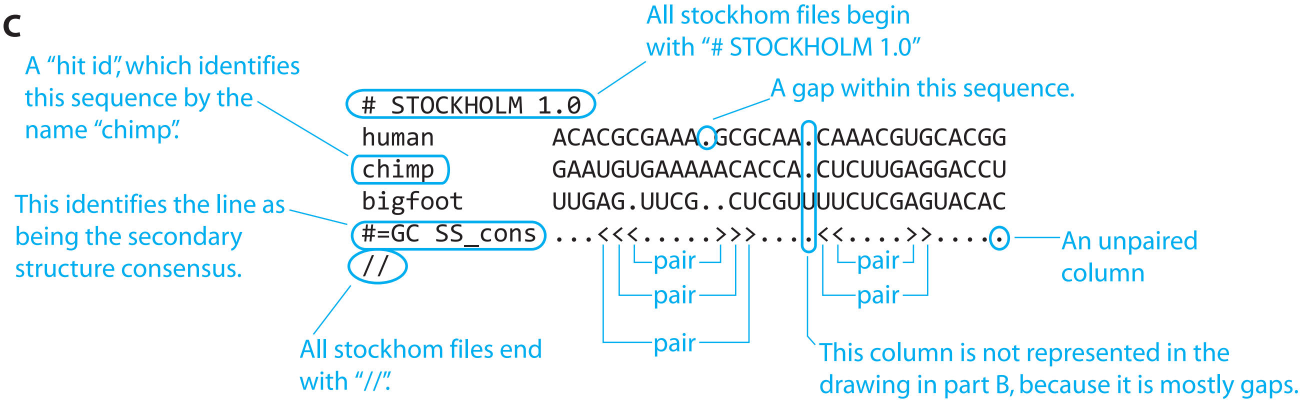

D

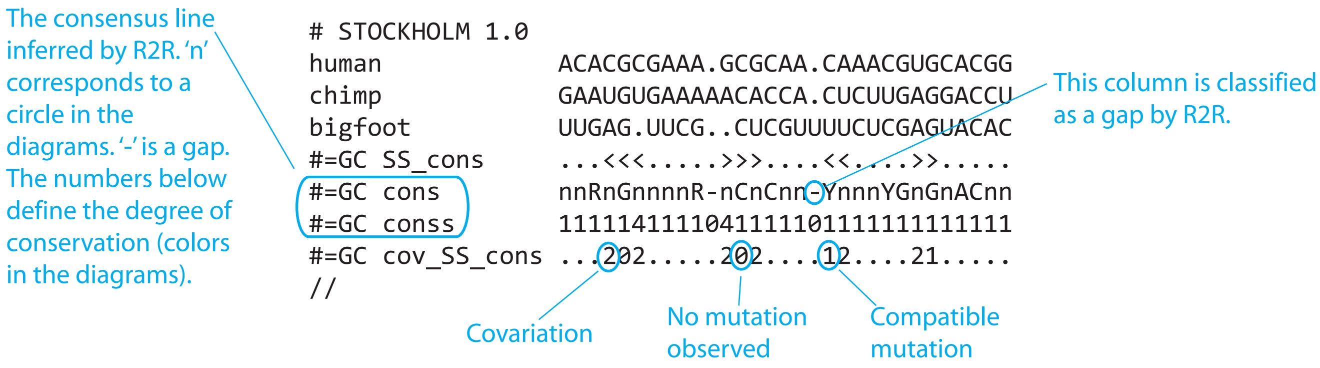

E `intermediate/demo1.cons.sto`

(A) A minimal Stockholm-format alignment of a hypothetical RNA motif. This is the file `demo/demo1.sto`. (B) The raw output of R2R when run on the alignment in part A. Note that the text "demo1" is part of R2R's raw output. (C) Annotations of the alignment file. (D) The Stockholm-format alignment file generated by R2R (with the `--GSC-weighted-consensus` flag) that has information on the consensus of the demo1 motif. (Some minor changes were made to improve readability.) Note that it is not necessary to understand the information in this file, because the file is simply used by R2R to produce its final output. The way in which R2R calculates consensus diagrams, and the meanings of the colors and symbols is explained in the research paper, as well as in Chapter 4. (E) The contents of the file `demo/demo.r2r_meta`. In this case, the file simply lists the name of input file used to draw the consensus.

(All commands in this section should be run within the `demo` directory that is part of the R2R distribution.)

The general form of this command is

> `commandprompt$ ../src/r2r --GSC-weighted-consensus` *input-file*`.sto`
> *output-file*`.sto` 3 0.97 0.9 0.75 4 0.97 0.9 0.75 0.5 0.1

This command is explained in Chapter 4, and the parameters are fully explained in Section 4.4. Also, while it is necessary to generate consensus information so that R2R can draw it, users can write their own routine to generate the consensus data instead of using the `--GSC-weighted-consensus` command; this alternative is explained in Section 4.6.

Given the consensus data generated by the previous command, we run R2R to create a PDF file. The file `demo1.r2r_meta` simply gives the path of `intermediate/demo1.cons.sto`, which is what R2R will use to draw.

```
commandprompt$ ../src/r2r demo1.r2r_meta output-pdf/demo1.pdf
```

Alternately, to create SVG-format output:

```
commandprompt$ ../src/r2r demo1.r2r_meta output-svg/demo1.svg
```

Note: a warning is printed in the output PDF or SVG about misusing R2R to analyze structural conservation, rather than just drawing diagrams. You can disable this warning by adding the `--disable-usage-warning` right after `../src/r2r`

Key points:

- R2R inputs are a subset of Stockholm-format where interleaved lines are not permitted. The Stockholm format is explained more at http://en.wikipedia.org/wiki/Stockholm_format.
- Consensus secondary structure is expressed using brackets (e.g., < and >) in the `#=GC SS_cons` line (e.g., Figure 3.1).
- R2R will infer the consensus sequence and classify covariation.
- Creation of the output requires two UNIX commands.

3.3.2 Some typical customizations for consensus diagrams

In this section we will use some R2R commands to improve the consensus diagram drawn in the previous section. This will entail adding an `#=GC R2R_LABEL` line, which will allow us to label and refer to specific columns in the alignment.

The demo1 drawing in Figure 3.1B has 5′ and 3′ flanking regions that are not conserved according to R2R's definition of conservation. Although it is often desirable to keep such poorly conserved flanking regions in curated alignments, they add little to a diagram. By adding dashes to the `R2R_LABEL` line, we cause R2R to remove those columns in the output (see Figure 3.2).

The first hairpin of the demo1 motif varies in length, and its terminal loop is poorly conserved. The R2R `var_hairpin` command allows us to replace part of the hairpin with an abstract representation of a variable-length hairpin. Similarly, a part of the junction between the hairpins is poorly conserved, and the R2R `var_backbone_range` command replaces this with a line. The line

Figure 3.2 demo1-ii: Some annotation for the consensus diagram

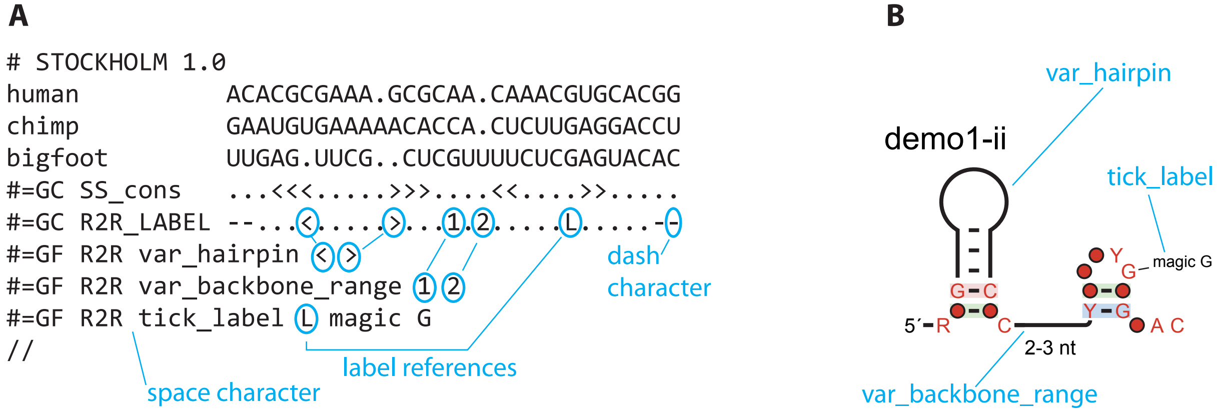

(A) Stockholm-format alignment that includes a labeling line (beginning `#=GF R2R_LABEL`) and associated R2R commands (beginning `#=GF R2R`). Annotations in blue show how R2R commands are associated with a symbol in the labeling line. Annotations also mark the special dash symbol in the `#=GC R2R_LABEL` line that causes the column to be deleted from R2R's drawing. Also it is important to note that a single space character separates all elements of R2R commands. (This text is identical to the file `demo/demo1-ii.sto`.) (B) Raw output of R2R, with annotation (in blue) showing what elements of the drawing were the result of the R2R commands in part A.

is annotated with the range in the number of nucleotides contained in those columns, which is automatically calculated. For both commands, the range is represented by two labels, each of which refer to a column in the `#=GC R2R_LABEL` line. For example, the `var_hairpin` command used the labels $<$ and $>$, each of which refers to a specific column, as shown (Figure 3.2A).

Finally, the drawing uses the `tick_label` command, which labels a specific nucleotide position. In this case, I imagined that a particular G nucleotide has a special biochemical significance in this fictitious RNA structure.

Key points:

- R2R commands refer to specific alignment columns using labels, in the `#=GC R2R_LABEL` line. It is also possible to expand the number of labels beyond simply one symbol (see Section 5.7).
- Columns can be deleted by putting dashes in the `#=GC R2R_LABEL` line.
- R2R has commands to represent variable-length regions of different sorts. For more information on R2R commands related to variable-length regions see Section 5.8.5.
- Specific positions can be labeled with a tick mark using the `tick_label` command.

3.3.3 Other kinds of drawings: single-molecule and skeleton diagrams

Figure 3.3 demonstrates two additional types of R2R drawings. The `.r2r_meta` file is needed to specify both kinds of alternate drawing. The first is drawing of a single molecule whose sequence is present in the alignment. Every tenth nucleotide is numbered by default. These kind drawings are called "`oneseq`" drawings, because they depict one sequence. A real-world example of this kind of drawing is given in Figure 3.4A.

The second kind of drawing is a "skeleton" drawing, which is an outline of the shape. Skeleton drawings can be applied to consensus or to individual sequences, and are useful to give a

Figure 3.3 demo1-iii: Other kinds of drawings

A

```
intermediate/demo1-iii.cons.sto
demo1-iii.sto	oneseq	bigfoot
intermediate/demo1-iii.cons.sto	skeleton-with-pairbonds
```

tab character

B

```
# STOCKHOLM 1.0
human                    ACACGCGAAA.GCGCAA.CAAACGUGCACGG
chimp                    GAAUGUGAAAAACACCA.CUCUUGAGGACCU
bigfoot                  UUGAG.UUCG..CUCGUUUUCUCGAGUACAC
#=GR bigfoot DEL_COLS    .........................-----
#=GC SS_cons             ...<<<.....>>>....<<....>>.....
#=GC R2R_LABEL           --...<.....>...1.2.....Lsssss--
#=GC R2R_XLABEL_bf       ......UUUU.....................
#=GC R2R_XLABEL_bfl      ........U......................
#=GF R2R var_hairpin < >
#=GF R2R var_backbone_range 1 2
#=GF R2R tick_label L magic G
#=GF R2R_oneseq bigfoot outline_nuc bf:U
#=GF R2R_oneseq bigfoot tick_label bfl:U bigfoot UNCG loop
#=GF R2R if_skeleton SetDrawingParam pairBondWidth 0.5pt
#=GF R2R if_skeleton shade_along_backbone s rgb:255,0,0
//
```

C

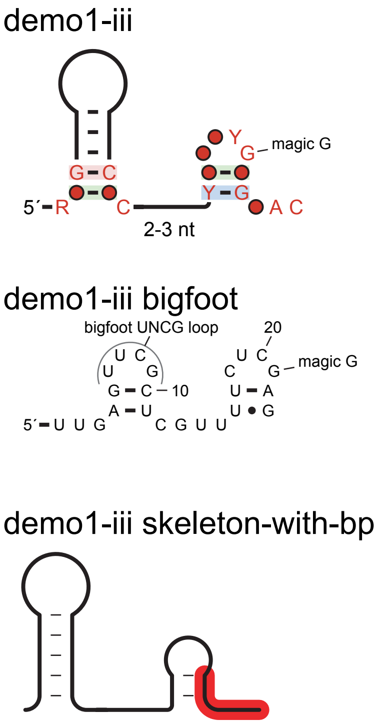

(A) The .r2r_meta file name (demo/demo1-iii.r2r_meta), which directs R2R to draw a regular consensus diagram (first line), a single sequence (oneseq) from the "bigfoot" sequence and a skeleton-style schematic drawing of the consensus. Note that the components in each line are separated by a single tab character. (B) Stockholm-format alignment. (This text comes from the file demo/demo1-iii.sto. Some irrelevant markup has been removed.) The two lines beginning #=GF R2R_oneseq are only processed within oneseq mode (i.e., only for the bigfoot sequence in this example). They refer to column labels with alternate names (bf and bfl) that are introduced using #=GC R2R_XLABEL_bf and #=GC R2R_XLABEL_bfl. In the oneseq case, dashes in the R2R_LABEL line are ignored, but dashes in the #=GR bigfoot DEL_COLS lines cause nucleotides to be eliminated from the drawing. The last line, which contains if_skeleton, is only interpreted in skeleton mode, and it overrides a default drawing parameter to set the basepair bond width to 0.5 points. (C) Raw output of R2R (rearranged to fit the figure space efficiently). From top to bottom is a standard consensus diagram, a single RNA molecule and a skeleton schematic.

Figure 3.4 Other kinds of drawings illustrated with SAM-IV riboswitches

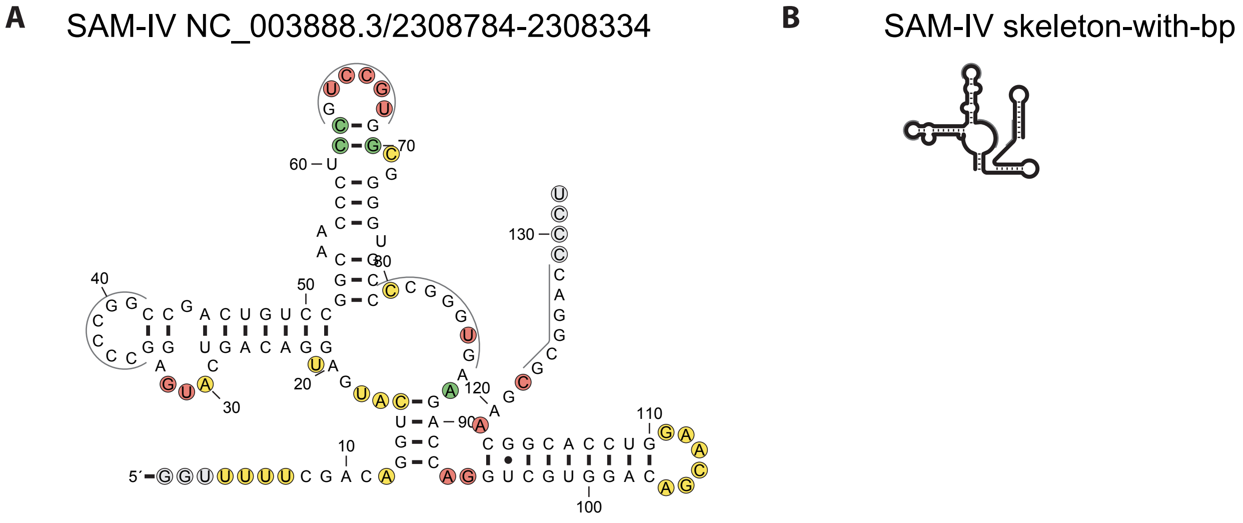

Examples of `oneseq` and skeleton drawings using SAM-IV riboswitches [16]. These examples are built from the files `demo/SAM-IV/SAM-IV.sto` and `demo/SAM-IV/SAM-IV.r2r_meta`. (A) `oneseq` drawing of a SAM-IV riboswitch from *Streptomyces coelicolor* annotated with cleavage data from in-line probing experiments [16]. The annotation of cleavage data used a `#=GR ... CLEAVAGE` line, as explained in Section 5.5.7. Note that this is the raw output of R2R, and some adjustment (in Adobe Illustrator or Inkscape) would be needed to position some of the nucleotide number labels in appropriate places. (B) R2R skeleton drawing of SAM-IV riboswitch consensus. This layout is the same as in Figure 3.10D, but at a reduced scale.

compact summary of the whole structure. Larger skeleton drawings are demonstrated in the files `demo/exceptional/GOLLD.sto` (and `.r2r_meta`) and `demo/exceptional/HEARO.sto` (and `.r2r_meta`). Those files also demonstrate making scaled-down "thumbnail" skeleton drawings. A more realistic example of a skeleton drawing is given in Figure 3.4B.

Key points:

- Fields in the `.r2r_meta` file are separated by tab characters.
- A single RNA molecule can be drawn using the `oneseq` directive in the `.r2r_meta` file.
  - For `oneseq` drawings, nucleotides are removed from the drawing using the `DEL_COLS` sequence-specific line.
  - R2R commands beginning with `#=GF R2R_oneseq bigfoot` are only applied in `oneseq` mode for the sequence `bigfoot`.
- A skeleton-style schematic can be drawn by adding `skeleton-with-pairbonds` (or `skeleton`) in the `.r2r_meta` file. Commands beginning `if_skeleton` are only applied in `skeleton` mode.
- Drawing parameters can be modified with the `SetDrawingParam` command. (This command can also be used in the `.r2r_meta` file.) A list of parameters that can be modified with `SetDrawingParam` is provided in Section 5.5.3.
- Lines beginning `#=GC R2R_XLABEL_`*something* can be used to introduce an extra dimension of labels with the name *something*. See Section 5.7 for details.

3.4 Common error: "One or more pairs is getting broken by one side getting deleted"

This sections explains how to handle a common error when using R2R. R2R defines which nucleotide positions are shown in a consensus based on how frequently they are present in sequences. Sometimes one nucleotide position involved in a base pair is removed from the consensus while the other is retained. This can happen with stems that are loosely conserved. When this arises, R2R will report an error ("one or more base pairs is getting broken..."), and you must choose how to resolve the problem. Figure 3.5 gives an example of this problem, and shows 4 ways to resolve it.

Pairs can also be broken when `var_backbone_range` commands refer to one side of a base pair. In this case, R2R will detect that this command was responsible for the problem (Figure 3.6). If you have variable-length stems, you should use the `var_stem` or `var_hairpin` commands.

Key points:

- Pairs can be broken due to the consensus rules, and Figure 3.5 shows 4 ways to resolve the problem.
- Pairs can also be broken due to variable-length backbones.
- R2R error messages often refer to lines or columns in the input Stockholm-format file. The left-most column is numbered zero. The first line is numbered one.

3.5 The `place_explicit` command

R2R encodes defaults for the layout of RNAs that work in many cases, but often you will want to change this layout. The `place_explicit` command allows you to position nucleotides relative to one another using explicit relative or absolute coordinates (Figure 3.7). The syntax for the command is:

> `place_explicit` *label relative-label place-angle x-relative y-relative x-absolute y-absolute next-angle*

(illustrated with an example in Figure 3.7). The nucleotide at position *label* is positioned relative to the nucleotide at position *relative-label*. The coordinate system of (*x-relative*,*y-relative*) is based on the direction of the nucleotide at *relative-label* rotated by *place-angle* degrees. The x,y coordinates (*x-absolute*,*y-absolute*) are added to this position. Both relative and absolute distances in the `place_explicit` command are specified in drawing units, where 1 drawing unit is equal to the standard internucleotide distance between consecutive nucleotides (by default 0.105 inches). The direction of the nucleotide at *label* is the angle at *relative-label* rotated by *next-angle* degrees.

An optional `f` at the end of the command directs R2R to "flip" the stem (Figure 3.7B,C). Flipping is analogous to applying a twist to the RNA. Where R2R normally positions the $3'$ nucleotide of a base pair to the right of its partner, flipped regions will cause the $3'$ nucleotide to be on the left. Flipping is used only sometimes, but Figure 3.7C shows a typical example where it is useful to avoid crossing stems. A real-world example of this usage is the IMES-4 motif [15] (see `demo/exceptional/IMES-4.sto`).

Section 5.8.2.8 explains the command in more detail.

More advanced usages of `place_explicit` are necessary for the "in-line" layout of complex pseudoknots (Section 3.8.3). I explain more about `place_explicit` commands and their semantics in that section. You can use set the `showPlaceExplicit` variable to `true`, in order to see the

Figure 3.5 demo-breakpair: common error

A

```
# STOCKHOLM 1.0
lemur          AAACUACCCCUAUUGG
blueberry      AAACAUCCCCAU-UGG
smurf          AAGCAACCCCUU-CGG
#=GC SS_cons   ..<<<<....>>>>..
//
```

B

```
ERROR: there was a problem drawing motif "demo-
breakpair": One or more pairs is getting broken by one
side getting deleted (presumably because it's not
conserved), while the other one stays.  Because of
generic process (probably RemoveGaps), and not the
result of any specific command.  Consider using #=GF
R2R keep allpairs.  (Note: another explanation is that
you've used #=GF R2R var_backbone_range on columns
that includes a pair.   (text columns in alignment
[16,25], left=keep,right=chuck, left context=AARC)
```

C

```
# STOCKHOLM 1.0
lemur          AAACUACCCCUAUUGG
blueberry      AAACAUCCCCAU-UGG
smurf          AAGCAACCCCUU-CGG
#=GC SS_cons   ..<.<<....>>.>..
//
```

D

```
# STOCKHOLM 1.0
lemur          AAACUACCCCUAUUGG
blueberry      AAACAUCCCCAU-UGG
smurf          AAGCAACCCCUU-CGG
#=GC SS_cons   ..<<<<....>>>>..
#=GC R2R_LABEL ...-........-...
//
```

E

```
# STOCKHOLM 1.0
lemur            AAACUACCCCUAUUGG
blueberry        AAACAUCCCCAU-UGG
smurf            AAGCAACCCCUU-CGG
#=GC SS_cons     ..<<<<....>>>>..
#=GC R2R_LABEL   ...p........p...
#=GF R2R depair p
//
```

F

```
# STOCKHOLM 1.0
lemur          AAACUACCCCUAUUGG
blueberry      AAACAUCCCCAU-UGG
smurf          AAGCAACCCCUU-CGG
#=GC SS_cons   ..<<<<....>>>>..
#=GC R2R_LABEL ...p........p...
#=GF R2R keep p
//
```

G

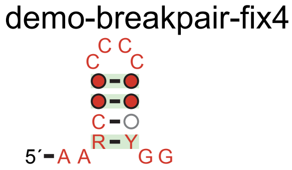

(A) input Stockholm file (`demo/demo-breakpair.sto.err`). Column numbers are labeled, with the left-most column having the number zero. (This is the numbering system used by the text editor program "Emacs".) (B) output of R2R with an error. The referenced column numbers are circled in blue. (C) one resolution: since the base pair is not well conserved, remove it from the `SS_cons` line. (You should only do this if you truly believe, on reflection, that the pairing is not biological.) (file `demo/demo-breakpair-fix1.sto`) (D) typical resolution: the base pair is not that well conserved anyway, so don't show its nucleotides in the consensus diagram at all. However, retain the pairing in the alignment, since the pair is sometimes formed. The nucleotides in the pairing are removed from the drawing by putting dashes in the `R2R_LABEL` line. (file `demo/demo-breakpair-fix2.sto`) (E) another resolution: break the pair in R2R, making it a bulge. (file `demo/demo-breakpair-fix3.sto`) You can also make R2R apply this solution automatically to all otherwise-broken base pairs by adding `#=GF R2R SetDrawingParam autoBreakPairs true` to the file. This is useful when you want to draw an RNA quickly. (F) another resolution: keep both sides of the pair. The nucleotide that would normally have been deleted is drawn with a circle that uses a gray line, rather than the normal black line. (file `demo/demo-breakpair-fix4.sto`) (G) The drawing resulting from part F.

Figure 3.6 demo-breakpair 2: Another cause

A

```
# STOCKHOLM 1.0
human           ACACGCGAAA.GCGCAA.CAAACGUGCACGG
chimp           GAAUGUGAAAAACACCA.CUCUUGAGGACCU
bigfoot         UUGAG.UUCG..CUCGUUUUCUCGAGUACAC
#=GC SS_cons    ...<<<....>>>....<<....>>.....
#=GC R2R_LABEL .......................1...2...
#=GF R2R var_backbone_range 1 2
//
```

B

```
ERROR: there was a problem drawing motif
"demo-breakpair-varlen": One or more pairs
is getting broken by one side getting
deleted (presumably because it's not con-
served), while the other one stays.
Because of command on line #7.  Consider
using #=GF R2R keep allpairs.  (Note:
another explanation is that you've used
#=GF R2R var_backbone_range on columns that
includes a pair.   (text columns in align-
ment [33,40], left=keep,right=chuck, left
context=nR-nCnCnn-Y) (text columns in
alignment [34,39], left=keep,right=chuck,
left context=R-nCnCnn-Yn)
```

(A) input Stockholm file (`demo/demo-breakpair-varlen.sto.err`). (B) output of R2R with an error. The line number with the problematic command is circled in blue, and corresponds to the `var_backbone_range` command.

`place_explicit` commands in the context of the structure. This is demonstrated in the same section (Section 3.8.3).

3.6 Multistem junctions and turning internal loops

Multistem junctions are a structural element within a RNA secondary structure in which a loop includes more than two base pairs (e.g., see Figure 3.8). This section can be skipped if you are drawing RNAs that don't have multistem junctions. However, the material in this section is also sometimes useful for turning the direction of the stem at internal loops (see Figure 3.14 for an example of an internal loop).

There are three ways to draw multi-stem junctions with R2R:

- The junction is laid out perfectly on a circle, and stems are allowed to go in arbitrary directions. This is the default. It usually looks acceptable (except when nucleotides clash), although I think it's typically not the most aesthetic solution.
- Manual layout using `place_explicit` commands.
- Automated solutions that attempt to position the junction on a circle as well as possible, but subject to constraints on the directions of the stems. This allows for orthogonal stems, a design feature that I think tends to result in more attractive layouts.

3.6.1 Simple circular layout

The default circular layout for multistem junctions is demonstrated in Figure 3.8. This is the layout that R2R uses if you do not give R2R any instructions on how to draw the multistem junction.

Figure 3.7 The `place_explicit command`

A

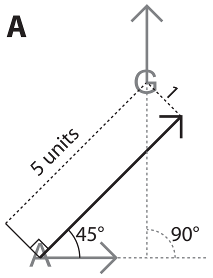

B

```
# STOCKHOLM 1.0
biologist      AACCCUUCGGAAACGGAGUAA
chemist        AACCCUUGCUUCGGCGAGUAA
physicist      AUCCCAUUUCUUGAAGAGAAA
#=GC SS_cons   <<...>><<....>>......
#=GC R2R_LABEL .......a.......b..c..
#=GF R2R place_explicit a a-- 0 1 0 0 0 0 f
#=GF R2R place_explicit b b-- +45 1 0 0 0 +90 f
#=GF R2R place_explicit c c-- -45 1 0 0 0 -90
//
```

C

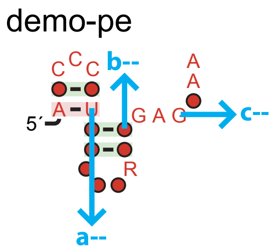

D

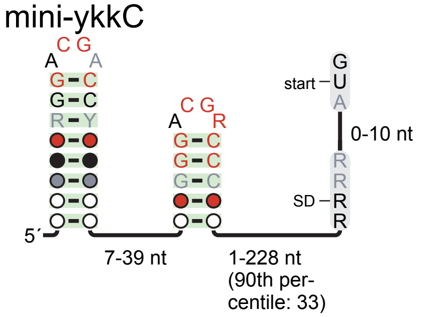

(A) Schematic of the positioning system in the `place_explicit` command. The diagram illustrates the effect of the command `place_explicit G A -45 5 -1 0 0 -90`, which defines how the nucleotide labeled G is positioned depending on the position of the nucleotide A. The direction of the backbone at the A (gray array pointing right) is rotated -45 degrees to form the black arrow. The position proceeds 5 drawing units along the black arrow, and -1 unit orthogonally to the black arrow. This is where the G is positioned. The direction of the backbone of the G (gray arrow pointing up) is taken by adding -90 to the direction of the backbone at the A. The two zeroes mean that no absolute positioning is used. One drawing unit is equal to the distance between consecutive nucleotides along the backbone. (B) Hypothetical Stockholm alignment to demonstrate `place_explicit` command (contents of the file `demo/demo-pe.sto`). Note that the `f` at the end of the first and second `place_explicit` commands directs R2R to "flip" the backbone such that the 3′ side of a base pair is on the left of the 5′ side, instead of the usual right side. The second flipping command flips the orienation back to the default for the 3′ tail of the hypothetical motif. Synthesizing an appropriate joke based on the organisms from which the hypothetical RNA sequences were taken is left as an exercise for the reader. (C) Annotated raw output of R2R when run on the alignment in part B. Nucleotide positions that were the referent of `place_explicit` command (i.e., `a--`, `b--` and `c--`) have a blue arrow indicating the direction of the backbone at that position. (D) Raw R2R output of the mini-*ykkC* motif (`demo/22/mini-ykkC.sto`) [13], which uses a `place_explicit` command to change the direction of the `var_backbone_range` just before the SD sequence.

Figure 3.8 Simple circular layout of multistem junctions

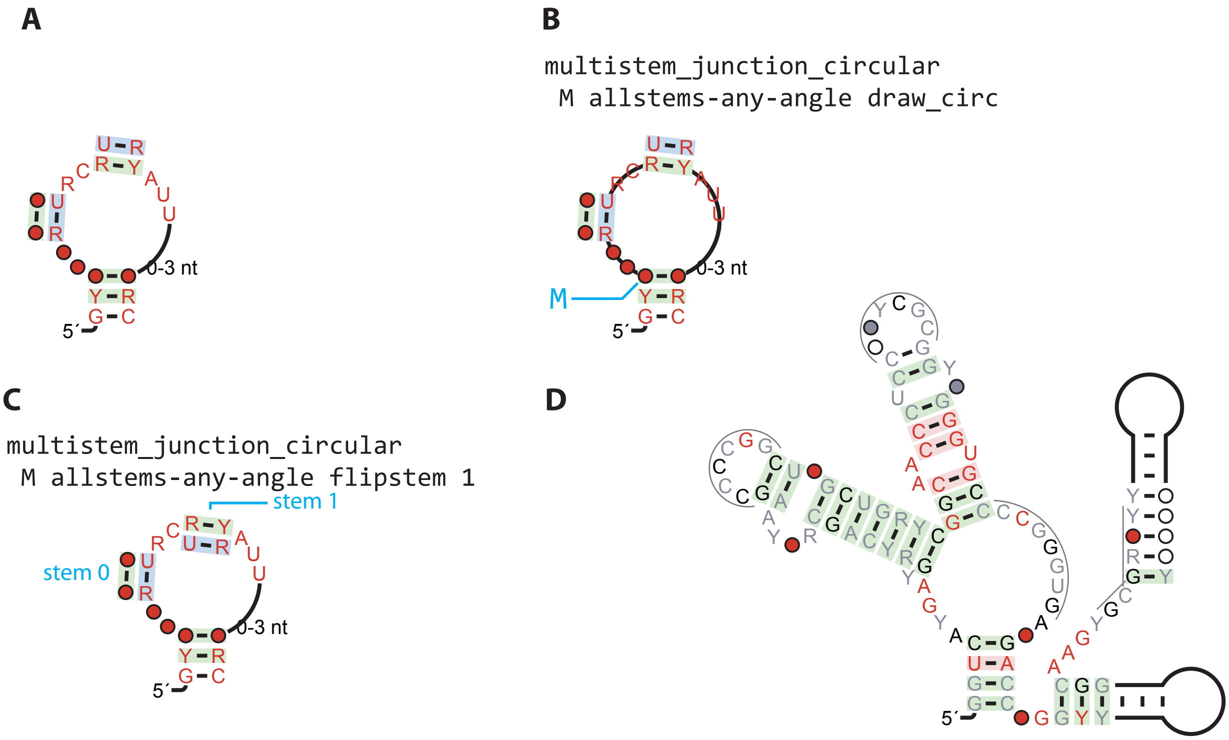

(A) Default layout of a hypothetical three-stem junction that is modeled on a multistem junction in HEARO RNA [15]. This output is based on the files `demo/demo-multistem.sto` and `demo/demo-multistem.r2r_meta`. (B) In the default layout, nucleotides along the multistem junction are positioned on a circle. The R2R command shown here directs R2R to draw this circle. The command refers to position “`M`”. Position `M` is labeled, and is the left nucleotide of the base pair that encloses the whole multistem junction. (Note: the command should be one line, but is displayed on multiple lines in this figure so it fits the page.) (C) Stems can be flipped so that they are directed into the multistem junction. This can save space when the multistem loop is larger than one of the stems. The stems are numbered starting at zero. The enclosing stem does not have a number, and cannot be flipped. (D) The SAM-IV riboswitch [16] is drawn using the default layout of its multistem junction. (Note: the gray lines correspond to pseudoknots. Drawing of pseudoknots are explained in a later section of this tutorial.)

Figure 3.9 Manual layout of multistem junctions

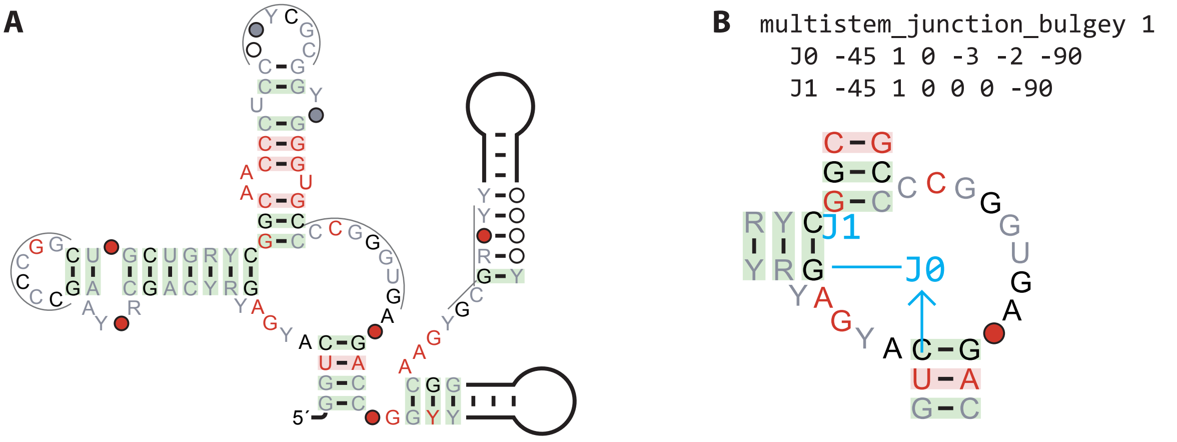

(A) The published layout of SAM-IV riboswitches [16]. Layout of the multistem junction uses the `multistem_junction_bulgey` command. This drawing is based on the file `demo/SAM-IV/SAM-IV.sto`, in `multistem=original` mode. This mode is entered in the `.r2r_meta` file using the specification `define multistem original`. (Note: at the time at which SAM-IV riboswitches were drawn, manual layout was the only option for drawing multistem junctions. Also, the gray lines are for pseudoknots, as described in Section 3.8.2.) (B) R2R command to draw the multistem junction, and larger view of the multistem junction. The junctions called "`J0`" and "`J1`" are labeled. There are no nucleotides within junction `J1`.

3.6.2 Manual layout of multistem junctions

Manual layout of multistem junctions can be accomplished by positioning the stems using `place_explicit` commands, and directing the layout of the single-stranded regions using the `bulge` command. Since the use of these commands would result in many labels to identify the various positions, R2R has the `multistem_junction_bulgey` command, for which only one label needs to be defined.

In the `multistem_junction_bulgey` command, the stems within the multistem junction are positioned using functionality similar to `place_explicit` commands. The junctions are then positioned automatically between the stems by positioning them on circles (equivalent to the `bulge` command). Figure 3.9 shows an example.

Junctions are identified using the symbols `J0`, `J1`, `J2`, etc. Junction `J0` identifies the junction between the enclosing stem and the next 5′-most stem. Junction `J1` identifies the junction between this next 5′-most stem, and the next stem to that (i.e., the immediately 3′ stem). Positioning of `J0` is equivalent to a `place_explicit` command that positions X relative to Y, where X is the left nucleotide of the stem on the 3′ side of the junction `J0` and Y is the left base-paired nucleotide of the stem on the 5′ side of the junction `J0`. For junctions `J1`, `J2`, etc., Y is the *right* nucleotide of the stem on the 5′ side of the junction. An equivalent viewpoint is that X and Y are the base-paired nucleotides immediately 3′ (X) and 5′ (Y) to the nucleotides within the junction. This is also illustrated in Figure 3.9.

The `multistem_junction_bulgey` command also provides alternate layouts for the bulges, such as flipping the direction of the bulge, and some linear layouts for the junctions. It is also possible to position the 5′ base-paired nucleotides in all stems relative to the enclosing stem, rather than to the previous stem by using `J1/base` in place of `J1`. These variations are described in Section 5.8.3.1.

3.6.3 Automated multistem junction layout with direction constraints

R2R provides the ability to automatically determine a layout of a multistem junction, given user-specified directions in which each stem must go. This functionality requires that CFSQP is available (see Chapter 2).

This automated layout facility is refered to as the `_solver` commands in this manual, and is applied with one of the following three related commands:

- `multistem_junction_circular_solver`
- `multistem_junction_bulgecircley_solver`
- `multistem_junction_bulgecircleynormals_solver`

All `_solver` variants use a very similar syntax and framework. They are explained in full detail in Section 5.8.3.2, but for now we will see the most essential aspects of the commands.

Examples of the `_solver` commands are given in Figures 3.10, 3.11, 3.12 and 3.13. The command begins with a `_solver` command, then specifies a label. As with previous multistem junction commands, the label denotes the left nucleotide in the base pair that encloses the multistem junction. Then, then properties of each stem in the multistem junction are written. The enclosing stem is named `s0`, then each stem from $5'$ to $3'$ are labeled `s1`, `s2`, ... (see Figure 3.10). The names for the stems are different from those of multistem junction commands described earlier in this tutorial. In particular, the enclosing stem can be explicitly referenced (as `s0`).

For each stem, the desired angle of that stem is given, in a coordinate system defined by the enclosing stem (Figure 3.10B). The point at which the circle should intersect the inner base pair of each stem is also specified. Useful values are `m` (intersects the `m`idpoint of the base pair), `l` (intersects the center of the `l`eft nucleotide in the base pair), `r` (intersects the `r`ight nucleotide in the base pair) and `ai` (`a`utomatically solve the `i`ntersection).

To find a good layout of a particular multistem junction, it is often necessary to play with several formulations, involving different stem angles and solvers. One strategy to finding good directions for the stems is to use the default circular positioning of the multistem junction to find directions that fit the circle perfectly, then consider nearby angles that are multiples of 90 degrees, or have other pleasing characteristics. See Figure 3.11 for an example. As noted above, the starting point for the circle-intersection point of each base pair can sometimes have an effect on the solution.

Variable-length backbones (introduced using the `var_backbone_range` command) can help to improve aesthetics of drawings, as the solver is permitted to change the length of the lines to improve the circularity of the layout. This additional degree of freedom can be helpful. Of course, some multistem junctions do not have variable-length regions, so this tactic cannot always be used.

Additional examples illustrating aspect of the `_solver` commands are shown in Figures 3.12 and 3.13.

Note: the starting point for the intersection can be supplied for automatic inference, and this sometimes assists the solution, as the problem that the computer has to solve is highly non-linear, and the computer can easily get stuck in local minima. See Section 5.8.3.2 for details on various initial values that can be set. For example, `ai=0` means start with the intersection at the left nucleotide and `ai=1` means start at the right nucleotide of the base pair. Any number from 0 to 1 can be given, so `ai=0.5` means start at the midpoint. The circle intersection parameter is only relevant to `multistem_junction_circular_solver`; the `multistem_junction_bulgecircley...` commands try to have both nucleotides on the circle. However, other parameters are relevant to

Figure 3.10 Automated layout of multistem junctions

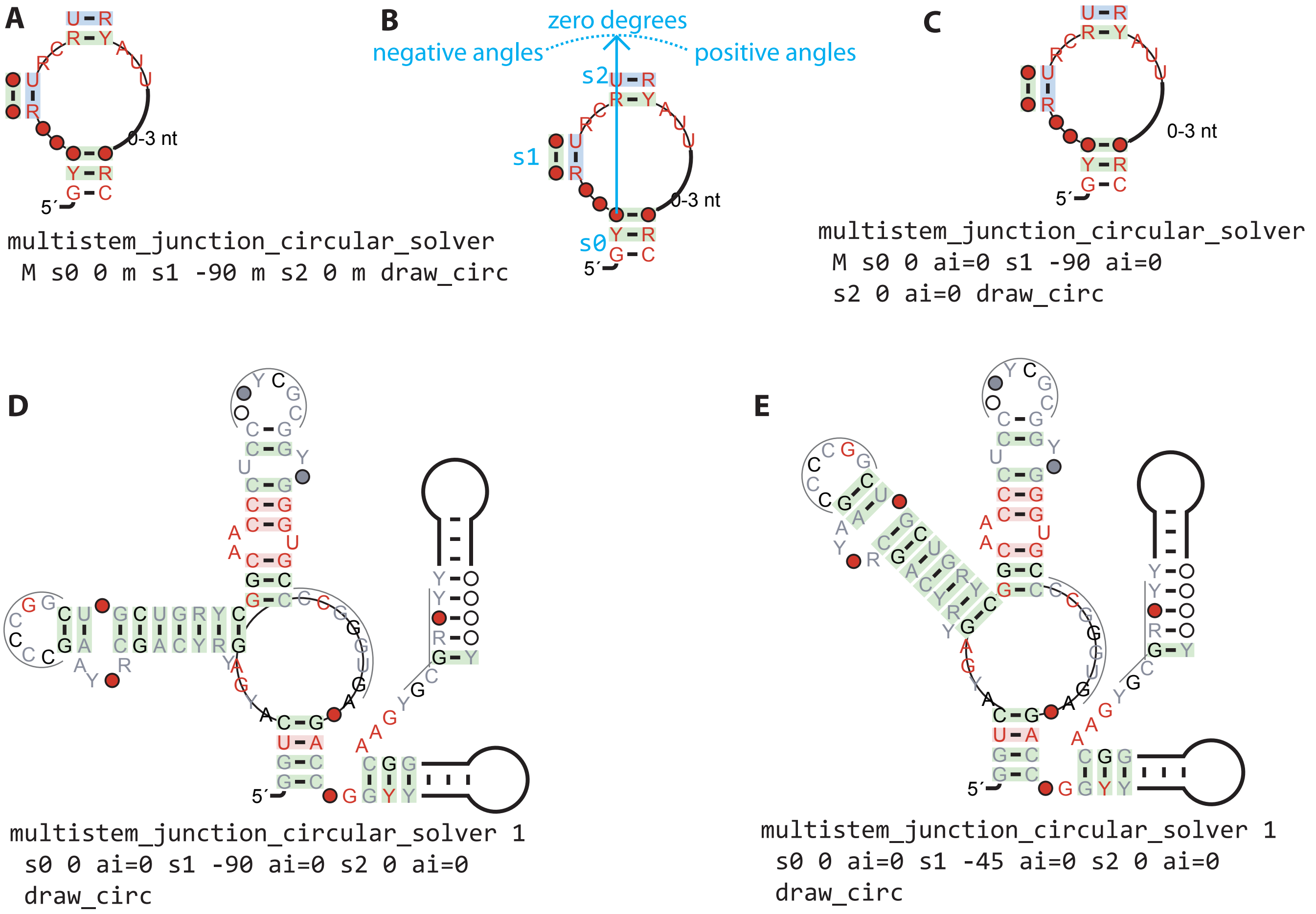

(A) Layout of the demo structure used in previous figures, using the solver. The solver command is given below the drawing. The lower case "m" parameters say that the circle should intersect in the midpoint (m) of stems. Note that the length of the variable-length region (marked as "0-3 nt") is adjusted by the solver to optimize the circular layout. (The drawing is based on the file `demo/demo-multistem.sto`, with `solver1=1`.) (B) Illustration of stem numbers and directions from the diagram of part A. The enclosing stem is `s0`, then the stems are number `s1`, `s2` from 5′ to 3′ around the multistem junction. The stem `s0` defines the angle zero, as indicated. As usual within R2R, positive angles are in the clockwise direction, and negative angles are counter-clockwise. Thus, `s1` is oriented in the direction `-90` (going to the left), while `s2` is in the direction `0` (up the page). If the enclosing stem (stem `s0`) were rotated, the angles of stems `s1` and `s2` would also rotate to be the same, relative to the enclosing stem. The angle of the enclosing stem (stem `s0`) can be changed, which has the effect of rotating the entire picture. This is purely for convenience, and is illustrated in Figure 3.11. (C) A variation on the drawing in part A. The `ai=0` parameters directs R2R to automatically decide on the intersection point with the circle for each stem. This leads to a circle that better goes through each nucleotide. (D) Drawing of the SAM-IV riboswitch [16] using the solver. In this case, the bulged `Y` within stem `s1` is very close to the `A` in the junction between stems `s0` and `s1`. The positions of these nucleotides could be adjusted in Adobe Illustrator of Inkscape. Alternately, the stem `s1` could be rotated to avoid this problem, as demonstrated in the next sub-figure. (E) Drawing of the SAM-IV riboswitch with an alternate angle for stem `s1`.

Figure 3.11 Automated layout of multistem junctions: unusual stem directions

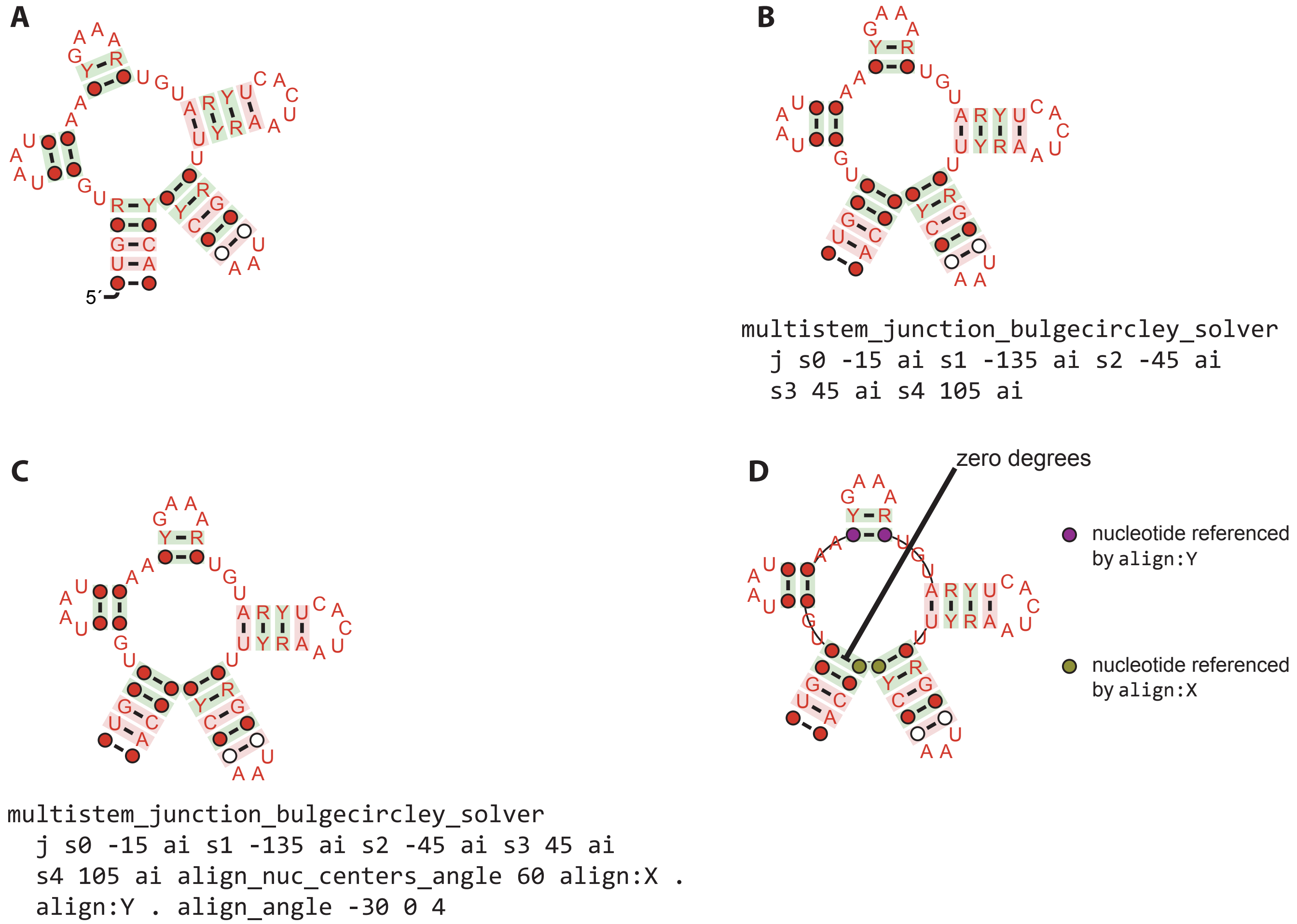

This figure illustrates the strategy of using the default circular layout to suggest aesthetic stem directions. In this case, I was not able to find directions that are multiples of 90 or even 45 that worked well. This example is based on a multistem junction in the *manA* motif [17] (see `demo/104/manA.sto` for the full motif). (A) Default circular layout of this motif. (This drawing is derived from the file `demo/demo-multistem-manA.sto`.) (B) Layout using solver, with stem directions that roughly match the default orientations in part A. Stem `s0` is rotated 30 degrees clockwise in this figure. The directions of stems `s0` and `s4` are symmetric about the vertical axis, which results in an aesthetic design. (Note: R2R does not draw the 5′ marking since the 5′ nucleotide's direction is not orthogonal to the X or Y axis.) (C) Alignment constraints are added to the layout of part B. `align_angle -30 0 4` dictates that the stems `s0` and `s4` should be vertically aligned. (The alignment angle `-30` is used because the enclosing stem is rotated by 30 degrees; see part D.) The constraint `align_nuc_centers_angle 60 align:X . align:Y .` forces the midpoint of the nucleotides defined by `align:X` to be horizontally aligned with the midpoint of the `align:Y` nucleotides. This alignment further improves the symmetry. Note that R2R cannot aesthetically satisfy any arbitrary constraint, and so stems `s1` and `s3` cannot be vertically aligned in this layout. (D) The drawing of part C is annotated. The nucleotides that were aligned are drawn in alternate colors as shown. The `align:X` nucleotides were vertically aligned with each other when their stems (`s0` and `s4`) were aligned. Zero degrees is defined as the direction of stem `s0` (as shown), so the vertical direction is `-30` degrees. The solver directives `draw_circ` and `draw_zero_degrees` were added to draw the multistem junction circle, and the zero degrees mark, and `nuc_color` was used to color the `align:X` and `align:Y` nucleotides.

Figure 3.12 Automated layout of multistem junctions: additional example

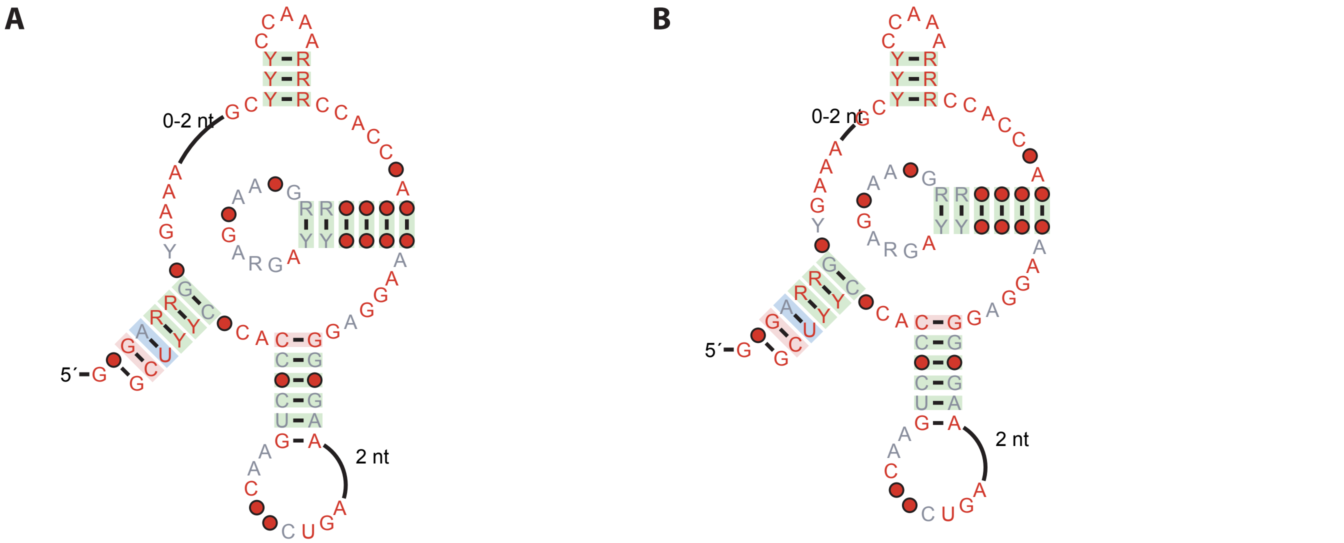


```
multistem_junction_circular_solver
 j s0 0 m s1 -45 m s2 +45 m
 s3 +135 m flipstem 2
```

```
multistem_junction_bulgecircleynormals_solver
 j s0 0 m s1 -45 m s2 +45 m
s3 +135 m flipstem 2
```

This figure illustrates an additional example of a multistem junction drawing, based on a multistem junction found in IMES-1 RNAs [15]. (The drawing is based on the file `demo/demo-multistem-IMES-1.sto`.) (A) Layout of the given command. (B) Layout a different _`solver` command, as shown.

Figure 3.13 Automated layout of multistem junctions: starting point is important

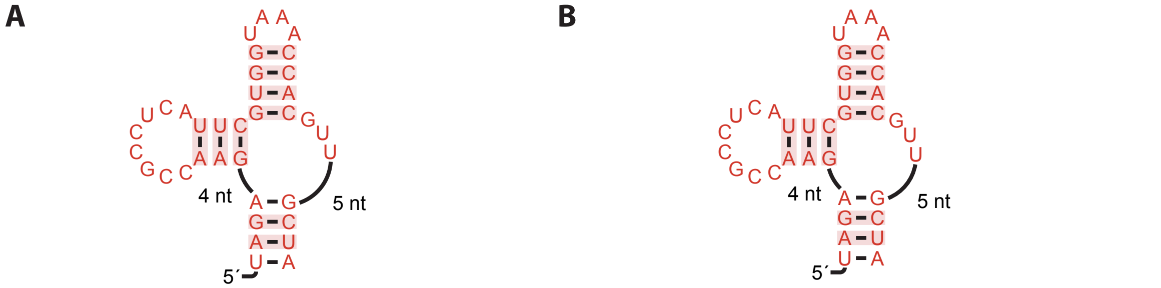


```
multistem_junction_circular_solver
 J s0 0 ai s1 -90 ai s2 0 ai
 align_stem_horiz 0 2
```

```
multistem_junction_circular_solver
 J s0 0 ai s1 -90 ai=0 s2 0 ai=1
 align_stem_horiz 0 2
```

This drawing is based on a multistem junction in HEARO RNA [15] that is different from the earlier multistem junction shown. (The drawing is based on the file `demo/demo-multistem-HEARO2.sto`.) (A) Layout of the given command, with `ai` parameters using the default midpoint as the initial point for the optimization search. (B) Layout of the given command, with `ai=0` and `ai=1` specifying alternate starting points. The resulting layout is slightly different, and I think a bit better.

the bulgecircley variant commands. You can also add try_harder to the multistem_junction_-circular_solver command to improve it's ability to find a good global optimum (details in Section 5.8.3.2).

3.6.3.1 Solving multistem junctions can be slow

On even modern machines, the computer can take a significant amount of time to solve the problem. Often the solver goes through a period of very gradually improving the solution, and many iterations are required to reach an optimal solution—and the difference in the ultimate solution is noticeable. Therefore, I have conservatively set the solver to use up to 100,000 iterations. You can decrease this number with an R2R command like SetDrawingParam solverMaxIters 50000.

I have also implemented a "caching" strategy, in which solutions to problems solved by CFSQP are store in a file that is associated with the .r2r_meta input file. These files end in the extention .solver-cache. If you re-run R2R, but do not change anything related to the multistem junction, the solution will be re-used. By deleting the .solver-cache file, you can force a re-computation.

Note: R2R is designed to detect subtle changes that can change the problem, and R2R will recompute the solution in these cases. For example, if you change the value of internucleotideLen using the SetDrawingParam command, this changes a parameter in the multistem junction Non-Linear Program, even though it is an indirect effect.

3.6.4 Internal loops

Internal loops and bulges are unpaired regions that interrupt stems. It is often desirable to turn the direction of the stem at these loops for two main reasons. First, bulges and highly asymmetric internal loops can have a distorted look due to having too many nucleotides on one side. A turn can give more space to the longer side, improving the clarity and aesthetic quality of the drawing. Second, turning of stems can resolve problems with other regions of the structure that might overlap, use space inefficiently, or otherwise be undesirable.

R2R has a special command for turning the direction of the stem at internal loops or bulges. This command restricts turning to +90 or -90 degrees, however. The command, turn_stem_at_-internal, is demonstrated in Figure 3.14, and documented in detail in Section 5.8.2.5.

The turn_stem_at_internal command is limited both in the angles it can handle, and in the layout policy. You can use the _solver commands that are designed for multistem junctions on internal loops or bulges. An internal loop is simply a multistem junction with only 2 stems, for the purposes of these commands. The application of _solver commands to internal loops is demonstrated in Figure 3.14.

Note that R2R does not distinguish between internal loops and bulges, as RNA biologists often do. For R2R, a bulge is simply an internal loop with zero nucleotides on one side.

3.7 Modular structures

I define modular structures as sub-structures of an RNA that are sometimes, but not always, present in a conserved RNA structure. Many RNAs, for example, have stems that are sometimes present. To draw a consensus, it is often desirable to reflect these modular structures, and their relative frequencies. As with other frequencies, R2R weights sequences using the GSC algorithm, and calculates the weighted frequency of the set of sequences that do have the modular structure. (See Chapter 4 for details.)

Figure 3.14 An internal loop with a 90-degree turn

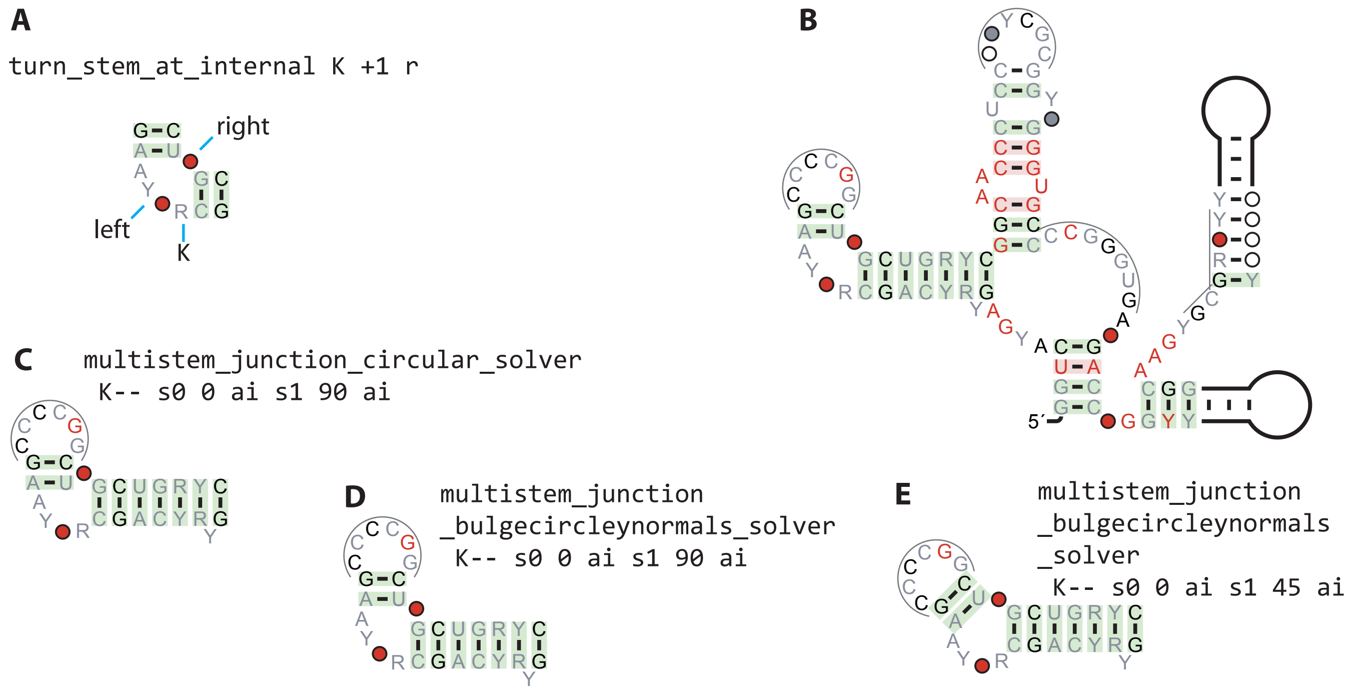

This drawing is based on SAM-IV riboswitches [16, 13], in the file `demo/22/SAM-IV.sto`. (A) An R2R command to turn an internal loop in SAM-IV riboswitches. The label `K` refers to the 5′-most nucleotide within the 5′ part of the internal loop, as shown. (You can also use the 3′-most part of the 3′ part of the internal loop.) The stem can be turned 90 degrees clockwise or counter-clockwise. The value `+1` (as shown) is clockwise, while `-1` would be counter-clockwise. R2R will decide the layout on one side of the internal loop. The value `r` will make R2R use the `r`ight side (i.e. 3′ side) for positioning, while `l` is for the `l`eft side. (B) The turned internal loop in the context of the full SAM-IV consensus. (C-E) Turning the internal loop using `_solver` commands. Since the `_solver` commands require the label to be the left nucleotide of the enclosing base pair of the internal loop, we use the label `K--`.

Figure 3.15 Modular structures

A

```
# STOCKHOLM 1.0
one                           GAGAAA.UCAACCCUUGGGGGAGCA
two                           AGUUCG.CUAAUCCUAGGAGGAGCA
three                         GGGAAACCCAA........GGAGCA
four                          AGCAAC.CUAA........GGAGCA
#=GC SS_cons                  <<.....>>..<<....>>......
#=GC R2R_LABEL                .........................
#=GC SUBFAM_LABEL_TERM        ..xxxxx..................
#=GC SUBFAM_LABEL_OPT         .............x...........
#=GC SUBFAM_GNRA_R2R_LABEL    -.......-----------------
#=GC SUBFAM_UNCG_R2R_LABEL    -.......-----------------
#=GC SUBFAM_OPT_R2R_LABEL     -----------.......------

#=GF SUBFAM_REGEX_PRED HAS_GNRA TERM G[A-Z][AG]A
#=GF SUBFAM_REGEX_PRED HAS_UNCG TERM U[A-Z]CG
#=GF SUBFAM_REGEX_PRED FOUR TERM ^[.]*[A-Z][A-Z][A-Z][A-Z][.]*$
#=GF SUBFAM_PERL_PRED GNRA return $predValue{HAS_GNRA} && $predValue{FOUR};
#=GF SUBFAM_PERL_PRED UNCG return $predValue{HAS_UNCG} && $predValue{FOUR};
#=GF SUBFAM_REGEX_PRED OPT OPT [A-Z]

#=GF SUBFAM_GNRA_R2R no5
#=GF SUBFAM_GNRA_R2R set_dir pos0 -90
#=GF SUBFAM_UNCG_R2R no5
#=GF SUBFAM_UNCG_R2R set_dir pos0 -90
#=GF SUBFAM_OPT_R2R no5
#=GF SUBFAM_OPT_R2R set_dir pos0 -90
//
```

B

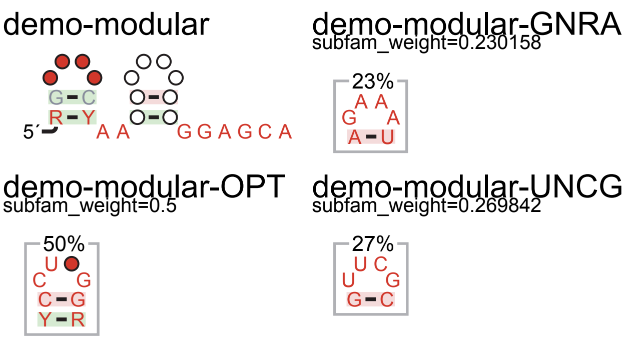

C

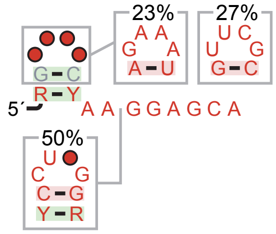

(A) The Stockholm alignment and R2R drawing commands for a hypothetical modular structure. This is the file `demo/demo-modular.sto`. (B) Raw output of R2R. To extract the Stockholm file for the predicate `GNRA`, for example, the command `perl ../src/SelectSubFamilyFromStockholm.pl intermediate/demo-modular.cons.sto GNRA > intermediate/demo-modular-GNRA.sto` was used, followed by inference of the consensus using `../src/r2r --GSC-weighted-consensus intermediate/demo-modular-GNRA.sto intermediate/demo-modular-GNRA.cons.sto 3 0.97 0.9 0.75 4 0.97 0.9 0.75 0.5 0.1`. The file `intermediate/demo-modular-GNRA.cons.sto` was included in `demo-modular.r2r_meta`. Similar commands were run for the `UNCG` and `OPT` predicates. (C) Integration of the modular structures into a consensus diagram. This drawing was created in Adobe Illustrator using the output of R2R shown in part B.

Figure 3.15 demonstrates the drawing of two modular substructures in the context of a simple, hypothetical motif. It covers hairpins that are sometimes, but not always, present. It also covers a loop that has distinct structures that are possible, namely the stable GNRA or UNCG tetraloops. The figure legend gives the commands that would be run to process files with modular structures.

The basic idea for processing modular structures in R2R is (1) to define a predicate that determines which sequences exhibit a given substructure (e.g., which sequences have the optional hairpin), (2) extract those sequences into a new `.sto` file using a Perl script that is part of R2R and (3) draw the new file. Predicates can be defined using either regular expressions or using Boolean operations using a subset of the Perl programming language.

Regular expressions are a mathematical formalism for describing sequence patterns [1]. Some examples are given in Figure 3.15. To implement these regular expressions, I used Perl, and consequently R2R uses the Perl syntax to specify regular expressions. To learn about all the capabilities of regular expressions in Perl, one good Web site is `http://www.perl.com/doc/manual/html/pod/perlre.html`.

Regular expression predicates operate on subsequences in columns defined by lines of the form `#=GC SUBFAM_LABEL_...`. For example, `#=GC SUBFAM_LABEL_TERM` would define columns that can now be referred to as "`TERM`".

Here are some examples of regular expressions that illustrate features relevant to RNA. For four columns with no gaps, `G[A-Z][AG]A` defines the GNRA tetraloop. The text in square brackets (`[` and `]`) define a set of symbols. `[A-Z]` is the set of upper-case letters, while `[AG]` defines the symbols `A` or `G`. A period ("`.`") is a special symbol that stands for any character, so in order to look for a gap character you should enter `[-.]`.

A few other special symbols are often relevant. The caret symbol "`^`" (shift-6 on a standard US keyboard) stands for the beginning of a subsequence, while the dollar sign "`$`" represents the end of a subsequence. The asterisk "`*`" represents zero or more occurrences of the previous symbol. Thus, the expression `^[-.]*$` matches all subsequences that consists only of gap characters.

R2R also provides Boolean operations that can combine the values of other predicates. These predicates also exploit Perl, and therefore inherit a Perl-like syntax. In these predicates, the expression `$predValue{GNRA}`, for example, would evaluate to true if the predicate named `GNRA` is true for a given sequence. Two ampersands (`&&`) represents the Boolean "and" operation, which evaluates to true if the two sub-expressions are true. So, `$predValue{GNRA} && $predValue{FOUR}` is true if the predicates `GNRA` and `FOUR` are both true. Similarly, two pipe symbols (||) denote the Boolean "or" operation, which evaluates to the true if *either* of the sub-expressions are true. The exclamation mark `!` is the Boolean not operation, so the expression `!$predValue{GNRA}` represents all sequences that do *not* match the `GNRA` predicate. Warning: if you use Boolean predicates, you must define predicates they use within `$predValue` earlier in the `.sto` file, since R2R will evaluate predicates in the order in which they appear in the `.sto` file.

The script `SelectSubFamilyFromStockholm.pl` evaluates predicates and creates a new `.sto` file with the subset of sequences. It also modifies which commands are present in the file, in order to use commands that are appropriate to drawing the modular structure. All lines beginning with #=GF R2R or #=GC R2R are by default removed. At the same time, for example with the predicate `GNRA`, lines beginning #=GF SUBFAM_GNRA_R2R are changed to #=GF R2R so that they now become processed. Similarly, lines beginning `#=GC SUBFAM_GNRA_R2R...` are changed to `#=GC R2R...`.

Chapter 6 gives further details on the commands.

3.8 Pseudoknots

Pseudoknotted RNAs typically lead to confusing drawings when drawn with the default layout of R2R. Therefore, to help you to plan the final layout better, the first thing to do with an RNA structure that has a pseudoknot is usually to make R2R ignore the pseudoknot, which can be accomplished with the `ignore_ss_except_for_pairs` command. I give an example of this command in the next section, since it leads naturally to a "callout" style of pseudoknot drawing.

3.8.1 A note on the representation of pseudoknotted secondary structures within Stockholm files

R2R uses a non-standard method for writing consensus secondary structures for motifs with pseudoknots. To describe pseudoknots, in addition to the standard `#=GC SS_cons` line, there can be other lines of the form `#=GC SS_cons`X for any string X (typically, I call them `#=GC SS_cons_1`, `#=GC SS_cons_2`, ...). In the pseudoknot examples in this section, you will see `#=GC SS_cons_1` used. GOLLD RNA [15] has 5 pseudoknots (see `demo/exceptional/GOLLD.sto`), and uses up to `#=GC SS_cons_5`. When you use extra `#=GC SS_cons_...` lines, I recommend that they each only contain only one stem (possibly with bulges/internal loops), and not more complex structures. R2R is best tested with this convention.

I find this representation of pseudoknots within Stockholm files to be convenient, because all `#=GC SS_cons` lines are interpreted in the same way.

3.8.2 Pseudoknots drawn with callouts

Figure 3.16 gives an example of an RNA with a pseudoknot that is drawn using a callout style. This style depicts the pseudoknot pairing in a "callout" (Figure 3.16C) that also allows R2R to shade covarying base pairs. To achieve this, we consider the pseudoknot as a modular structure, and make R2R draw it separately. Modular structures were explained in Section 3.7. Their application to pseudoknots is somewhat simpler, since all sequences presumably have the pseudoknot, so there is no need to define any regular expressions.

The line `#=GF SUBFAM_PERL_PRED pknot return 1;` (e.g., see Figure 3.16A) defines the `pknot` "modular" structure as applying to all sequences. R2R commands and `#=GC` lines beginning with `SUBFAM_pknot_` then define commands that should be run to generate the `pknot` structure. The actual drawing is initiated by a line in the `.r2r_meta` file, and running extra commands to create the pseudoknot view of the RNA structure, as in Section 3.7.

It is also possible to draw callout-style pseudoknotted structures without the use of the modular structure logic, but instead using the `define` and `ifdef` commands to draw the main RNA and separately draw the pseudoknot. The associated files `demo/demo-pknot-callout-ifdef.sto` and `demo/demo-pknot-callout-ifdef.r2r_meta` demonstrate how to do this.

Once R2R has drawn the pseudoknot and the main structure, these need to be assembled in a drawing program like Adobe Illustrator or Inkscape. One style of this assembly is shown in Figure 3.16C.

Note: R2R will attempt to automatically join the lines or arcs that comprise an outline corresponding to a pseudoknot. However, sometimes its heuristics will fail, and it might be missing some lines, i.e., some nucleotides will not be outlined. In this case, try disabling the automatic joining behavior:

```
#=GF SetDrawingParam outlineAutoJoin false
```

Figure 3.16 A pseudoknotted RNA drawn in callout style

A

```
# STOCKHOLM 1.0
rope                        AGGCAUUUGAACCAUAUUGUGCGCCUAACAUC..GCCAAAGCACAA
speed                       GGGUAUUUGAACUGUAUUAUGCACCCAGCAUAAUGUGGAACCAUAA
topology                    AGGUAUUUGAACCGUAUUGUGCACCUAGCAUGA.GUUAAAGCACAA
#=GC SS_cons                <<<<..................>>>>....................
#=GC SS_cons_1              ..................<<<<..................>>>>..
#=GC R2R_LABEL              ...............................1.2...3........
#=GC SUBFAM_pknot_R2R_LABEL ------------------....------------------....--

#=GF R2R ignore_ss_except_for_pairs _1 outline
#=GF R2R var_backbone_range 1 2
#=GF R2R place_explicit 3 3-- -45 1 0 0 0 -90

#=GF SUBFAM_PERL_PRED pknot return 1;
#=GF SUBFAM_pknot_R2R subst_ss _1 primary
#=GF SUBFAM_pknot_R2R no5
#=GF SUBFAM_pknot_R2R outline_nuc all
#=GF SUBFAM_pknot_R2R set_dir pos0 90 f
//
```

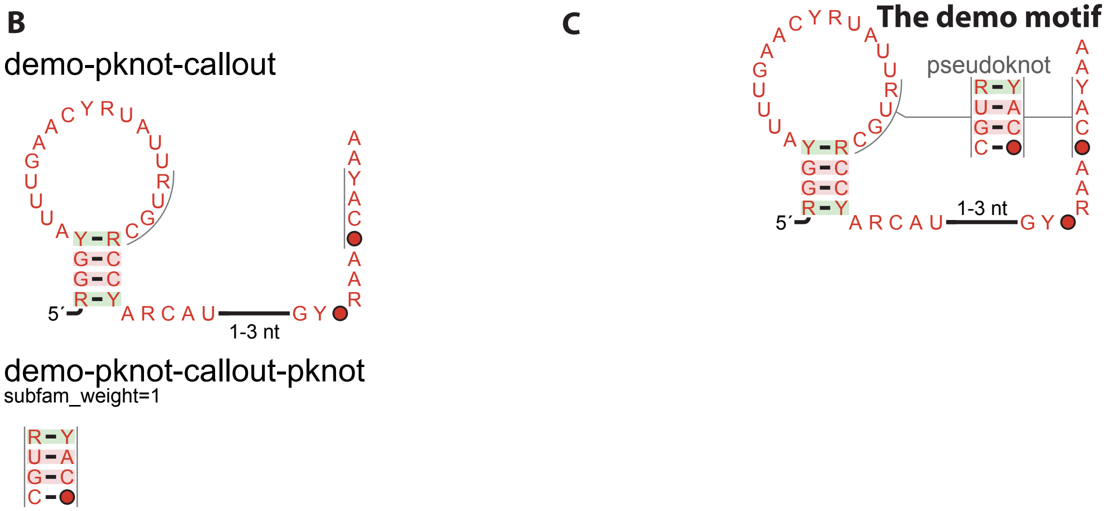

(A) The Stockholm file `demo/demo-pknot-callout.sto`. The structure of this hypothetical RNA is inspired by that of SAM-V riboswitches [8], but is simplified for this example. (B) The raw output of R2R when run on `demo/demo-pknot-callout.r2r_meta` / `demo/demo-pknot-callout.sto`. (C) Assembled drawing. To create this drawing, the components of part B were combined using Adobe Illustrator.

This case arises with the first pseudoknot in SAM-IV riboswitches [16], for which I disabled automatic joining (see `demo/SAM-IV/SAM-IV.sto`)

3.8.3 Pseudoknots drawn with in-line style

Sometimes the geometry of a particular RNA motif allows the pseudoknot base pairs to be drawn without the use of a callout. In these cases, it is often desirable to use an "in-line" drawing of the pseudoknots, as shown in Figure 3.17. However, even if you want to draw an in-line style, I recommend starting with a basic callout-style drawing, because it's easier to understand the RNA and decide on a final layout.

Internal loops, bulges, terminal loops and multistem junctions are by default drawn along a circle. However, adding `place_explicit` commands that refer to positions within these loop regions will cause them to be drawn in a straight line. In cases where you want a part of the original loop to be drawn along a circle, you can use the `bulge` or `bulge_flip` commands.

In order to achieve an in-line pseudoknot layout, it is often necessary to flip stems, using the `f` option to the `place_explicit` command. Sometimes it can be confusing to get the proper stems flipped. It is useful to note that R2R has default rules for layout. When you use a `place_explicit` command, your command will override default rules. (Specifically it will override any *conflicting* default rules.) To diagnose problems with flipped stems, try using the `mark_flip` R2R command, which shows you in the drawing whether each element is flipped or not. Another strategy is to simply remove the `f` marking from `place_explicit` commands that you might think should be flipped, and see if that helps. Finally, you can use the command `SetDrawingParam showPlaceExplicit true` to show you how your `place_explicit` commands were used in the positioning, and where default rules were used (which you might want to override with additional `place_explicit` commands). The `showPlaceExplicit` option is demonstrated in Figure 3.17.

Examples of R2R-based RNA drawings with in-line pseudoknots are as follows. Most of these use flipped stems.

- SAH [12, 13] (see `demo/SAH/SAH.sto`)
- PreQ$_1$-II [13] (see `demo/22/preQ1-II.sto`)
- The *pfl* motif [17] (see `demo/104/pfl.sto`)
- The *ykkC*-III motif [17] (see `demo/104/ykkC-III.sto`)
- The Downstream-peptide motif [17] (see `demo/104/Downstream-peptide.sto`)

.

3.9 Preparing presentations using projectors

This section concerns the default colors for shading base pairs, which indicate covarying mutations. These colors were selected with journal papers in mind. The colors are clear in print or on a computer monitor, yet are not too distracting. However, these colors are too light for many projectors. Therefore, if you're doing a talk and want the audience to be able to see the shading, I recommend darker colors.

To modify the colors in an R2R drawing, you can add the following R2R command:

```
SetDrawingParam pairShadingColors rgb:156,199,153 rgb:152,199,222 rgb:235,138,126
```

Figure 3.17 A pseudoknotted RNA drawn in inline style

A

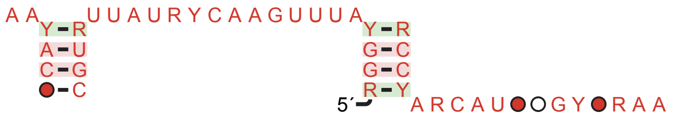

B

```
# STOCKHOLM 1.0
rope                        AGGCAUUUGAACCAUAUUGUGCGCCUAACAUC..GCCAAAGCACAA
speed                       GGGUAUUUGAACUGUAUUAUGCACCCAGCAUAAUGUGGAACCAUAA
topology                    AGGUAUUUGAACCGUAUUGUGCACCUAGCAUGA.GUUAAAGCACAA
#=GC SS_cons                <<<<..................>>>>....................
#=GC SS_cons_1              ..................<<<<..................>>>>..
#=GC R2R_LABEL              ....a.................t...b....1.2..........3.

#=GF R2R place_explicit t-- t 0 -1 0 0 0 0 f
#=GF R2R place_explicit 3 3-- +45 1 0 0 0 +90 f
#=GF R2R var_backbone_range 1 2
#=GF R2R bulge a
#=GF R2R bulge_flip b
//
```

C **D** **E**

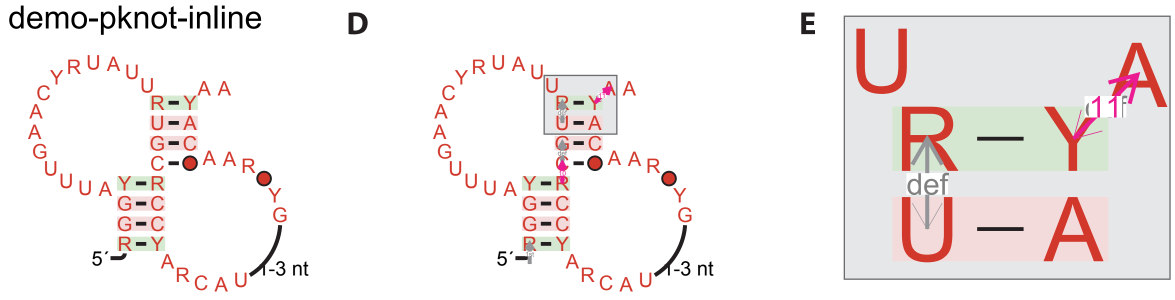

The same RNA structure depicted in Figure 3.16 is now drawn with an in-line-style pseudoknot. (A) R2R's default drawing of the structure. R2R does not attempt to make a feasible in-line-style drawing of a pseudoknot structure, since this is quite difficult, and sometimes not possible within R2R's quality constraints. (B) Stockholm input file. This is the contents of `demo/demo-pknot-inline.sto`. (Some commands were removed, but these lines are not necessary to generate the drawing in part C.) (C) Raw output of R2R when run on the Stockholm file in part B. (D) Output of R2R when run using the command `R2R SetDrawingParam showPlaceExplicit true`, which marks where `place_explicit` commands were used. The boxed region is shown at a larger scale in the next subfigure. (E) Expansion of the region boxed in part D. The pink arrow labeled '11' on the top left corner refers to a `place_explicit` command on line 11 of the input `.sto` file (`demo/demo-pknot-inline.sto`). The pink line has two arrowheads. The thicker one (pointing up and right) indicates the direction in which layout actually proceeded, although it is not usually necessary to know this. The gray arrow on the left labeled 'def' shows a default rule that R2R used to position these nucleotides. In this case, the default rule says that a helix should be positioned in a linear arrangement. These arrows are sometimes useful to diagnose problems with `place_explicit` commands. The arrows and their labels are intentionally small so that they clearly indicate the relevant nucleotides in an automated drawing. Most PDF and SVG viewers allow zooming in, to expand the scale of these features.

(thanks to Kirsten F. Block for these RGB color values).

Or you can change the colors of an existing drawing in a drawing program. For example, Adobe Illustrator allows you to select all object that have the same fill color as a selected object. So, you can select one covarying (pale green) base pair, and then get all the others, to change this color.

3.10 How to draw GOLLD RNA from R2R output

As stated previously the published drawing of GOLLD RNA [15] was based on the most commonly observed structural elements. Creating the drawing based on the output of R2R therefore requires some assembly. The drawing is generated from the demo files `demo/exceptional/GOLLD.sto` and `demo/exceptional/GOLLD.r2r_meta`, and this description refers to the PDF or SVG output generated when R2R is run on those files. Most elements of the drawing come from the default drawing, called "GOLLD". However, the second multistem junction (numbered from the $5'$ end) is taken from the "GOLLD-skipbadd2" drawing. The third and fourth domains are taken from the "GOLLD-d3classic" drawing. All other elements of the structure come from the default drawing.

3.11 Making the demo output files

To create the demos yourself, `cd` into the `demo` subdirectory. To create the PDF output in the `output-pdf` directory, run `make all-pdf`. Similarly, to create SVG, run `make all-svg`. I recommend that you *not* run plain `make` or `make all`, because of unlikely but possible conflicts with the `.solver-cache` file, as the same file is used both for PDF and for SVG generation. You might need to delete the files in `output-pdf` or `output-svg` in order to force the `make` command to re-build them.

If you wish to really make the files from scratch, also delete the files in the `intermediate` subdirectory and `.solver-cache` files in various subdirectories, to force CFSQP to run. (Of course, this requires that you have CFSQP.) Note that this process will take a considerable amount of time (possibly hours), because several RNAs use the solver, and the solver sometimes requires significant amounts of time to obtain optimal solutions. (Tip: if you have GNU make, and have a multiprocessor machine, you can use the `j` flag. For example, if you have 4 CPUs and want to make PDF files, run `make -j 4 all-pdf`. You can determine if you have GNU make by running `make --version`; GNU make will say "`GNU Make`" on the first line.)

Chapter 4

Reference: automated inference of nucleotide conservation levels

In order to draw a consensus diagram, it is necessary to determine which nucleotides are conserved (i.e., are part of the consensus sequence). It is also useful to determine how conserved they are, and to annotate which base pairs exhibit covariation. R2R can perform this calculation, and this output is subsequently used by R2R to actually draw this consensus information. This process of inferring a consensus, then drawing it, was illustrated in Chapter 3. To draw a single RNA molecule, it is not necessary to determine a consensus.

The definition of these consensus statistics was defined in a previous report[13], which read

> "To establish the extent of conservation reflected in consensus diagrams..., sequences were weighted to de-emphasize highly similar homologs. Weighting used the GSC algorithm[3], as implemented by Infernal[2], and weighted nucleotide frequencies were then calculated at each position in the multiple sequence alignment. To classify base pairs as covarying, the weighted frequency of Watson-Crick or G-U pairs was calculated. However, aligned sequences in which both nucleotides were missing or where the identity of either nucleotide was uncertain (e.g. was 'N', signifying any of the four bases) were discarded. Classification as a covarying position was made if two sequences had Watson-Crick or G-U pairs that differ at both positions amongst sequences that carry the motif. If only one position differed, the occurrence was classified as a compatible mutation. However, if the frequency of non-Watson-Crick or G-U pairs was more than 5%, we did not annotate these positions as covarying or as compatible mutations."

Note: it is now possible to weight sequences using methods other than the GSC algorithm (see below).

4.1 Recommended command

The standard command line is:

`r2r [flags] --GSC-weighted-consensus` *input-sto output-sto* `3 0.97 0.9 0.75 4 0.97 0.9 0.75 0.5 0.1`

where *input-sto* is the name of a Stockholm-format file containing your alignment. This command infers a consensus of 3 degrees of nucleotide identity, with weighted conservation thresholds of 97%, 90% and 75%; 4 levels of presence conservation of nucleotides whose identity is not highly conserved,

with thresholds 97%, 90%, 75% and 50%; and tolerance for up to 10% of sequences having non-canonical base pairs in a given paired position. The command will create a file *output-sto* that will have the same content as *input-sto*, but will contain additional markup summarizing what is conserved.

In the *output-sto* file, the line `#=GC cons` will contain a dash (`-`) for columns that are classified as gaps, and a nucleotide for non-gap columns. Gap columns are, by default, removed from the consensus when R2R draws it. See Section 4.5 for details on the added contents of the *output-sto* file.

Available flags that alter the behavior of this process are described in Section 4.5.

4.2 "Fragmentary" alignments

The "fragmentary" feature described in this section was motivated by GOLLD RNA [15]. GOLLD RNAs average roughly 800 nucleotides in size, but are predominantly found in metagenome data from Lake Gatún that consists of short sequence scaffolds. Therefore, most of the detected GOLLD RNAs are fragments of the full RNA structure. Moreover, since relatively few GOLLD RNAs are currently available, it is not practical to discard fragmentary GOLLD RNAs, as this would greatly reduce our ability to analyze its structure.

To handle this situation, R2R has a "fragmentary" mode. In this mode, for example, any sequence that consists entirely of gap characters on its $5'$ end is assumed to be a fragment that is cut off at this $5'$ end. In this case, nucleotides that are expected to be $5'$ to this cut-off point are treated as unavailable data. As such, they do not participate in any of the conservation statistics calculations—neither in the degree of conservation of nucleotides, nor in calculation of base pair covariation. Similar semantics apply to $3'$ ends that consist entirely of gap characters. The fragmentary mode was also applied to HEARO RNAs [15] in the case of the $3'$-most hairpin that is often missing, presumably due to truncation events.

Fragmentary mode is enabled by adding the following line to a `.sto` file:

```
#=GF FRAGMENTARY 1
```

4.3 Weighting sequences using your own algorithms (for calculating nucleotide frequencies)

WARNING: please be careful about using this functionality. It is not appropriate in the vast majority of cases. In almost all scenarios, it is most correct to use R2R's default weighting.

4.3.1 Why R2R weights sequences

Nucleotide frequencies have often been calculated by simply counting the number of sequences with a particular nucleotide in a given alignment column, and dividing that by the total number of sequences. For example, suppose there are 10 sequences in an alignment, and 9 of them have an A nucleotide in some particular column, while the 10th sequence has a C in that column. The just-described approach would report that the A nucleotide is then 90% (9/10) conserved.

However, biological sequences are typically correlated through evolution, and are therefore not indpendent samples. For example, suppose that the 9 sequences with an A nucleotide are all various types of primates, and the 10th sequence is a kangaroo. In this case, the 9 primate sequences are

essentially redundant. In other words, the fact that the A nucleotide occurs in 90% of cases says much more about what organisms people decide to sequence, and not very much about evolutionary constraints on this nucleotide position. A better estimate would be that the A nucleotide is actually only 50% conserved.

This issue can also occur even within metagenomic sequences from environmental samples (e.g., bacteria in the human gut), even though no-one chooses the organisms in an environment. First, there is a bias in which environments are chosen to be sequenced (e.g., there is vastly more metagenomic sequence from the human gut than from the guts of any other animal). More importantly, the frequency of a particular RNA sequence is not likely to be proportional to the fitness or other property of the RNA. Rather, the frequency of a given RNA sequence is often more related to the fitness of the organism in which it resides—even a pseudogene can be highly abundant in nature if it happens to be encoded within a prolific organism.

Therefore, R2R weights sequences to try to correct for these issues, and does this with the GSC algorithm[3] (as implemented by the Infernal[2] software package the R2R uses).

The one case in which I have used my own sequence weighting is for a project where sequence pools from *in vitro* selection were sequenced using high-throughput sequencing. In this case, it's reasonable to assume that the fitness of a sequence is proportional to its frequency, or at least is the dominant factor. (This assumption ignores evolutionary correlation between the sequences, so it's not perfect.)

You might also use this functionality if you know of a better weighting algorithm, i.e., one that solves the redundancy problem better than the GSC algorithm.

4.3.2 How to use your own weights

If you really need to weight sequences yourself, you must create a version of your Stockholm alignment that has a line specifying the weights, as follows:

`#=GF USE_THIS_WEIGHT_MAP` *map*

where *map* is a space-separated list, where each element of the list specifies a hitId identifying a sequence in the alignement, followed by a space, followed by the weight of that sequence. For example, the following is a valid Stockholm file with custom weights:

```
# STOCKHOLM 1.0
A            CCAAAGG
B            ACUAUGU
#=GC SS_cons <<...>>
#=GF USE_THIS_WEIGHT_MAP A 5 B 1
//
```

and says that RNA molecule `A` should be weighted 5 times more than molecule `B`.

Then run the --GSC-weighted-consensus command on this augmented Stockholm file. The --GSC-weighted-consensus command will use the specified weights whenever the `#=GF USE_THIS_WEIGHT_MAP` tag is present.

4.4 General command

This section gives additional detail on the command that will likely not be relevant to most readers' needs.

`r2r` *[other flags]* `--GSC-weighted-consensus` *input-sto output-sto identity-levels* [...] *present-levels* [...] *max-non-canon*

where

- optional *[other flags]* are:
  - `--maxNonCanonInNoVariationObserved` *fraction* : by default *fraction*=1. This means that R2R will tolerate up to 10% non-canonical base pairs, while depicting base pairs that do not vary (among canonical pairs). A fraction of *fraction*=0 will prevent non-canonical base pairs, and is probably a better idea.
  - `--cutEmptyLines` : in some circumstances, `r2r` must eliminate lines from the input file. This occurs when the input file has lines such as `#=GC cons` that `r2r` wants to output. Instead of deleting the lines, `r2r` will substitute these lines with empty lines, in order to keep the line numbers consistent, so that any error messages will make sense with the line numbers of the original file. You can suppress these empty lines with the `--cutEmptyLines` flag.
  - `--topNMostConserved` *N* : indicate which of the columns are among the *N* most conserved. The output is in the form of an extra label line "`#=GC R2R_XLABEL_mostcommon`". The conserved columns are indicated with an '`x`', while the other columns have a dot.
  - `--position-based-weights` : do not weight sequences with the GSC Algorithm, but instead use a position-weight-based algorithm (which is also implemented by the Squid library of the older version of Infernal that is included as part of the R2R code). This algorithm is significantly faster with many sequences, but might not work as well as the GSC algorithm.
  - `--verbose` : print out a bit of extra info to `stderr`. The extra information is very limited, so "verbose" is kind of an exaggeration.
- *identity-levels* is an integer *N* followed by *N* real numbers. *N* defines the number of different levels for classifying the degree of conservation of a nucleotide identity. The real numbers define a minimum frequency (from 0 to 1) for each level, and must be in decreasing order.
- *present-levels* is also an integer *N* followed by *N* real numbers. In this case, *N* defines the number of different levels for classifying how frequently the nucleotide is present. The real numbers define minimum frequencies (0 to 1) for each level, and must be in decreasing order.
- *max-non-canon* is a real number specifying the maximum frequency (0 to 1) of non-canonical base pairs. Non-canonical base pairs are nucleotide pairs that are not A-U, C-G, G-C, U-A, G-U or U-G. If the number of non-canonical base pairs in paired-columns exceeds *max-non-canon*, then R2R will never declare covarying, compatible, or non-mutating pairs. In other words, it will always declare code `?`, which corresponds to base pairs that are not shaded in consensus diagrams.

A concrete example of these parameters and their meaning was given in Section 4.1.

Note: if *identity-levels* is greater than 3 or *present-levels* is greater than 4, R2R is not designed to be able to draw the consensus diagram, although it would be relatively straightforward to extend it to interpret additional levels. I have assumed this is not likely to be an important feature.

4.5 Output of R2R for the consensus

You probably won't have to understand R2R's consensus output, except to understand that R2R removes gap columns when it draws the consensus (though you can override this with the `keep` command), and that it annotates these gap columns in the line beginning `#=GC cons`.

R2R's consensus output consists of a series of `#=GC` lines that annotate per-column data. These lines are:

- `#=GC cons` : Consensus sequence. Gaps are represented as dashes (`-`). Nucleotide symbols (`A`, `C`, `G`, `U`) represent conserved nucleotides. Two degenerate IUPAC symbols are used: `R` means the position is conserved as either A or G, and `Y` represents either C or U. A lower case '`n`' is used for columns that are typically or always present, but do not conserve the nucleotide identity. These positions are drawn as circles by R2R. An annotated example is given in Figure 3.1D.

- `#=GC conss` : The degree of conservation. "`1`" is the highest level of conservation. When the consensus letter (in `#=GC cons`) is a nucleotide or degenerate nucleotide, by default the levels range from `1` to `3`, and represent how often that nucleotide is found in this alignment position. When the consensus letter is lower-case `n`, by default the levels range from `1` to `4`, and represent how often any nucleotide is found in this position (as opposed to a gap). When the consensus letter is a dash (representing a gap), the number is always `0` (zero). An annotated example is given in Figure 3.1D.

- `#=GC cov_SS_cons` : Covariation annotation. `2` represents base pairs that show at least one instance of covariation, `1` means there is at least one compatible mutation, `0` (zero) means there are no mutations, so no data. `?` means that there are too many non-canonical base pairs. An annotated example is given in Figure 3.1D.

- `#=GC cov_SS_cons`*x* : covariation data for pseudoknot structure line `#=GC SS_cons`*x*.

- `#=GF NUM_COV` : Number of base pairs with covariation. (per-file annotation)

- `#=GF WEIGHT_MAP` : Lists GSC-algorithm weights used for each sequence, in a space-speareted list. The even-numbered fields identify a sequence, and the odd numbered fields are the weights. (per-file annotation) Note: if you use `#=GF USE_THIS_WEIGHT_MAP`, the results of `#=GF WEIGHT_MAP` will be identical to the weights you supplied

- `#=GF USED_GSC TRUE` : Confirms that R2R used the GSC algorithm. (per-file annotation)

- `#=GC col_entropy_`*i* : Experimental feature, the entropy of each column, written vertically.

- `#=GF DID_FRAGMENTARY FALSE` : Use of `FRAGMENTARY` feature is recorded. (per-file annotation)

4.6 Generating your own alignment consensus, bypassing R2R

When drawing a consensus, R2R depends on data added by the `--GSC-weighted-consensus` command, which is added to the original information in the Stockholm-format alignment file. An alternate program to calculate the consensus data need only output these extra data fields. The required fields are `#=GC cons`, `#=GC conss`, `#=GC cov_SS_cons` and for any extra stem `#=GC SS_cons`*i*, there

must be `#=GC cov_SS_cons`x. The form of these fields is described in the previous section. Examples can be generated from the `demo` files, by looking at the output of the `--GSC-weighted-consensus` command in the `demo/intermediate` directory (the file names will end with `.cons.sto`).

R2R is, however, committed to up to 9 conservation levels, each corresponding to a different color. For a larger set of colors, the color of nucleotides can be overridden later using the `nuc_color` command (see Section 5.8.6.4). There is no command to specify an arbitrary color for shading of base pairs.

Chapter 5

Reference: drawing

5.1 Running R2R

R2R processes Stockholm-format (`.sto`) files with special markup. Note that interleaved lines are not permitted, so technically R2R can only process Pfam format. R2R has some other incompatibilities with Stockholm format, as explained in section 5.2.2 for details. You can convert Stockholm-format alignments into the Pfam format using the `esl-reformat` command that comes with the HMMER and Infernal software packages in the `easel/miniapps` directory.

The files must have information on the consensus, which is also made by running R2R (see Chapter 4).

There are two command lines you can use to process Stockholm-format alignments:

1. `r2r` *input-file*`.sto` *output-file*

   where *output-file* will be created in either Adobe Acrobat PDF format or Scalable Vector Graphics (SVG) format (see below), and *input-file*`.sto` is a file in Stockholm format.

   Note: some functionality is not available in this command-line format, so option #2 below is preferable.

2. `r2r` *input-file*`.r2r_meta` *output-file*

   where *output-file* will be created in PDF or SVG format, and *input-file*`.r2r_meta` is a file that lists individual Stockholm-format files, and has additional options. Its format is described in Section 5.5. The diagrams drawn will be organized into a grid in *output-file*.

The *output-file* name must end in either `.pdf` or `.svg`, which determines what output format R2R will write. Note: PDF output is optimized for Adobe Illustrator, while SVG output is optimized for Inkscape, mainly in terms of the font. Of course, with SVG output, you can easily do a search & replace to change the font. However, R2R internally knows (approximately) font metrics, which allows it to position text (almost) accurately. Therefore, you should use a font that is related to Helvetica: Helvetica, Arial, Bitstream Vera Sans or DejaVu Sans.

The following flag can be added after `r2r`:

- `--disable-usage-warning` : do not output the warning at the top of the PDF or SVG file. By default, R2R warns about appropriate usage.

- `--inkscape-helvetica-font-name` *fontName* : use this font name in output SVG files. Inkscape is sometimes happier if this name matches a font that it has. The default is "`Bitstream Vera Sans`".

5.2 About the Stockholm file format, and how R2R uses it

R2R uses the generalizable format of Stockholm files to specify markup that defines how a motif is drawn. Stockholm format is explained on Wikipedia (http://en.wikipedia.org/wiki/Stockholm_format).

R2R's deviations from the Stockholm format are explained in section 5.2.2.

The key ideas is that R2R uses the following types of annotation:

- `#=GF` *tag data*

  This specifies file-level markup. For example R2R drawing commands use the form (*tag* = `R2R`):

  `#=GF R2R` *command*

- `#=GC` *tag columns*

  In this case, each position in the string *columns* corresponds to each column in the alignment. For example, for *tag* = `SS_cons`, the symbols in the *columns* string specify the consensus secondary structure of the alignment.

- `#=GR` *hitId tag columns*

  Each position in the string *columns* corresponds to each column in the sequence specified by *hitId* (see Section 5.4.1). This is relevant to identifying columns in an individual RNA when the user wants to draw that RNA sequence, instead of the consensus.

Pedantic point: R2R's input format does not quite respect the Stockholm format, or even the more restrictive Pfam format. For example, it uses many `#=GF R2R` lines, each of which represents a distinct command. However, the Stockholm format says that these lines are logically part of the same line, and the separation of commands is technically not enforced. However, has not been a problem in practice.

5.2.1 A note on R2R's representation of consensus secondary structures

R2R uses a non-standard method for writing consensus secondary structures for motifs with pseudoknots. To describe pseudoknots, in addition to the standard `#=GC SS_cons` line, there can be other lines of the form `#=GC SS_cons`*X* for any string *X* (typically, I call them `#=GC SS_cons_1`, `#=GC SS_cons_2`, ...).

To mark base pairs, a structure line must contain only left brackets (“(”, “[”, “<” or “{”) or right brackets (“)”, “]”, “>” or “}”). All other symbols are assumed to be single-stranded. (Thus, R2R does not use Rfam's method for marking pseudoknots.)

I find that this method reduces special-case code because all pairs are specified in the same way. It also allows for nucleotides that can be in more than one base pair interaction (not necessarily simultaneously). This is occassionally relevant to conserved structures (e.g., with alternate pairings), and is convenient when the exact structure is unknown.

R2R does not work with consensus structures in Rfam format. Supporting such a format automatically would require some rethinking of structure-related commands such as `ignore_ss_except_for_pairs`.

5.2.2 R2R does not conform to the Stockholm file format

R2R is not fully compatible with the Stockholm file format. Thanks to Elena Rivas for helping with this information.

- R2R cannot process interleaved lines, and thus it truly only processes "Pfam format", which is a subset of Stockholm format. With interleaved lines, the sequence for a given "hit" within an alignment can span multiple lines of text, and this is used to reduce the width of files. In Pfam format, sequences and column annotation (for #=GC and #=GR lines) must be written in one line.

  You can convert Stockholm-format alignments into the Pfam format using the `esl-reformat` command that comes with both the HMMER and Infernal software packages in the `easel/miniapps` directory (in both software packages).

- R2R and the Stockholm format differ in their usage of whitespace, which is defined as one or more consecutive non-printable characters, like spaces, tabs or newlines. R2R requires that most whitespace consist of exactly one space or tab character. Whitespace separates components of the Stockholm format. For example, the line

  ```
  #=GC MAGIC_COLUMNS ......*......**
  ```

  has the components `#=GC`, `MAGIC_COLUMNS` and `......*......**`. Each of these components are separated by whitespace. For R2R, there must be exactly one space or tab character between `#=GC` and `MAGIC_COLUMNS`, but the Stockholm format allows multiple spaces. Some programs use multiple spaces to line things up to make it easier for humans to read, or for other reasons. Such alignments would need to have the extra spaces removed in order for R2R to be able to parse them. Multiple spaces are allowed by R2R between `MAGIC_COLUMNS` and `......*......**`.

- As noted in the previous section, R2R's definition of conserved secondary structure differs from the mechanism used by the Rfam Database. However, this definition does not belong to the Stockholm format definition.

5.3 R2R "drawing units"

R2R divides an RNA structure into units, and positions each unit as a block. Units are: (1) a single stranded region, including terminal loops, (2) both sides of an internal loop or (3) a stem. Some commands operating on labels will have no effect if the label corresponds to a position in the middle of a unit. For example, to use the `turn_stem_at_internal` command, you must specify the $5'$-most nucleotide in the internal loop (although in this command, the $3'$-most nucleotide is also permitted).

`place_explicit` commands that reference the midding of a drawing unit will break it into two units.

5.4 Data types in R2R

5.4.1 hitId

I define hitIds as strings in Stockholm-format files that identify each sequence. In Stockholm files, the hitId begins a line, and is followed by whitespace, which are then followed by the RNA sequence with gap characters.

5.4.2 Distances

Distances within drawing commands are specified in internucleotide units. One internucleotide unit is the distance between two consecutive nucleotides along the backbone, and defaults to 0.105 inches.

5.4.3 Angles

Angles are always specified in degrees.

5.4.4 Measurements (length/width/size)

Some constant lengths used by R2R can be set to non-default values using the `SetDrawingParam` command (see Section 5.5.3). The default units are inches, except for font sizes. Font sizes are by default measured in points. Suffixes can be used to specify other measurement units. No whitespace is allowed between the number and the suffix. The following measurements are equivalent:

- `1` (default is inch)
- `1in` (just makes the default explicit)
- `2.54cm`
- `25.4mm`
- `72pt`

5.4.5 Colors

Colors (e.g. in the `circle_nuc` command) are specified as follows.

- RGB colors are written in the form

  `rgb:`*r*`,`*g*`,`*b*

  where *r,g,b* (written separated by commas) are numbers from 0 to 255.9999.

- Standard colors used in cleavage diagrams for in-line probing experiments can also be used. They are specified as `cleavage:?` (unknown/no data, gray), `cleavage:=` (constitutive, yellow), `cleavage:-` (decreasing, red), `cleavage:+` (increasing, green).

5.5 .r2r_meta file

You specify what files R2R should process in a file with extension `.r2r_meta`. The file is tab delimited—all fields must be separated by tab characters. If the first (tab-delimited) field is empty, the line is ignored (i.e., this is a comment).

If a line has only one field, that field is the path to a Stockholm-format alignment file. This file is processed to draw a consensus diagram.

If the line's first field is `SetDrawingParam`, then you can change the default settings of certain drawing parameters (see below).

You can also specify that a single RNA molecule should be drawn (rather than a consensus), as described below.

5.5.1 Display name

R2R will output multiple drawings into its output PDF or SVG file. There is one drawing per line in the `.r2r_meta` file. By default, R2R creates names for these drawings that are based on the file name and any other parameters you specify (such as 'defines' or 'oneseq' mode, as explained in the following subsections). You can override the name for any line (in the `.r2r_meta` file) by adding

`displayname` *name*

to the line (as tab-delimited fields, like normal).

This functionality is for R-scape, and any other software that automatically generates R2R input.

5.5.2 Defines

You can define symbols that control which R2R commands are processed, as described in Section 5.8.1. You can add `define` *name value* to any line that specifies a `.sto` files in the `.r2r_meta` file. These defines affect the drawing of that `.sto` file.

For example, see the file `demo/SAM-IV/SAM-IV.r2r_meta`. This file directs the drawing of SAM-IV riboswitches in various ways, and was used for the various examples in the tutorial that used SAM-IV.

Defines are only applicable to the line in which they appear.

5.5.3 SetDrawingParam

You can include lines like the following in the `.r2r_meta` file

`SetDrawingParam` *name value*

to change internal drawing parameters in R2R. This affects all subsequent lines of the `.r2r_meta` file.

Valid *name* codes are:

- Miscellaneous font sizes (note: as with all font sizes, default units are points for font sizes only)
  - `nameFontSize` : for text that shows the name of RNA motifs. (Default: `12pt`.)

- nucFontSize : writing nucleotides (Default: 7.5pt.)

- Nucleotide layout distances
  - internucleotideLen : distance between consecutive nucleotides along the backbone. (Default: 0.105in.)
  - pairLinkDist : distance between base-paired nucleotides. (Default: 0.17in.)

- tick_label nucleotide labels
  - nucTickLabel_distFromNuc : the distance from the nucleotide at which the tick line starts. (Default: 3.75pt.)
  - nucTickLabel_tickLen : the length of the tick line. (Default: 4pt.)
  - nucTickLabel_tickColor : the color of the tick line. (Default: rgb:0,0,0 , i.e., black.)
  - nucTickLabel_tickPenWidth : the width of the tick line. (Default: 0.5pt.)
  - nucTickLabel_extraSpaceToText : extra distance between the end of the tick line and the start of text (might be a bit off because R2R still does not use absolutely exact font metrics). (Default: 1.5pt.)
  - nucTickLabel_fontSize : the font size used to draw the label. (Default: 6pt.)

- backboneWidth : width of lines that represent the backbone (used for skeleton drawings and for variable-length regions). (Default: 1.5pt.)

- outlining/inlining nucleotides (also used to indicate nucleotides involved in pseudoknot pairings)
  - outlineNucExtraRadius : extra distance from outside of nucleotide that is added before the outline line. (Default: 3.75pt.)
  - outlineNucPenWidth : width of pen used to draw the outline. (Default: 0.5pt.)
  - outlineNucColor : color used. (Default: rgb:92,92,92.)
  - circleRadiusToSmoothDirectionChange : when two straight lines in the outline meet, the direction change is smoothed using a circular arc. This parameter is the radius of the arc. (Default: 0.025in.)
  - outlineAutoJoin : if true, R2R will attempt to join lines and arcs that are part of the outline, for both outlines generated implicitly by pseudoknots and by outlines generated explicitly with the commands outline_nuc or inline_nuc. Sometimes R2R's heuristics for drawing outlines fail, and so it is better to doing the joining yourself. (Default: true.)
  - prefixSsWithPkInDrawings : When you configure an SS_cons line to be a callout-style pseudoknot by using the ignore-ss-except-for-pairs command, R2R will indicate the two sides of each stem with the text pk_... For example, with SS_cons_1, the added text would be "pk_1". This behavior corresponds to prefixSsWithPkInDrawings being true. If prefixSsWithPkInDrawings is set to false, R2R will not use the prefix pk_, but will just use the label past SS_cons_, so in our example the label would be "1". (Default: true.)

- Circling nucleotide (also used for cleavage diagrams in oneseq mode)

- cleavageIndicatorRadius : radius of the circle. Note: R2R will refuse to allow this to be greater than half the length of internucleotideLen, since then circles would overlap. (Default: 3.75pt.)
- cleavageIndicatorPenWidth : width of line surrounding the circle. (Default: 0.5pt.)

• Drawing base pairs bonds

  - pairBondLen : the length of the line connecting Watson-Crick base pairs. (Default: 0.054in.)
  - pairBondWidth : the width of the line connecting Watson-Crick base pairs. (Default: 0.02in.)
  - pairBondGURadius : radius of the filled circle for representing G-U wobble pairs (only used in oneseq mode). (Default: 0.02in.)
  - pairBondNonCanonRadius : radius of the un-filled circle for representing non-canonical base pairs (only used in oneseq mode). (Default: 0.01in.)
  - pairBondCircleLineWidth : width of the line of the circle used for G-U or non-canonical base pairs. (Default: 0.002in.)
  - minPairShadeGap : minimum distance between shading rectangles of consecutive base pairs. When base pairs are drawn at an angle (45 degrees is worst), the shading box's width needs to grow in order to cover the whole nucleotide letter, since nucleotides are rectangles that are always drawn in the same orientation. Eventually these shading rectangles can get very close or even overlap, which looks bad. Therefore, R2R will cheat and allow some small parts of nucleotide letters to be unshaded in order to guarantee a minimum distance between the shading rectangles, and this minimum distance is minPairShadeGap. (Default: 2.5pt.)

• Circles that represent nucleotides whose identities are not conserved:

  - anyNucCircleWidth : the width of the circle's line. (Default: 0.01in.)

• Variable-length stems, loops or linkers

  - varHairpinNumFakePairs : for the var_hairpin command, the number of fake base pairs to draw. (Integer. Default: 3.)
  - varTerminalLoopRadius : for the var_hairpin or var_term_loop commands, the radius of the arc used to represent the terminal loop. (Default: 0.17in.)
  - backboneConnectorCircleRadius :the radius of the arc that R2R uses to bend the backbone, esp. when drawing the 5′ end of the molecule. This can be more important when the 5′ end of the molecule is at a weird angle. However, because R2R sets the position of the text that says "5′" in an inflexible way, there's a very small range of values for backboneConnectorCircleRadius that will work okay. If the value is too big, R2R will draw the connecting line directly, rather than using an arc. (Default: 0.035.)
  - backboneAnnotTextOffset : the distance between the text annotation and the backbone line for var_backbone... commands. If this value is negative, then it is set as 4/7.5 the value of varBackboneFontSize. The reason for this weird ratio is that originally it was set to 4 points, and the font size was 7.5, so with this default, the default drawings will be like before (Default: -1 , i.e. set based on the ratio).

- `varBackboneFontSize` : the font size used for the annotation in the various forms of the `var_backbone...` command. (Default: `7.5pt`)
- `varTermLoopFontSize` : the font size used for the annotation in the `var_term_loop` command. (Default: `7.5pt`)

- Shading along backbone or drawing in `skeleton` mode
  - `outlineAlongBackboneWidth` : width of shading for `outline_along_backbone` command. (default: `0.7pt`.)
  - `shadeAlongBackboneWidth` : width of shading for `shade_along_backbone` command. If the value is negative, then the computer will automatically set the width based on the size of nucleotide symbols, i.e., the font size. (default: `-1`, i.e. set automatically.)
  - `alongBackboneStyle` : permissible styles are integers (default: `0`) as follows:
    * `0` : shade nucleotides with circles; join consecutive base pairs with line, but keep endpoints rounded. This tends to look good with `shade_along_backbone` where nucleotides are shaded. It is the default style.
    * `1` : shade nucleotides with an appropriately thick line that extends half-way to the previous nucleotide, and half-way to the next. This looks better for skeleton drawings where you want to change colors (otherwise lone circles look weird when they're small and not shading a nucleotide).
  - `alongBackboneMidpointGapLength` : If `alongBackboneStyle` is set to `1`, this parameter allows for gaps between the shading of consecutive nucleotides. By default differently colored consecutive nucleotides will have the two different colored shading paths joined at the midpoint between the two nucleotides. However, a gap of length `alongBackboneMidpointGapLen`[illegible] can be left between these two paths, so that they don't touch. There are two technical issues that complicate this feature. First, the length for the gap is the length along the backbone path between the two nucleotides, not the straight-line length. Thus, if the two consecutive nucleotides lie on a circle (meaning their backbone path is an arc) the straight-line gap between them will be a bit smaller than `alongBackboneMidpointGapLength`. Second, R2R draws the backbone shading as a line/arc and sets the thickness of the line to simulate the shading. The length of the gap is thus calculated at the midpoint of the shading line. But, if the path is on a circle, the two ends of the shading line will not be of equal distances. Usually the circles are of a large enough radius that these effects have no practical effect.

    (By the way, I implemented this feature to experiment with shading nucleotides involved in a pseudoknot base pair to show covariation, as an alternate way of indicating pseudoknots. These pseudoknotted nucleotides are often on a circular path. The gaps between shading are meant to simulate the gaps between consecutive nucleotides in a regular stem. Some other changes would be needed to implement this style, especially since currently all `shade_along_backbone` commands use the same drawing style, so covariation shading and other user-directed shading would conflict.)

    If `alongBackboneStyle` is not set to `1`, this parameter has no effect. (Default: `0`, i.e. don't leave any gap.)

- Debugging help

- showPlaceExplicit : Boolean (true or false). Shows information on what place_explicit commands were used to position drawing units (shown as thick lines), as well as unused commands (thin lines). The lines connect the two nucleotides that were positioned based on a place_explicit. User-specified place_explicit commands are shown in magenta, while default rules are gray. Lines are labeled. Default rules say "def". The positioning of the 5′-most nucleotide says "1st". User-specified place_explicit commands are labeled with their line number in the input Stockholm file. To simply the layout, I used a very small font size to label the lines. You will probably need to use display software to zoom into the picture in order to read the labels. (Default: false.)
- showEachNucleotideDir : Boolean (true or false). Shows the direction vector at each nucleotide. Useful in particular for working out place_explicit commands. Note that some nucleotides have two directions. These are nucleotides that are positioned in a straight line on their 5′ direction, but are on a circular layout on their 3′ end. Linear directions are shown with a normal arrow. Circular directions are shown with a line that has a small circle on the direction end. Direction vector lines always originate at the center of the nucleotide. Lines are drawn in cyan. (Default: false.)
- dumpInfoFile : if non-blank, the name of a file into which R2R should dump information on the layout and other features. See section 5.11.2. If blank, no information is output. (Default: blank.)

• Parameters relevant to skeleton mode

  - skeleton_scaleMeasurementsBy : alternate value for scaleMeasuresBy to be used in skeleton mode. (Default: 0.25.)
  - skeleton_pairBondWidth : alternate value for pairBondWidth to be used in skeleton mode. Note that this is *before* scaling is applied. (Default: 0.5pt.)
  - skeleton_backboneWidth : alternate value for backboneWidth to be used in skeleton mode. Note that this is *before* scaling is applied. (Default: 1.5pt, which is the same as the default for backboneWidth.)
  - skeleton_outlinePseudoknots : enable drawing an outline for callout-style pseudoknots. If this variable is set to true, then commands ignore_ss_except_for_pairs outline will result in an outline even in skeleton mode. If the variable is set to false, then this command will not result in an outline in skeleton mode—I think you usually don't want the outline. (Default: false.)

• Parameters relevant to circle_nuc or implicit circling from the #=GR ... CLEAVAGE line in oneseq mode.

  - nucShrinkWithCircleNuc : when the circle_nuc command is used, nucleotides are shrunk to accommodate the circles. They are scaled by a factor of nucShrinkWithCircleNuc. (Default: 0.8)
  - pairBondScaleWithCircleNuc : scaling applied to base-pair bonds to accommodate circled nucleotides. (Default: 1.)

• Parameters relevant to oneseq mode (drawing a single RNA molecule)

– drawStandardCleavage : Boolean (true or false). If true, R2R will draw the standard cleavage diagram based on the #=GR ... CLEAVAGE line. (Default: true.)
   – defaultOneseqLabeling : If true, then in oneseq mode (see below), R2R will label the number of every 10th nucleotide. (Default: true.)
   – indicateOneseqWobblesAndNonCanonicals : If true, then in oneseq mode (see below), R2R will indicate whether pairs are Watson-Crick, wobble (i.e., G-U) or non-canonical by using different bond symbols. If false, R2R will use the same symbol for all pairings, specifically all pairings will be drawn as if they are Watson-Crick. (Default: true.)
   – makeRedNucsRedInOneseq : Boolean. For any nucleotides that belong to the highest conservation level (i.e., level '1', which is usually depicted as red nucleotides that are *geq*97% conserved), color these nucleotides red in the single sequence. (Default: false.)
   – makeNonDegenRedNucsRedInOneseq : Boolean. Same as makeRedNucsRedInOneseq, except ignore highly conserved R or Y nucleotides, which in fairness aren't really that conserved. Note: if makeRedNucsRedInOneseq is true, then it doesn't matter what the value of makeNonDegenRedNucsRedInOneseq is. (Default: false.)

- pairShadingColors *covariationColor compatibleColor noMutationsObservedColor* : change the colors used to shade pairs. Colors are specified using standard R2R codes. Good colors for slides are

  ```
  SetDrawingParam pairShadingColors rgb:156,199,153 rgb:152,199,222 rgb:235,138,126
  ```

  (thanks to Kirsten F. Block for these values).

- solverMaxIters # : sets the maximum number of iterations of the solver (i.e., CFSQP) for layout of multistem junctions. Sometimes a high number of iterations is necessary since the solver gradually creeps towards the solution. However, usually fewer iterations are needed, and so the conservatively high default value is a waste of time. (Defualt: 100000.)

  Note: you can compile R2R with a new default by adding -DMAX_SOLVER_ITERS=# to the g++ command line.

- autoBreakPairs : if this variable has the value true, then R2R will automatically separate base pairs where one of them should be deleted and the other should not. This is suitable for quick drawings where you don't want to think about how to resolve these issues. If this variable has the value false, R2R will report an error. (Default: false.)

- DNA : if this variable has the value true, R2R will treat the input under the assumption that it is a single-stranded DNA molecule. In this mode, and U nucleotide in the input will be changed into a T. (Default: false.)

- disableSubfamWeightText : when you use the modular structure functionality, which is based on the SelectSubFamilyFromStockholm.pl script, this script will output a field #=GF SUBFAM_WEIGHT into the output .sto file. This weight indicates the fraction of sequences that exhibit the modular structure. r2r will then output a message at the top of the relevant drawing that states this fraction (e.g. "subfam_weight=1"). To disable this, set disableSubfamWeightText to true. If you set disableSubfamWeightText to false, R2R will print the subfam_weight if it is there. (Default: false.)

name and *value* pairs may be repeated multiple times. Thus, for example, the following line is legal, and sets 2 parameters at once:

```
SetDrawingParam  varHairpinNumFakePairs  2  varTerminalLoopRadius  0.12
```

5.5.4 Oneseq mode

If a line has three fields and the second field is equal to the literal string `oneseq`, then the first field is the path to a Stockholm alignment file, and the third field is a "hitId" (Section 5.4.1) specifying a specific sequence within the alignment. In this case, R2R will draw that sequence. It can optionally draw the sequence like a cleavage diagram for in-line probing experiments (Section 5.5.7).

5.5.5 Skeleton mode

If a line has two fields and the second field is equal to either the literal string `skeleton` or `skeleton-with-pairbonds`, then the RNA is drawn in skeleton mode. In this mode, a black line traces the backbone. Such drawings are often used to present a small sketch of an overall structure, especially for larger RNAs.

Drawing of nucleotides, nucleotide conservation, length of variable-length regions and covariation annotation are supressed. The lines that indicate base-pair bonds are supressed with `skeleton`, but drawn with `skeleton-with-pairbonds`.

Commands can be conditionally performed only in skeleton mode, or never in skeleton mode (see Section 5.8.1).

5.5.6 Entropy mode

(experimental feature)

If the line has two fields and the second field is equal to the literal string `entropy`, R2R will draw each alignment position's entropy. In this mode, columns are not removed by the `#=GC R2R_LABEL line`, or if the `#=GC cons` line says it's a gap. Similarly, `var_hairpin` and any `var_backbone`... commands are ignored. Rather, columns are only removed if there is a dash in the special `#=GC ENTROPY_DEL_COLS` line. This is to allow you to see all the columns with their entropy, and also to extend to more flanking sequence.

The resulting diagram is drawn twice: once with normal letters/colors (note: gap columns are drawn with an circle that has a gray line, rather than the usual black line.), and then with entropy. The calculated entropy in the file (by `--GSC-weighted-consensus`) is drawn. This entropy counts the gap character as a 5th nucleotide. Therefore, the entropy E is guaranteed to be in the range: $0 \leq E \leq \log_2 5$ (≈ 2.3). An entropy of zero is mapped to red, and $\log_2 5$ is blue. In between these extremes, the colors are used linearly (along the log scale).

Entropy diagrams are experimental, and the code is not guaranteed to work.

5.5.7 Cleavage diagrams

Cleavage diagrams are specific to in-line probing experiments [10], although the scheme might be applicable to other structural probing experiments. Examples of cleavage diagrams drawn using R2R are those of the SAM-IV riboswitch [16, Fig. 2(A)] and HEARO RNA [15, Supplementary Fig. 8(b)]. In `oneseq` mode (see .r2r_meta file, above), two special `#=GR` tags are processed:

- DEL_COLS : columns with a dash (-) will be deleted. These are used to specify the RNA subsequence that was actually probed. (Note that the R2R_LABEL line does not specify deletion in oneseq mode.)
- CLEAVAGE: nucleotides within a column are colored according to the following code:
  - = : constitutive cleavage (yellow)
  - - : decreased cleavage with ligand (red)
  - + : increased cleavage with ligand (green)
  - ? : no data (gray)
  - Anything else : no cleavage (not shaded)
  - (note: you can also circle nucleotides with your own colors using the circle_nuc command)
- R2R_LABEL..., R2R_XLABEL : see labels below. If present, these #=GR tags override anything in the global #=GC tags. Note: I find it easier to just use global #=GC R2R_XLABEL lines and reference them in sequence-specific drawing commands.

5.5.7.1 Multiple drawings of the same RNA molecule

In oneseq mode, you might have done multiple experiments with the same sequence, and want to show distinct data with each experiment. This situation would cause a problem because the hitId that is used with #=GR tags would have to be the same.

To avoid this problem, you can add :*construct* (where *construct* is any string that specifies a given RNA sequence in the MSA, and : is a literal colon) to the keywords DEL_COLS, CLEAVAGE, R2R_LABEL... or R2R_XLABEL... . For example,

#=GR NC_003888.3/5-50 CLEAVAGE:L

In the rest of the .sto file, and in the .r2r_meta file, this RNA would be called

NC_003888.3/5-50:L

(i.e. concatenate the hitId with the colon stuff).

Presumably the L here stands for your "Large" construct.

This scenario is illustrated by the *yjdF* motif [17]; see demo/104/yjdF.sto and demo/104/yjdF.r2r_meta. In this case, I performed in-line probing experiments for the same *yjdF* RNA homolog, but with different 3′ ends.

You could also solve this problem using defines, and draw cleavage markings explicitly with the circle_nuc command, instead of using #=GR CLEAVAGE lines.

5.6 The R2R solver cache

This section applies to the _solver commands for drawing multistem junctions. If you do not use these commands in a .sto file, this section is not relevant.

Since the _solver commands often take a long time to run, I have implemented a feature to reuse already-calculated solutions. Thus, it will be slow the first time, but will be quick in subsequent times (unless you change the parameters of the _solver command, in which case R2R must compute the solution for the altered problem). In other words, I implemented the standard "cache" strategy.

Most likely this functionality will just work and be transparent, but it is something I've implemented very recently. If you change parameters, and the solver does not re-run, try deleting the

.solver-cache file (see below for where it is). Note that even parameters not part of the _solver command can force a solver re-run. For example if you add a variablen-length region within the multistem junction or change certain drawing parameters (like internucleotideLen), this indirectly changes the problem, and the solver must solve the new problem.

Stored (cached) solutions are associated with each .r2r_meta file you use as input. If you process a file called something.r2r_meta, and there is a _solver command used in it, R2R will create a file called something.r2r_meta.solver-cache. This .solver-cache file contains cached solutions. The .solver-cache will not be created if the given .r2r_meta file does not use any _solver commands.

I have attempted to make the .solver-cache files platform independent, so that you can create a .solver-cache file on one system, and use it on a different one. However, this functionality is not well tested. In principle, you should be able to build the demo files without CFSQP because of the .solver-cache files I've provided.

5.7 Labels

"Labels" allow you to identify a column or columns in the alignment by a name. Defining column(s) by names is somewhat more convenient that using the column number, and also means that the R2R markup remains valid even if you add or remove columns. Therefore, R2R only supports referencing columns by name. (Okay, actually it supports referencing columns by number – but only do this if you're a computer.)

Tip: to see what labels are available, add an R2R command that refers to an invalid label, e.g.

```
#=GF R2R tick_label INVALID dummystring
```

R2R will give you an error message that lists the labels that are valid.

5.7.1 Main labels

The main label line is #=GC R2R_LABEL, which has 1 symbol per column. You can create multi-symbol label for each column's label with #=GC R2R_LABEL_i for integers $i = 1, 2, \ldots$ If there is a dot (.) in a column, nothing is added (i.e. the dot is the empty string). For example, the following Stockholm file has three columns with the first two columns having the labels "A" and "mul".:

```
# STOCKHOLM 1.0
x               AAU
y               CAG
z               GGC
#=GC SS_cons    <.>
#=GC R2R_LABEL  am.
#=GC R2R_LABEL_1 .u.
#=GC R2R_LABEL_2 .l.
//
```

5.7.2 Extra named label lines

Sometimes having only one set of labels (with the *R2R_LABEL* line) is not enough. You can add additional labels with names with #=GC R2R_XLABEL_*name* for some name *name*. The *name* must

not end with a number. You can create multi-symbol names by adding lines like `#=GC R2R_LABEL_`*i*, for integers *i*.

The *name* of the label is relevant when you want to refer to the label. Referencing labels is explained below in Section 5.7.5.

5.7.3 Sequence-specific (optional)

You can also have sequence-specific labels (in '`oneseq`' mode). In this case, **all** labels override anything that is specified in the global `#=GC R2R_..LABEL...` stuff. To specify sequence-specific labels:

`#=GR` *hitId* `R2R_LABEL...  ...`

Or

`#=GR` *hitId* `R2R_XLABEL...  ...`

(You can also emulate this behavior more conveniently by definining special `#=GC R2R_XLABEL_-...` label lines for each sequence.)

5.7.4 SS_cons

All `#=GC` lines beginning with `SS_cons` are added like named labels (see "extra named labels", above). In this case, for `#=GC SS_cons`, the label name is `SS_cons`. The actual string is normalized to use only the symbols '<', '>' or '`.`'.

5.7.5 Using labels and special labels

Various commands use a parameter *label*, which refers to the label of a column(s). The following are valid labels:

- `pos0` : the literal string `pos0` refers to the column #0, the 5′-most position.
- `all` : specifies all positions
- `allpairs` : specifies all positions annotated as pairing. If only `#=GC SS_cons` is used, it's equivalent to the union of columns matching label `SS_cons:<` and those matching label `SS_cons:>` . If other `SS_cons` lines are used, it applies it to them too.
- *name*`:`*label* : specifies all columns with the label *label* in the named-label *name*. (Note: the *name* and *label* are separated by a literal colon). For example, if you had a label "`e`" in the line `#=GC R2R_XLABEL_fun`, you could refer to it as `fun:e`. (Note: that was a self-describing bad joke.) The `SS_cons` lines act as named labels. Thus, `SS_cons:<` refers to all columns that are a left base pair in the `#=GC SS_cons` line.
- *label* : all columns with label *label* in the default label line (`#=GC R2R_LABEL`). This is a shortform for using the fully qualified *name*:*label* format. For example, the label "`A`" is equivalent to the label "`:A`" (note: the part before the colon is the empty string, which is the name of the `#=GC R2R_LABEL` line).
- The text "`--`" (two minus signs) can be added to the end of any label name, which gets the position immediately 5′ to the label position. Similarly, "`++`" is the position immediately 3′ to the label position. Obviously this means that labels cannot end in `--` or `++`.

- The special `notinpknot` can only be used in the context of a `SS_cons` line. For example, given `SS_cons_1:notinpknot`, the label specifies all positions that are (heuristically) assumed to not be a part of the pseudoknot that is assumed to be represented by `SS_cons_1`. That is, all positions before the first <, between the last < and the first >, and after the last >. This is useful for drawing pseudoknots using the `define` commands (and not the `SUBFAM` functionality), in the command `delcol SS_cons_1:notinpknot`.

- The special label form `#:`*col* refers to column number *col* in the input alignment. Column number zero (`#:0`) is the 5′-most (i.e. first/left-most) column.

  NOTE: In most cases, it is probably a mistake to use this label format, because changes to the alignment that involve adding or removing columns will cause the numeric reference to become wrong. However, numeric labels are useful for computer scripts that generate R2R input, where the script would simply regenerate the input upon changes to the alignment.

5.8 R2R commands

Commands are given with the text
`#=GF R2R` *commands ...*

All parameters are space-separated with *exactly* one space character. Yes, I have not implemented a robust parser.

5.8.1 Conditional commands

5.8.1.1 Define symbols

R2R implements a simple system for conditional processing that is inspired by (but much simpler than) the C preprocessor. Symbols can be defined, and R2R commands can be performed only if a given symbol is defined (or not defined) or if the symbol is equal (or not equal) to a specific value. The other conditional processing commands given in later sections are supported for backwards compatibility with previous structures I have drawn, but are interpreted in the context of defines.

The following commands operate on defined symbols:

- `define` *name value* : sets the symbol *name* to the given *value*.

- `ifdef` *name* : true if *name* has been `defined`. `ifdef` commands can apply to a single R2R command, or two multiple lines of R2R commands. If the *name* value is at the end of the line, the `ifdef` command will apply to subsequent commands, until the next `endif` command. If there is text beyond the *name* value, then the `ifdef` command will apply only to that line.

- `ifndef` *name* : opposite of `ifdef`. `ifndef` commands can apply to a single line or to multiple lines exactly like `ifdef` commands.

- `ifdefeq` *name value* : true if *name* has the value of *value*. `ifdefeq` commands can apply to a single line or to multiple lines exactly like `ifdef` commands.

- `ifdefneq` *name value* : opposite of *ifdefeq*. `ifdefneq` commands can apply to a single line or to multiple lines exactly like `ifdef` commands.

- `else` : for a multi-line `ifdef` or `ifndef`, reverses the test.

- endif : terminates a multi-line ifdef or ifndef.

The following *names* are predefined based on drawing options selected:

- oneseq : if we're in oneseq mode, then the oneseq symbol is the *hitId* that identifies the sequence. Otherwise, if we're drawing a consensus (the default), then oneseq is not defined.
- skeleton: true if we're in skeleton mode, i.e., if either skeleton or skeleton-with-pairbonds was specified in the .r2r_meta input file.
- skeleton-with-pairbonds : if skeleton was specified in the .r2r_meta command file, then this define symbol has the value false. Otherwise, if skeleton-with-pairbonds was specified, it has the value true. Otherwise, it is not defined.
- cfsqp : defined only if the CFSQP solver is available.
- entropy : defined only if entropy mode is used.

5.8.1.2 Commands that apply to only a consensus or single-molecule drawing

Commands given by

#=GF R2R_allseq *commands...*

or

#=GF R2R_consensus *commands...*

will only be interpreted if we're in drawing-the-consensus-motif mode.
Commands given by

#=GF R2R_oneseq *hitId commands...*

will only be interpreted in oneseq mode for the given *hitId*.
Setting oneseq or consensus mode is done in the .r2r_meta file (Section 5.5).
Note: these commands are equivalent to certain commands with defines, as follows:

- R2R_consensus is equivalent to ifndef oneseq
- R2R_oneseq NC_003888.3/100-200 is equivalent to ifdefeq oneseq NC_003888.3/100-200

5.8.1.3 Other kinds of conditional commands

If a command begins with any of the following words, the rest of the command is ignored in certain circumstances:

- if_skeleton : the rest of the command is processed only if skeleton drawing mode has been enabled (see Section 5.5.5). Equivalent to ifdef skeleton
- if_not_skeleton : reverse of above. Equivalent to ifndef skeleton
- if_cfsqp : the rest of the command is processed only if CFSQP is available to the program. Equivalent to ifdef cfsqp
- if_not_cfsqp : reverse of above. Equivalent to ifndef cfsqp.

5.8.2 Turning and positioning

5.8.2.1 Set_dir

The `set_dir` command is used to set the orientation of the 5′-most nucleotide. The orientation of other nucleotides are set either by R2R's default layout rules or by `place_explicit` commands.

`set_dir pos0` *angle*

Sets the orientation at the 5′-most nucleotide to *angle*.

`set_dir pos0` *angle* `f`

Sets the orientation, and causes stems to be flipped (reflected along the axis of their middle) (This is caused by the extra '`f`'.)

5.8.2.2 Laying out arbitrary units like a bulge

`bulge` *label*

Position the structural element starting at *label* as a bulge. (Note: implicitly, the bulge is placed between the position immediately before the nucleotide at *label*, and the position immediately after the drawing unit containing *label*. In earlier versions of R2R, the user could set the before & after points explicitly, but I found this was never needed; the added flexibility just allowed the user to draw things in the reverse direction, and make errors.)

If N single-stranded nucleotides are laid out as a bulge from nucleotide X to nucleotide Y, then the computer calculates a circle such that:

- X and Y are points on the circle
- The distance between all consecutive nucleotides along the region from X to Y (including the single-stranded region *label*) is the constant internucleotide length.

`bulge_flip` *label*

Same as `bulge` (which was just discussed above) but draw the circle on the other side of the line from nucleotide X to Y.

5.8.2.3 Layout single-stranded loops along a straight line, instead of circle

`layout_straight` *label*

This command forces drawing units that would normally be circular (bulges, internal loops and terminal loops) to be straight. Usually the command is not necessary, because `place_explicit` commands that intersect circular drawing units will cause them to be straight.

5.8.2.4 turn_ss

`turn_ss` *label turnAngle*

Turns a single-stranded region by the given *turnAngle*. The command is equivalent to the following `place_explicit` command:

`place_explicit` *label label*`--` *turnAngle*/2 `1 0 0 0` *turnAngle*

5.8.2.5 turn_stem_at_internal

`turn_stem_at_internal` *label dir optimize-for*

Uses an internal loop to turn the stem 90 degrees. *label* must identify either the 5′-most nucleotide in the 5′ part of the internal loop or the 3′-most nucleotide in the 3′ part of the internal loop). *dir* is either `-1` or `+1`, and this value is multiplied by 90 and added to the current angle of the stem. *optimize-for* is either `L` or `R`, which means optimize the sizes of the circles for the bulges based on either the left or the right sides of the internal loop.

Note: if you have problems with this, you can also try the multistem junction commands. If it doesn't work, try this: split the internal loop into bulges with `internal_loop_to_bulges`, make the bulges as bulges-along-a-circle with `bulge` *label*, and finally use `place_explicit` to manually set where the stem goes.

5.8.2.6 disconnect_from_5prime

`disconnect_from_5prime` *label*

R2R has default rules for positioning elements based on adjacent elements. These rules are overriden by commands such as *place_explicit* or *bulge*.

The `disconnect_from_5prime` command removes the implicit positioning constraint between the structural element at position *label*, and the immediately-5′ element. Positioning will now happen by constraints on the other sides of the elements.

The position at *label* must be the 5′-most position within the given strctural element, or the command will be ignored.

This command is useful in some very specific cases when drawing inline style pseudoknots, where R2R's default layout is inconvenient, and requires setting several `place_explicit` commands to force R2R to do its layout based on the 3′ of an element (and subsequent elements). The `disconnect_from_5prime` command achieves this in one step.

5.8.2.7 split_ss

`split_ss` *label*

Splits a continuous single-stranded region at internal position *label*. Without this command, the continuous single-stranded region will be treated as one unit in the structure. The consequence of the split is that the two sides can be treated separately. For example, one part could be positioned straight, and the other could be positioned with the *bulge* command.

5.8.2.8 place_explicit

`place_explicit` *label relativeLabel placeAngle relativeVector.X relativeVector.Y absoluteVector.X absoluteVector.Y finalAngle* [`f`]

Positions a nucleotide relative to another. *label* specifies the region that will be positioned. Positioning is done relative to *relativeLabel*. The position of *relativeLabel* is taken as an origin. The following two positions are added to this number, where all coordinates (.X and .Y) are specified in units of the distance between consecutive nucleotides:

- The vector *absoluteVector* is added directly

- We form the unit direction vector in the direction *relativeLabel.angle* + *placeAngle*, where *relativeLabel.angle* is the angle of the backbone at the nucleotide specified by *relativeLabel*. We then travel *relativeVector.X* units in this direction. Then we rotate the unit direction vector by +90 degrees, and travel *relativeVector.Y units*.

The angle of *label* is computed by taking the angle of *relativeLabel* and adding *finalAngle*.

It is possible to use `place_explicit` to place the right nucleotide of a base pair. In this case, remember that the backbone for nucleotides on the right side (3′ side) of base pairs is considered to go in the opposite direction as (180 degrees from) the nucleotides on the left (5′) side. `place_explicit` can also be used to place the last nucleotide in a structural unit (like the last nucleotide of a contiguous stem).

If the optional `f` flag is given at the end of the line, the left/rightness of *label* is flipped relative to that of *relativeLabel*.

Note that `place_explicit` commands can be evaluated in both directions, in a mathematically equivalent way. In other words, if you position label A based on B, there is an equivalent `place_explicit` command to position B based on A. R2R will pick whichever layout order is convenient.

5.8.3 Layout of multi-stem junctions

5.8.3.1 Manual layout

`multistem_junction_bulgey` *label [see below]*

This is a convenience function that allows you to render a multi-stem junction by placing each stem in the junction relative to the previous stem, and then having the computer render the single-stranded junctions as bulges. It is largely equivalent to a series of `place_explicit` and `bulge` commands. The main benefit is that you only have to create one label (i.e., *label*). Note that `multistem_junction_bulgey` also provides some seldom-used functions that do not use `bulge` layouts for single-stranded regions.

label specifies the left nucleotide of the base pair that encloses the multi-stem junction. For examples, see Section 3.6. R2R will complain if you specify something that is not the left nucleotide of an enclosing pair.

By default each junction within the multistem-junction is laid out as if a `bulge` command were used. However, you can specify alternate layouts, as explained below.

After *label* is a series of commands. Each command is one of the following:

- `J`*i anglePlace x1 y1 x2 y2 angleMove*

  where `J` is the literal letter and *i* is a number specifying which stem. The enclosing stem is assumed to have a known position. Then the next stem is positioned relative to the left nucleotide of the enclosing stem pair. This next stem is positioned using `J0`. Then the next stem is positioned using `J1` relative to this stem. This continues until we've done all stems immediately enclosed by the enclosing stem. (Basically we're going around the multi-stem junction from 5′ to 3′). The rest of the `J`*i* command is the same as the `place_explicit` command.

  For `J0`, we position the left nucleotide of the basal pair of the next stem, relative to the left nucleotide of the enclosing pair from the enclosing stem. For `J1`, `J2`, ..., we position the left nucleotide of the basal pair of a stem relative to the right nucleotide of the basal pair of the previous stem. This makes a bit of sense if you think of going around clockwise, or equivalently it's the closest column numbers in the alignment.

- `J/base`*i anglePlace x1 y1 x2 y2 angleMove*

  This is the same as the `J`*i* command just described, except that the placement is always relative to the left nucleotide of the enclosing stem. In other words, both `J0` and `J/base0` place relative to the left nucleotide of the enclosing stem, but `J/base1` also places relativive to this enclosing nucleotide, whereas `J1` places relative to the right nucleotide of the enclosing stem positioned by `J0` or `J/base0`. This function is mainly used by R2R itself, so that R2R can give static layout commands that are equivalent to computationally expensive solved layouts.

- `b`*i* or
  `bf`*i* or
  `bs`*i* or
  `bpe`*i [place_explicit stuff]* or
  `bss`*i* or
  `btriangle`*i corner* or
  `btrianglef`*i corner*

  Here the '`b`' stands for 'bulge'. (It should arguably be "j" for junction, but that is taken for positioning the stems surrounding the junctions.) The bulges/junctions are numbered clockwise starting at i=0. `b`*i* lays out like a bulge (which is the default anyway). `bf`*i* is a flipped bulge (i.e. the bulge goes the other way). `bs`*i* lays the junction out along a straight line like the `layout_straight` command (note: you're responsible for making sure the length is good). `bpe`*i* is an independent command that really only makes sense with `bs`*i*, and it's a `place_explicit` command that positions the bulge itself; you'll usually have to use it with `bs`*i* since otherwise the bulge will go 90 degrees. A short-form for this is `bss`*i*, which both says that the bulge should go straight, and that it should be positioned straight from the base pair (it's the same as `bs`*i* combined with `bpe`*i* `0 1 0 0 0 0`). `blinearstretch`*i* says make the bulge straight, and stretch it between the nucs it has to go. `btriangle`*i corner* says make the region into a triangle (really, two linear segments), with the corner at the position identified by the label *corner* (which must be within the bulge). `btrianglef`*i* is the flipped version, i.e., the corner point goes on the opposite side of the direct line.

- `bspecifyendpoints`*i relDir beforeBulgePos.X beforeBulgePos.Y afterBulgePos.X afterBulgePos.Y*

  Specify the positions of the endpoints of the bulge; the bulged nucleotides will be positioned between them. The endpoint positions are relative to the position of the left nucleotide of the enclosing base pair in coordinates oriented at *relDir*. This is mostly useful for automated layout commands generated by R2R's solver.

- `bdrawcirc`*i*

  Says that the computer should draw the full circle related to bulge i, which is useful for debugging, or for doing touch-ups in Illustrator. If the bulge is defined such that it doesn't use a circular bulge, R2R may fail.

- `backbonelen` *label length*

  Sets the length of the variable-length backbone identified by *label* For example, these two commands would make sense in a given file:

  ```
  #=GF R2R multistem_junction_bulgey J ...backbonelen A 8 ...
  #=GF R2R var_backbone_range A B
  ```

for columns labeled `A`, `B` and `J`.

5.8.3.2 Multi-stem junctions: automatic circular layout

`multistem_junction_circular` *label [see below]*

`multistem_junction_circular_solver` *label [see below]*

`multistem_junction_bulgecircley_solver` *label [see below]*

`multistem_junction_bulgecircleynormals_solver` *label [see below]*

`multistem_junction_bulgecircley_stemnormals_solver` *label [see below]*

These commands are demonstrated and explained at a high level in Section 3.6.1 or Section 3.6.3. The current section explains the details. All of these commands attempt to find a circle on which to approximately position the nucleotides along a multistem junctions.

The parameter *label* must specify a column that is the left nucleotide of the base pair that encloses the multistem junction.

Warning: these commands often don't work very well. It will fairly often produce wacky layouts with nucleotides crossing, which happens if it cannot solve the non-linear program optimization problem. In rare cases, the program will crash. (Note: the selection of a good starting point seems to be important for the solver to solve highly non-linear problems. However, it is difficult to find such a starting point in an automated fashion. The current starting value for the center of the circle seems to work well.) With `_solver`, even slight changes in the initial fraction of stem intersection (e.g the number in the `ai=#` option for stem layout strategy) can be the difference between success and failure. I find the `bulgecircley` commands to be more robust, but it's sometimes necessary to try multiple versions of the `_solver` commands, and fiddle with parameters.

The commands differ as to their strategy for solution. `multistem_junction_circular` uses a highly limited version of the problem, and solves using a simple binary search over the problem defined in one variable (the height of the circle along which the multistem junction is positioned). With `_solver`, a more general version of the problem can be posed in multiple variables, and CFSQP is used. `_solver` will generally take much longer to compute.

The problem the commands solve is also different. `multistem_junction_circular` and `multistem_junction_circular_solver` attempt to solve essentially the same problem: all nucleotides in single-stranded junctions are forced to be on a circle, and the computer attempts to fit the base-paired nucleotides to be close to the circle. The best solution is the one with the smoothest transition between stems and junctions. (Mathematically, the computer is minimizing the sum of squared deviations between the distances of the stem nucleotides and the radius of the circle; perfect smoothness means that the stem nucleotides will lie exactly on the circle.)

On the other hand, the commands `multistem_junction_bulgecircley_solver`, `multistem_junction_bulgecircleynormals_solver` and `multistem_junction_bulgecircley_stemnormals_solver` require that junctions have to lie on some circle, but not necessarily all on the same circle. The computer then tries to find the junctions that fit a common circle as closely as possible. There are two specific ways to do this, which actually seem to give similar results. With `multistem_junction_bulgecircley_solver` the computer measures the distance between points on the junction and the common circle. The points are evenly spaced, and so the computer is using a finite approximation to the integral (which would be hard to compute). The computer minimizes the mean square difference between the distances. With `multistem_junction_bulgecircleynormals_solver`, the computer attempts to minimize the differences between the normals of the common circle curve to the normals in the bulge's circle, at the given points. (The normal is the vector

perpendicular to the circle at a given point. The computer measures differences between normals by the square of their dot products.) `multistem_junction_bulgecircley_stemnormals_solver` only checks for the deviations of the normals at the nucleotides in a stem (i.e., at the ends of a bulge). Note that the `multistem_junction_bulgecircley...` commands do not explicitly deal with circle intersection, and this parameter (e.g. `ai`) is ignored. However, it must still be supplied.

After the *label* is specified, additional commands are in a list, and each command element is as follows:

- `s`*i* *angle strategy*

  You must provide one of these options for each stem. (The computer will tell you if you don't.)

  Tells the computer how to position stem #*i*. The stems are defined differently for the two implementations. For `_solver` , stem #0 is the enclosing stem of the multistem junction, stem #1 is the first stem clockwise after the enclosing stem, and so on, going around clockwise (or equivalently 5′-to-3′). For `multistem_junction_circular`, the enclosing stem has no number, and always fits perfectly on the circle, and stem #0 is the first stem clockwise from the enclosing stem.

  angle is the direction in which the stem points, relative to the left nucleotide of the enclosing stem. For example, *angle*=`90` means that the stem will be pointing clockwise by 90 degrees relative to the angle of the enclosing stem. In the usual layout the enclosing stem is pointing up the page (from the outside of the stem pointing into the multistem junction). Thus, *angle*=`-90` is pointing left, *angle*=`0` is also pointing up the page, *angle*=`+90` is pointing to the right. In the `_solver` commands, setting the direction of stem #0 (the enclosing stem) has the effect of rotating the other stems.

  If you set an *angle* that is not nicely compatible with the tangent of the circle where the stem needs to go, things will look a bit bad (or the computer might not be able to find a plausible solution).

  strategy says how to try to fit the base (outermost pair) of the stem into the circle. *strategy* is ignored for the `bulgecircley` options, but you must provide a legal option. Valid options for *strategy* are as follows:

  – *strategy*=`l` (lowercase '`L`') means that the left nucleotide lies on the circle, and the right nucleotide should go wherever it has to.

  – *strategy*=`r` is the reverse.

  – *strategy*=`m` means that the midpoint of the outer basepair is coincident with the circle.

  – *strategy*=`aa` allows the computer to set the direction of the stem to any angle, allowing the base of the stem to be flush with the circle. This makes the circle look best, but then the stems are not likely to be horizontal or vertical. Note: to ease the implementation you still have to supply some number of *angle*, even though this number is ignored for *strategy*=`aa`. Also, `aa` is not implemented for `_solver`, except for the enclosing stem (It could be implemented for any stem, but there doesn't seem to be a point.)

  – *strategy*=*#*. A number from 0-1 can be specified as the strategy. In this case, the circle will intersect at this point, which is a linear interpolation from zero (left nuc) to one (right nuc). Thus *strategy*=`0` is equivalent to *strategy*=`l`, *strategy*=`0.5` is same as *strategy*=`m`, *strategy*=`1` is same as *strategy*=`r`. Note: *strategy*=`aa` constrains the problem, and with

_solver can avoid some solutions when its applied to the enclosing stem (if you apply *strategy=*aa to the enclosing stem, this constrains the center of the circle to lie on the right bisector of the line between the nucleotides of the enclosing pair, while otherwise the center of the circle is not constrained).

  – *strategy=*ai. ("ai" = "automatic intersect" in this case). Same as *strategy=#*, except the number becomes a free variable in the optimization. Only available for _solver.

  – *strategy=*ai=*#*. Similar to *strategy=*ai, but in this case you specify the starting value before the optimization (from 0 to 1). This can be useful in cases where certain starting values lead to infeasible parameters.

  – *strategy=*ar. Doesn't work well. This is similar to *strategy=*ai, but formulates the problem slightly differently; the free variable is the radius of the left nucleotide to the center of the circle, rather than the circle intersect. I thought it would lead to an easier function to optimize, since the anyangle possibility should be easy to reach, where with *strategy=*ai, the anyangle possibility requires going to the left or right. Unfortunately, *strategy=*ar seems to perform worse.

- `all-stems-anyangle`

  Equivalent to setting *strategy=*aa for all stems. Does not work with _solver commands, since there's no point.

- `fixed_var_backbone_length` *label length*

  Set a fixed length for a variable-length backbone, and do not allow the solver to modify its value. *label* must specify the first label used in the `var_backbone_range` command. For example, given a command

  ```
  var_backbone_range X Y
  ```

  you might set

  ```
  multistem_junction_..._solver ...  fixed_var_backbone_length X 5
  ```

  length is specified in units of `internucleotideLen` (default: 0.105 inches), and can be any real number. Bad values (even negative values) are allowed, but will produce weird results.

- `draw_circ`

  Tells the computer to actually draw the main circle that is the target for layout of the multistem junction. This is useful for debugging (to see what it's trying to do), and might be useful in Illustrator, since it might be helpful to have the circle that you can cut pieces out of.

- `draw_zero_degrees`

  Tells the computer to draw a line in the direction of 0 degrees within the multistem junction _solver command. This is useful as a guide if your enclosing stem is directed in an unusual direction, and you wish to align nucleotides.

- `flipstem` i

  Flips the ith stem, so that it goes inside the multistem junction circle. Useful for saving space.

- `align_stem_horiz` *s t*

  (only with `_solver`) The literals *s* and *t* are stem numbers. The solver will attempt to ensure that the midpoints of their base pairs (that are on the multistem junction) have the same X value (i.e. are horizontally aligned).

  Warning: horizontal is defined relative to the standard coordinate system in which the direction of the left base pair of the enclosing stem is zero degrees. If you are confused, add the `draw_zero_degrees` directive.

  Warning: adding alignment constraints can easily make the problem over-constrained, and the computer might not be able to achieve all constraints, or might end up with a completely messed-up layout.

- `align_stem_vert` *s t*

  Similar to `align_stem_horiz`, but align vertically.

- `align_angle` *angle s t*

  Similar to `align_stem_horiz`, but aligns at an arbitrary angle, specified by *angle*. The command `align_stem_vert` *s t* is equivalent to `align_angle 0` *s t*, while `align_stem_horiz` *s t* is equivalent to `align_angle 90` *s t*.

  If *angle* is the letter `h`, then alignment is horizontal. If it's `v`, then alignment is vertical.

  Mathematically, the computer is projecting the base pairs' midpoints onto the line defined by *angle* using a scalar projection, and constrains the projected positions to be the same.

- `align_nuc_centers_angle` *angle list1 list2*

  A more general version of `align_angle`. *list1* and *list2* each specify a set of one or more nucleotides. The computer will calculate the midpoint (arithmetic mean) of each of these nucleotide sets, and force the midpoints to be aligned. The nucleotides within the sets can be any nucleotide within the multistem juction.

  list1 and *list2* are specified by a space-separated list of labels that are each terminated by a dot ('`.`'). The first thing in either list can be `circle-center`, in which case the center of the circle used in optimizing the multistem junction will be used. `circle-center` must be first in the list if it is used. (See `demo/THF/THF.sto` for an example of its use.)

  If *angle* is the letter `h`, then alignment is horizontal. If it's `v`, then alignment is vertical.

- `try_harder`

  Try harder to solve the problem, by trying different initial values of certain variables, and seeing which leads to the best solution. The variables modified are `initial_radius` and `initial_first_point_angle` (see below).

  For `multistem_junction_circular_solver`, I have encoded a few different values that have worked in some circumstances. The list is, of course, not complete.

  For the `..._bulgecircley_...` commands, I have not come upon any cases with a strong need for alternate values. Therefore, `try_harder` has no effect on these commands.

- `initial_radius` *number*

  Specifies the circle radius (in inches) that the solver will use as an initial value in its optimization. The starting values of variables can make a big difference in whether the optimizer

ends up with a good solution, an acceptable but not ideal solution or a complete mess. Sometimes you can get good starting values (where necessary) from less-constrained problems that the computer has solved. The computer will output the optimal circle radius after solving a problem. The default is `0.459181`.

Note: I have considered trying to set initialization values like this using the simpler **`multistem_junction_circular`** function. However, this function won't take into account **`var_backbone_range`** regions, whose lengths can be changed, so it won't be a general solution.

- `initial_first_point_angle` *number*

  (Only applicable to `multistem_junction_circular_solver`.) Specifies an angle in degrees of a nucleotide immediately $5'$ to the right nucleotide in the enclosing base pair. The solver will use this angle as an initial value in the optimization. The starting values of variables can make a big difference in whether the optimizer ends up with a good solution, an acceptable but not ideal solution or a complete mess.

  Sometimes you can get good starting values (where necessary) from less-constrained problems that the computer has solved. The computer will output the optimal first angle value after solving a problem. The default is `70`.

- `initial_var_backbone_length` *label length*

  Set an initial value for the variable-length backbone length, to help the optimizer reach a good solution. *label* must specify the first label used in the `var_backbone_range` command, as described under `fixed_var_backbone_length`, above. *length* is specified in units of `internucleotideLen` (default: 0.105 inches), and can be any real number. However, the solver will constrain it to be at least `1`. The default initial length value is `2`.

All commands will optionally append to a file a `multistem_junction_bulgey` command that statically models the layout determined by the solver. This will happen if the `R2R_DUMP_FILE` shell environment variable is set, and this environmental variable specifies the path to the file.

With `_solver`, the computer will print some additional diagnostics. It will tell you the coordinates of the center of the circle and its radius (mainCircle =). Note: these coordinates are in inches, and they're in a coordinate space where the left nucleotide of the enclosing stem is at the origin (0,0), and the vector from this left nucleotide to the right nucleotide is (1,0). Up is negative in the Y dimension.

It will also tell you the result of the objective function with the optimal variables. The objective function is broken into these components:

- `circleJoinedValue`: how close the total angles in the circle are to 360 degrees. This should be very close to zero.

- `alignConstraintObjective`: irrelevant. (Alignments are now specified as non-linear constraints.)

- `changeInRadiusSum`: the main optional number. My feeling is that it's best if there's as few as possible changes in the radius (distance from the circle center) that can arise between stems and junctions. Essentially this makes the circle as smooth as possible.

- `rightNucFakeToActualDist`: essentially another way of looking at circleJoinedValue

- `stem # intersect fraction`: the final value (between zero and one) with s# *angle* ai option.

- `junc #, text-col # (raw #) var backbone length = # :` the lengths chosen for var_backbone_range units in the junctions. The length units are angular, and 1 unit is equal to the rotation in angle caused by the default internucleotide length with the given circle radius.

The output will also show any constraints, and the value achieved for them.

5.8.4 Changing layout of secondary structure

5.8.4.1 depair

`depair` *label [label. . .]*

Assumes that *label* is the left nucleotide of a base pair. Removes the pairing. Useful for when one side of the pair should be a gap, but not the other side. *label* can also specify a set of left nucleotides (like with `SS_cons:<`). Multiple *label*s can be given, separated by spaces. For example, to remove all the pairs in a pseudoknot corresponding to line `SS_cons_1`, use:

```
depair SS_cons_1:<
```

5.8.4.2 make_pair

`make_pair` *left-label right-label* [*ss-name*]

Makes the nucleotide *left-label* base pair with the nucleotide specified by *right-label* by altering the secondary structure. By default the alteration will happen in the primary secondary structure (i.e., `SS_cons`), but the optional *ss-name* can specify an alternate secondary structure line to use.

In theory, you can specify multiple nucleotides in each of *left-label* and *right-label*, and the corresponding pairs (in reverse order for *right-label*) will become base pairs. Thus, again in theory, you can specify a stem at once. However, in practice, I haven't tested this functionality.

5.8.4.3 Internal_loop_to_bulges

`internal_loop_to_bulges` *label*

When *label* is the beginning left nucleotide of an internal loop within a hairpin, splits each side of the hairpin into bulges, so that they can be manipulated independently. This is also important if you want to use the command `bulge` *label*.

5.8.4.4 Ignore pseudoknots entirely

`ignore_ss` *ssname*

Entirely ignore the given secondary structure line. For example,

```
ignore_ss _1
```

will ignore the pairings in the line `#=GC SS_cons_1`.

ssname=`primary` is a special name that corresponds to '`SS_cons`' (i.e. the primary secondary structure). This is useful, because Emacs/RALEE gets upset with lines that end in whitespace. Thus,

```
ignore_ss primary
```

will ignore the pairings in the line `#=GC SS_cons`.

5.8.4.5 Ignoring pseudoknots for the purposes of layout

`ignore_ss_except_for_pairs` *ssname detail*

Do not position nucleotides at all based on the given secondary structure consensus line. The secondary structure line is specified in the same way as with the `ignore_ss` command (see immediately above).

detail can have the following values:

- `outline` : outline the nucleotides involved in base-pairing. This is useful for "callout" layouts of pseudoknots.
- `outline-no-bpannot` : same as `outline`, but ignore the *ssname* for the purposes of drawing covariation.

  With `outline`, if *ssname* has covariation at a given nucleotide position, the nucleotide will be shaded green, even if the non-ignored `SS_cons` lines have no covariation. With `outline-no-bpannot`, the ignored *ssname* will not contribute to covariation shading. Thus, with `outline-no-bpannot`, the base pairs will be reflected only in the fact that they'll be outlined and there'll be an indication like 'pk_1' next to them.
- `outline_only_bp`, this is similar to `outline`, but it only outlines the nucleotides that are really involved in a basepair. By contrast, outline will attempt to also outline bulges.
- `ignore`, then don't do anything with the base pairs.

5.8.4.6 subst_ss

`subst_ss` *replace-ss into-ss*

deletes the `SS_cons_`*into-ss* line, replacing it with whatever is in `SS_cons_`*replace-ss*, and deleting `SS_cons_`*replace-ss*. '`primary`' is a special name that corresponds to '`SS_cons`' (i.e. the primary secondary structure). This is useful, because Emacs/RALEE gets upset with lines that end in whitespace. Useful for drawing a pseudoknot pairing, where the pseudoknot pairing should be the main pairing.

In `oneseq` mode, a line like `#=GR` *hitId* `SS_cons_`*name* can also specify a secondary structure line, when working with the hit *hitId*. See `merge_ss` (next) for an example.

5.8.4.7 merge_ss

`merge_ss` *replace-ss into-ss*

Same as `subst_ss`, but merges the base pairs in, instead of clobbering. WARNING: the code does not check if the base pairs are compatible (i.e., if they're pseudoknots relative to each other).

This command is useful if you want to keep a terminator as a separate `SS_cons`, which means that when you use the `#=GC ACTUAL_MOTIF` line, you won't get only half of the terminator (which would make the structure line invalid because it would have unmatched brackets). However, by using `merge_ss`, you can put the terminator into the drawing with R2R.

In `oneseq` mode, a line like `#=GR` *hitId* `SS_cons_`*name* can also specify a secondary structure line, when working with the hit *hitId*. For example, given the line:

```
#=GR NC_018081.1/1633710-1634138 SS_cons_TT
```

you can then have the command

```
#=GF R2R ifdefeq oneseq NC_018081.1/1633710-1634138
#=GF R2R merge_ss _TT primary
#=GF R2R endif
```

which will add a hairpin (TT=transcription terminator) to the primary secondary strcture.

5.8.5 variable-length regions

5.8.5.1 var_hairpin

`var_hairpin` *leftpair rightpair*

Replace the terminal loop contained within *leftpair* and *rightpair* with a variable-length hairpin symbol. *leftpair* and *rightpair* must be base-paired with each other (so, I don't know why I require the user to specify both. . .)

5.8.5.2 var_term_loop

`var_term_loop` *leftpair rightpair message*

leftpair and *rightpair* must be the matching base pairs of the last base pair before the terminal loop.

Largely the same as `var_backbone_range`, except that I think it draws a nicer circle in the special case where you're replacing the whole terminal loop.

5.8.5.3 Variable-length backbone

`var_backbone_range` *firstLabel lastLabel [text]*

Replace the nucleotides from *firstLabel* to *lastLabel* with a variable-length backbone. The nucleotides within the *firstLabel* and *lastLabel* columns are included within the variablen-length region. All nucleotides within the variable-length region should be single-stranded, or R2R will report an error that base pairs are broken. (However, you can use the `ignore_ss` command to remove pairing that complicates the use of the `var_backbone_range` command. This strategy is demonstrated in the file `demo/104/SAM-I-IV-variant.sto`.)

The variable-length region will be drawn with a thick black line. The line will be labeled with *text*. If *text* contains the special string `ntrange`, then the occurrence of `ntrange` is replaced with the text "x-y `nt`", where x-y is the range of the number of nucleotides within the variablen-length region. If *text* is not given, then it simply gives the range "x-y `nt`". (This is the functionality I normally use.) You can include the text "␣`\n`␣" (where ␣ is a single space), and this will put the remaining text on another line.

Note: if you're replacing an entire terminal loop, use `var_term_loop`, as it usually looks nicer.

Note: after applying a `var_backbone_range` command, the *lastLabel* will no longer work, but you can refer to the region using the label *firstLabel*.

Note: in single-stranded and straight regions, the length of a `var_backbone` is determined by the size of the text that it's labeled with. But, in circular regions (bulges or other loops), the `var_backbone` length is fixed (by default 3), regardless of the size of the text.

`var_backbone_range_if_5prime_nuc_exists` *firstLabel lastLabel text*

Same as `var_backbone_range`, but only count sequences where there is at least one nucleotide that is 5′ to the variable backbone. This is to account for sequences that are truncated (like environmental sequences) that shouldn't be counted.

`var_backbone_range_size_fake_nucs` *numNucs firstLabel lastLabel text*

Same as `var_backbone_range` , but size the backbone as if it contained *numNucs* nucs. This is silently ignored if the backbone is in a straight region (i.e., not an internal loop, terminal loop or bulge). *numNucs* can be any positive number, not just integers, but it will usually look bad if it's less than `1`.

5.8.5.4 var_stem

`var_stem` *leftOuterLabel leftInnerLabel rightInnerLabel rightOuterLabel*

Replaces a stem region with a variablen-length stem. The original stem region is allowed to contain bulges/internal loops, but not terminal loops. The region is defined by the closed intervals [*leftOuterLabel*,*leftInnerLabel*] and [*rightInnerLabel*,*rightOuterLabel*], which are assumed to pair to each other (*left* pairs with *right*).

It is required that *leftOuterLabel* and *rightOuterLabel* must pair with each other, although this restriction could be relaxed with improvements in the code.

5.8.6 Annotation

5.8.6.1 Tick labels (like the nucleotide numbering in cleavage diagrams)

`tick_label` *label text*

Annotate the nucleotide identified by *label* with *text*. The *text* is connected to the nucleotide with a tick mark (i.e., a short line). Within *text*, you can include the text "␣\n␣" (where ␣ is a single space), and this will put the remaining text on another line. Thus, the command

```
tick_label A one line \n two line \n red line \n blue line
```

will take 4 lines.

`tick_position_label_names`

Useful for debugging and helping you work out where things are. Labels nucleotides with all valid labels (except for the `SS_cons` implicit labels, like `SS_cons:<`).

`tick_label_regular_numbering` *start skip* [`zero`] [`firstNucNum` *num*]

Uses the tick-style labeling to label nucleotides with their number. The nucleotide position *start* is labeled, as is *start+skip*, *start+2*skip*, ... When one sequence is printed (like to show ligand-dependent changes in cleavage), there is implicitly *start*=`0`, *skip*=`10`. Note that the first, 5′-most nucleotide is by default number 1, so in the `oneseq` case, the 5′-most nucleotide is not labeled, and the first labeled nucleotide from the 5′ end is nucleotide number 10.

You can change the number of the first nucleotide. If you put `zero` at the end, then the first nucleotide is numbered zero instead of one. If you put `firstNucNum` *num* at the end of the line, then the first nucleotide has the number *num*. Putting

```
firstNucNum 0
```

at the end of the line is equivalent to using `zero`. And the line

```
#=GF R2R tick_label_regular_numbering 0 10 firstNucNum 1
```

is the default behavior.

Using `zero` is mainly useful if you're using the labels to aid in debugging the C++ code comprising R2R, since the R2R program uses zero-based numbers internally. Setting other numbers is useful if you're trying to match numbers from a previous figure.

The use of this command disables the default numbering of nucleotides in `oneseq` mode. However, you can always add this in explicitly with `tick_label_regular_numbering 0 10`

```
tick_label_disable_default_numbering
```

Disables the numbering of nucleotides that is done by default when you run R2R in `oneseq` mode.

5.8.6.2 nobpannot

```
nobpannot
```

Do not shade any base pairs. Normally, R2R will shade base pairs in consensus diagrams to annotate them as covarying, compatible, etc.

5.8.6.3 Pseudo-bold fonts

`thick_stroke_font` *label stroke-width*

Draws nucleotides matching *label* with stroking, which has the effect of making them look bold. (R2R cannot select bold fonts directly.)

Note: this feature has not been implemented with SVG output. It only works with PDF.

5.8.6.4 Changing nucleotide colors

`nuc_color` *label color*

Sets the font color of the nucleotide(s) specified by *label*.

5.8.6.5 Circling nucleotides

`circle_nuc` *label color* [`width` *width-in-points*]

Draws circles around all nucleotides with *label*. The style of the circling is like for cleavage diagrams. The optional width directive specifies the thickness of the line forming the circle. (By default it's the same as for the cleavage diagram.)

note1: To accommodate the circles, the size of nucleotides is reduced by a constant factor defined by `nucShrinkWithCircleNuc` (default: `0.8`), and the size of bond lines by a factor of `pairBondScaleWithCircleNuc` (default: `1`, so no actual change). To eliminate this behavior, or find alternate measurements that work, use the `SetDrawingParam` command; see Section 5.5.3.

5.8.6.6 Boxing nucleotides

`box_nuc` *label color* [`width` *width-in-points*]

Draws boxes (rectangles) around all nucleotides with *label*. The optional width directive specifies the thickness of the line forming the box. (By default it's the same as for the cleavage diagram.)

5.8.6.7 Outlining/inlining nucleotides

`outline_nuc` *label color*

Draws outline around the outside of all nucleotides with *label*. This is the same look as for drawing pseudoknots.
color is currently ignored.

`inline_nuc` *label color*

Same as `outline_nuc`, but draw the line on the other side.
Warning: R2R will attempt to automatically join the lines or arcs that comprise an outline. However, sometimes its heuristics will fail, and it might be missing some lines. In this case, try disabling the automatic joining behavior:

```
SetDrawingParam outlineAutoJoin false
```

5.8.6.8 Boxing a set of nucleotides

`box_label` *label X Y text*

Draws a bounding box in gray around the nucleotides identified by *label*. The box is labeled with *text*, in the direction specified by *X,Y*. *X* and *Y* are both either `-1`, `0` or `1`. For example,

```
box_label L 1 0 this is a box
```

would box the nucleotides identified by the label `L`, and put the text "`this is a box`" immediately to the right of the box.

5.8.6.9 Shading nucleotides along the backbone

`shade_along_backbone` *label* [*label2 ...*] *color*

Draws a thick line along the backbone defined by the nucleotides belonging to any of the *labels* in the list. The last parameter is always the color of the shading. The line is thick enough to fully shade the nucleotide letters, so its width is implicitly set by the nucleotide font size.

Because R2R is not programmed to handle all cases, there will be abrupt changes in the direction of the shading line when joining nucleotides in some directions. These abrupt changes are usually not very noticeable.

Note: you can use the shading produced to highlight a set of nucleotides in other ways. The `shade_along_backbone` command creates a complex path (series of lines and arcs) that can be manipulated in drawing programs. Also, for example in Adobe Illustrator, select the line and use the "Outline stroke" command (under the Object/path menu in Illustrator CS) to convert the line to an outline shape, which you can then manipulate.

5.8.6.10 Outlining a stretch of nucleotides around both ends

`outline_along_backbone` *label* [*label2* ...] *color*

This command has the same syntax and idea as `shade_along_backbone`, except that it creates an outline around the nucleotides.

There are limitations of this command. First, the method is somewhat of a hack: it simply uses the code for `shade_along_backbone` to draw a larger shade in the outline color, then uses the code again to draw a smaller white shade. What's left is the outline, and there's no need to calculate the actual outline.

The limitation is that you can't draw the outline on top of anything, because the white-out shading would obscure whatever's below. Therefore, the outline is drawn below any covariation shading. This often looks okay (and isn't a problem for unpaired nucleotides), but sometimes does not look good.

One workaround is to use the `shade_along_backbone` command, then use a drawing program to convert to an outline. For example, in Adobe Illustrator the "outline stroke" command will do this conversion.

Note: users who are interested in implementing a more general solution, might wish to use the code in the `2geom` subdirectory of the Inkscape source code. This code can create shapes that are unions of simpler shapes, and so it should be possible to create robust outlines of arbitrary regions. I have not implemented this strategy in R2R, however. A complication is that it will be necessary for the user to specify whether they want the inside of an internal/terminal loop to be a part of the shape, or not. In general, I find it easier to use `shade_along_backbone`, and then manipulate the shape in Adobe Illustrator.

5.8.6.11 Shading the backbone in skeleton drawings

`shade_backbone_skeleton` *label color*

This command is only relevant to `skeleton` mode drawings. It is similar to `shade_along_-backbone`, but shades the backbone itself. Normally the backbone is drawn in black.

Note that the use of different colors for the backbone in skeleton drawings often does not look good because the isolated base pairs are small circles. It can help to change the drawing style using this command:

```
SetDrawingParam alongBackboneStyle 1
```

(see Section 5.5.3). The feature is used experimentally for the HEARO RNA [15], with output in `demo/output-svg/HEARO.svg` or `demo/output-pdf/HEARO.pdf`.

5.8.6.12 Drawing circles associated with loops

`draw_circ` *label [label...]*

Draws full circle for layout involving the nucleotide at the given *label*. This command is useful for internal loops, terminal loops and bulges. For multistem junctions, use the *draw_circ* directive associated with the `multistem_junction_circular_...` commands.

These circles are sometimes useful in preparing other annotation in a general-purpose drawing program.

5.8.6.13 Drawing direct lines between consecutive nucleotides.

`straight_line_3prime` *label [label...]*

Draws the straight line connecting the center of one nucleotide to the next nucleotide 3′ to it.

5.8.7 Miscellaneous

5.8.7.1 Override default parameters for drawing

`SetDrawingParam` ...

The syntax for this command is the same as the `SetDrawingParam` command in the `.r2r_meta` file, except that names and parameters are space-separated, as are all R2R command parameters within the Stockholm file. For details on parameters, see Section 5.5.3.

Note: due to the way R2R processes files, changing a drawing parameter in one line of a file might affect the use of that parameter in earlier lines of the file.

5.8.7.2 No 5′ label

`no5`

does not draw the 5′ label on the molecule. Useful for drawing pseudoknots and some modular structures.

5.8.7.3 Adding G for transcription

`g_for_transcription` *number*

Add G residues to the 5′ end of the sequence in the alignment. The number of Gs to add is given by *number*. Each G has an asterisk added to it as if with the `tick_label` command. All of these added Gs gets the special label "`g_with_transcription`", so you can refer to them by this label. Non-genomic G nucleotides are often added to the 5′ end of RNAs because it increases the yield of *in vitro* transcription using T7 RNA polymerase.

5.8.7.4 Keeping gap columns in the drawing

`keep` *label1 label2 . . .*

Forces alignment positions corresponding to any of the listed labels to be kept in the alignment, even if they would normally be considered gaps and removed.

5.8.7.5 Breaking pairs

`depair` *label [other labels...]*

Converts the columns matching any of the given *label*(s) to single-stranded. It will be as if these nucleotides were never annotated as base paired.

For example, this command removes all base pairs in the `#=GC SS_cons_1` line:

```
depair _1:< _1:>
```

5.8.7.6 Explicitly setting/overriding the covariation shading

This command allows you to explicitly set the covariation shading of base pairs. The setting will override whatever value is set in the input Stockholm-format file that was determined by a covariation analysis.

You can already modify covariation shades by modifying `#=GC` lines in the Stockholm file, but in some cases it's convenient to do this in the original .sto file.

`set_covary_shade` *ssName label shade*

where

- *ssName* : identifies the `#=GC SS_cons` line to modify. The text "`primary`" identifies the `#=GC SS_cons` line. To identify a line `#=GC SS_cons`*something*, use "*something*" (without the quotes) as the value of *ssName*.
- *label* : a valid label identifying positions to modify. These positions must be the left (5′) nucleotide of a base pair. Modifying the right nucleotide has no effect.
- *shade* :
  - `?` : nothing. This is typically not shaded.
  - `0` : no mutations. This is typically shaded red.
  - `1` : compatible mutations. Or R2R covariation, if R-scape is being used. This is typically shaded blue.
  - `2` : covariation. This is typically shaded green.

5.8.7.7 Deleting columns using an explicit command

`delcol` *label1 label2* . . .

Deletes the columns specified. One more more labels may be specified. The effect of this command is equivalent to putting a dash character in the `R2R_LABEL` line, except that the `delcol` command can be applied conditionally using `ifdef` or `ifdefeq` commands.

5.9 Troubleshooting

NOTE: R2R will parse the RNA structure into units, and commands only apply to the first nucleotide in a unit. See Section 5.3.

- Use the `tick_label` command to label nucleotides and make sure you have the right one (or find out where it is), like "`#=GF R2R tick_label_regular_numbering 1 1 zero`"
- cyclic dependencies (very rare)
  - try using `ignore_ss_except_for_pairs` *SS_cons-code* `outline` for any extra `SS_cons` lines, and see if the problem recurs. For example, if you have `#=GC SS_cons_1`, use `ignore_ss_except_for_pairs _1 outline`

- `mark_flip`

  This R2R command marks nucleotides that are flipped (left vs. right) with "f", and un-flipped nucleotides with "=". Only the 5′-most nucleotide in each structural unit (stem, bulge, etc.) are marked.

- You can get R2R to show extra information about how it's using `place_explicit` commands. See section 3.8.3 for examples, and use the following command:

  ```
  #=GF R2R SetDrawingParam showPlaceExplicit true
  ```

- You can get R2R to show you the direction of the backbone at each nucleotide using:

  ```
  #=GF R2R SetDrawingParam showEachNucleotideDir true
  ```

5.10 Text output of r2r useful in debugging

The `r2r` program produces output that might help in debugging problems with your input (or indeed the R2R code). Sorry, there's no `--quiet` option.

5.10.1 Start/end of file

For each Stockholm input file, R2R will print

`PROCESSING:` *file-name*

When it finishes parsing and layout of the RNA, it will print

`DONE` *file-name*

Note that the actual drawing code is performed after parsing/layout of all structures, and some error conditions are only detected in drawing.

5.10.2 consensus lines and "raw" coordinates

Next, R2R will draw the consensus sequence line, after columns are removed because of gaps, explicit deletion or variable-length functions. The codes for the consensus line (e.g. `nnnRGGACGACC`) are explained in the previous chapter for the `#=GC cons` line. The consensus line defines the "raw" coordinates, which are numbered from 5′ to 3′, starting at zero.

Next, all `SS_cons` lines are output (again after removal of columns).

5.10.3 Preliminary secondary structure drawing units

Next, R2R will dump its preliminary secondary structure units, called "`ssContextList`". This is immediately after parsing, and does not take into account any splitting of drawing units that will be done later for `place_explicit` commands.

For each unit, it will print something like this:

```
[0,8:12,20) T,F,T Pair
```

In this example, the `[0,8:12,20)` defines the nucleotide positions of the drawing unit, in raw coordinates. `[0,8:` is the left (5′) side of the unit, and is a half-open interval. In other words, it is the set {0,1,2,3,4,5,6,7}, thus including the first number (`0`), but not including the second (`8`). Similarly, `:12,20)` defines the right side of the unit, and is also a half-open interval.

The `T,F,T` defines flags that are not important.

The `Pair` defines the type of drawing unit. The following are types:

- `Pair` : stem
- `Outside` : single-stranded region outside a pair. (`Outside` is also sometimes used for single-stranded regions within a hairpin that should be positioned on a straight line, instead of on a circle.)
- `InternalLoop` : internal loop or bulge. At this stage, also includes some outside regions that are between (but not contained) in stems. These will be fixed to type `Outside` later.
- `TerminalLoop`

5.10.4 place_explicit links

The next section of the file shows how the drawing units are linked together by `place_explicit` commands, or by implicit rules.

For example, here is one item of output:

```
ssContext
                [147,172;inf,=] {raw [0,23;109,109) }  Outside
        link
                posFrom: 22 , [147,172;inf,=] {raw [0,23;109,109) }
                posTo  : 23 , [173,177;278,282] {raw [23,28;82,87) }
                default rule
                -45 (1,0) -90
```

The `ssContext` line introduces a drawing unit whose links will be listed. The `raw` specification and the meaning of `Outside` were explained above.

The text `[147,172;inf,=]` defines the drawing unit in terms of text columns within your input alignment file. (As always, the left-most text column is numbered zero, to follow emacs.) The equals sign (`=`) says that the right part of the drawing unit is empty. The range `147,172` is a doubly closed interval that includes text columns 147 and 172, and the columns between them.

The `link` introduces a `place_explicit`-style link, showing how the drawing units can be positioned relative to one another. `posFrom: 22` specifies that raw position 22 will be the nucleotide position within the first drawing unit. `posTo : 23` species that position 23 is the nucleotide position within the second drawing unit, whose nucleotide ranges are given. `default rule` means that this is the default placement, which can be overridden with a `place_explicit` command. `-45 (1,0) -90` defines the `place_explicit`-style coordinates, where `-45` is the placement angle, `(1,0)` is the relative units to travel, and `-90` is the relative angle of the next unit. Thus, this is analogous to the following `place_explicit` command:

`place_explicit` *position-23 position-22* `-45 1 0 0 0 -90`

The text “`involvesCircularLayout==true`” (within a `link`) signifies that the `posFrom` or the `posTo` drawing unit is an internal loop that will be laid out along a circle.

Links relating to actual `place_explicit` commands look like this:

```
link
        posFrom: 32 , [169,174;272,277] {raw [27,33;110,116) }
        posTo  : 36 , [181,185;198,202] {raw [36,41;51,56) }
        place_explicit demo/104/leu-phe-leader.cons.sto:67
        0 (0,0) -180
```

The file name and line number of the `place_explicit` command are given. The absolute shift coordinates are not shown.

5.10.5 Selecting the place explicit commands

Eventually, you will see lines like "`Calling PositionBackboneElement for this place_explicit:`". These introduce the `place_explicit`-style links that R2R selected to place the drawing units. There is a special link called `dummy rule to position first element` that gives the position of the 5′-most drawing unit.

5.11 Interacting with R2R using scripts

This section discusses feature that are useful if you want to generate R2R input by scripts, or if you want to extract layout information from R2R.

5.11.1 Generating R2R input using scripts

Scripts can explicitly select a layout for R2R using the `place_explicit`, `set_dir pos0`, `bulge` and `bulge_flip` commands. These commands are described elsewhere in this chapter and in the tutorial. An example input file is in the directory `demo/pedagogical/ScriptInputExample.sto`.

First, set the orientation of the backbone at the 5′-most position.

`set_dir pos0` *angle-in-degrees*

(see section 5.8.2.1 for more on this command, and also how to flip the drawing so that stems are drawn in a mirror image.)

Then set the position of key nucleotides using the `place_explicit` command (see section 5.8.2.8). It will probably be most convenient the orient nucleotides relative to the 5′-most nucleotide and to refer to alignment positions by their numbers. For example, to set alignment position 7 (note: the 5′-most column is numbered zero) to coordinates (4,5), oriented at an angle of 75 degrees relative to the angle of the 5′-most nucleotide, use this R2R command:

```
place_explicit #:7 pos0 0 0 0 4 5 75
```

Note:

- distance units are in terms of the (generally constant) distance between nucleotides, by default 0.105 inches. See the `internucleotideLen` drawing parameter on page 52. Thus, by default, coordinates (4,5) in the above `place_explicit` command correspond to (0.42,0.525) inches.
- The positive X direction is to the right, the positive Y direction is down.

- When nucleotides X and Y are base paired, the position of Y depends on the orientation angle of nucleotide X, and the drawing parameter defining the distance between paired nucleotides (see `pairLinkDist` in section 5.5.3). Only one of the paired nucleotides should be positioned. An exception is for pairs that are ignored (e.g., by the `ignore_ss` command); these nucleotides are not treated as being base paired by R2R for the purposes of determining the layout.

Use `bulge` or `bulge_flip` (see section 5.8.2.2) to position nucleotides on a circle. These commands will correctly set the orientation of each nucleotide in the bulge, and will consider the backbone to lie on an arc. It is also possible to set the position of each nucleotide individually using `place_explicit` commands, but R2R will not realize that the nucleotides are on a circle. This will affect the behavior of some commands, e.g., `shade_along_backbone` and `var_backbone_range`. Currently, no command is implemented that would allow the input to specify a circle explicitly.

5.11.2 Accessing layout information from R2R using scripts

R2R can dump information on the data it will draw, including which alignment positions were not drawn (because they're gappy or because of a user command) and the coordinates of the nucleotides. The format is a tab-delimited file with multiple sections.

To direct R2R to output this information, set the `dumpInfoFile` drawing parameter (see section 5.5.3) to the desired output file path.

The format of the file is as follows. The file is tab-delimited, and any whitespace below is actually a tab character. The file contains multiple sections. Each section begins with a header line, giving a name of the section. All headers except for the first (`drawingName`) specify the number of lines contained in subsequent lines of the section. So, to read this file, read a line to determine the section and number of lines of data, then read that number of lines, then read the next header line, etc.

An R2R output file (PDF or SVG) can contain multiple drawings in it. For example, you might have a consensus diagram, a consensus of one or more pseudoknots and a single sequence (`oneseq` mode). All of these drawings will appear in the same dump file. The order of sections in the file is always the same, except that the sections will be repeated for each drawing.

The sections and their order are as follows:

- Header line: `drawingName` *name*

  The name of the drawing whose data will come next. The drawing name is given in at the top of each drawing in R2R's PDF or SVG output.

  There are no additional data lines in this section, just the header line.

- Header line: `posToAlignCol` *num-data-lines*

  R2R removes alignment columns from the drawing when they are gappy or as a result of some commands (e.g., `var_backbone_range`). This section defines the mapping from positions in the drawing to columns in the original input alignment. The 5′-most position in the drawing is numbered zero, and the 5′-most column in the input alignment is also numbered zero.

  Each data line in the section has two fields:

  - A position index within the drawing.
  - The corresponding column in the original input alignment.

- Header line: `layout` *num-data-lines*

  The fields in the data lines are as follows:

  - Position index of nucleotide within the drawing.
  - The nucleotide letter ('A', 'C', 'G', ...).
  - X coordinate. (Unit length is inches, which is R2R's internal unit.)
  - Y coordinate.
  - Is backbone flipped 180 degrees at this position (like mirror image, e.g., in commands like `bulge_flip`)? (1=Yes, 0=No.)
  - Does the backbone from this position i to the next position $i + 1$ go along a circle? (1=Yes, along a circle, 0=No, along a straight line.) Note: the radius of the circle is equal to the distance from the center of the circle to the position of the nucleotide.
  - If the backbone is linear (i.e., not a circle) at this position, then the orientation angle of backbone at this position, in degrees. Otherwise undefined.
  - If backbone along a circle at this position: X coordinate of the circle's center. Otherwise, undefined.
  - If backbone along a circle, Y coordinate of circle's center.
  - If backbone along a circle, does the next position ($i+1$) also intersect the circle? (1=Yes, 0=No.) In some cases, for example some multistem-junction solver results, the ends of a single-stranded junction are on a circle that does not intersect with the nucleotide at position $i + 1$. This issue only has implications if you want to trace along the backbone of the RNA. (R2R has an ad hoc way of solving this.)
  - Is position actually a variable-length backbone symbol? (1=Yes, 0=No.)
  - Is position actually a variable-length hairpin?
  - Is position actually a variable-length stem?
  - Is position actually a variable-length terminal loop?

Chapter 6

Reference: modular structures

6.1 Sub-families

You can display subsets of a family (with re-normalized conservation stats) to:

- display the base pairs forming a pseudoknot separately
- display distinct sub-types of a motif (like the GRRA/GYRA subsets of GEMM [13, 11])

  Doing this requires running additional commandes; these commands could be automated using a Makefile. Ignoring the issue of automation, you use the script `SelectSubFamilyFromStockholm.pl` to extract the subset based on some predicate, then you calculate conservation stats from it, then you run the generated `.sto` file through R2R.

Examples are given in Section 3.7.

6.2 Using `SelectSubFamilyFromStockholm.pl`

Often pseudoknots and alternate stems cannot be drawn without breaking the RNA backbone. In this case, you need to draw the pseudoknot as a separate structure. The subset predicates allow you to do this.

They also allow you to draw structures that appear only in some RNA sequences, like an optional stem.

6.2.1 Command line

To run `SelectSubFamilyFromStockholm.pl`,

> `perl SelectSubFamilyFromStockholm.pl` *input.sto-file predicate-name* > *output.sto-file*

Flags:

- `-cutEmptyLines` : by default the script will replace lines containing rejected sequences with empty lines. The advantage of this approach is that the line numbers in the file will remain the same. Thus a line number reported in an error during downstream processing of *output.sto-file* will correspond to the same line number as in *input.sto-file*. However, with this flag, blank lines will not be generated.

6.2.2 How to define predicates

You define the predicate within the `.sto` file. To define a column or columns in the alignment, use the following format:

`#=GC SUBFAM_LABEL_`*columns-name*

Then annotate each column where an '`X`' means that column is referenced by the predicate, and a dot '`.`' means it is not.

There are two types of predicates: '`regex`' or '`perl`'.

6.2.2.1 Regex predicates

Regex predicates match all seqs whose selected columns match a given regular expression. The format is:

`#=GF SUBFAM_REGEX_PRED` *predicate-name* [`SUBFAM_STRING` *substString*] *columns-name regex*

where *predicate-name* is the predicate you're defining, *columns-name* designates the columns referenced (which you defined using `#=GC SUBFAM_LABEL_`*columns-name*) and *regex* is a valid regular expression in Perl.

[`SUBFAM_STRING` *substString*] : is optional, if you give it, it will use the alternate string for substituting in changing the file (See 6.2.3). If *substString* is set to `primary` , this will retain the primary modular structure, i.e., it will not modify the file

For example:

```
#=GC SUBFAM_LABEL_GNRA ............XXXXXXX.........
```

(don't take this literally; see the `GEMM.sto` file from Sudarsan *et al.* for specifics)

```
#=GF SUBFAM_REGEX_PRED GYRA GNRA G[CU][AG]A[.][.][.]
```

In this example, we define a set of columns '`GNRA`', which is the 7 nucleotides positions (columns) in the `GNRA` tetraloop of GEMM RNAs. (Sometimes there are more nucleotides in the loop, so that's why there are 7 positions.) Then, we define a predicate '`GYRA`' which sees if those 4 nucleotides match GYRA.

6.2.2.2 Perl predicates

Perl predicates allow you to use arbitrary perl expressions, which are convenient for Boolean logic. The syntax is:

`#=GF SUBFAM_PERL_PRED` *predicate-name* [`SUBFAM_STRING` *substString*] *perl-code*

Currently there aren't that many things you can do with the perl code (without implementing additional capabilities within `SelectSubFamilyFromStockholm.pl`). The *perl-code* used in defining a Perl predicate should end with a `return` statement. At the moment, the *perl-code* can reference the following variables:

- associative array `%predValue` (`$predValue{`*predicate-name*`}`) : evaluates to true or false, where *predicate-name* is the name of a predicate. *predicate-name* can refer to either a "Regular expression" or a "Perl" predicated, but the referenced predicted must have been defined earlier in the `.sto` file than when it is used, otherwise its value will be undefined.

- associative array `%labelToSeq` (`$labelToSeq{`*label-name*`}`) : for the given label, evaluates to the current sequence's nucleotides in the columns associated with the label. Thus, for example, the regex predicate (following the example in Figure 3.15)

  [`SUBFAM_STRING` *substString*] : is optional, and has the same effect as in the `REGEX` case.

  `#=GF SUBFAM_REGEX_PRED HAS_GNRA TERM G[A-Z][AG]A`

  is equivalent to this perl predicate

  `#=GF SUBFAM_PERL_PRED HAS_GNRA return $labelToSeq{TERM} =~ G[A-Z][AG]A;`

- scalar `$hitId` : the identifier of the hit within the original Stockholm alignment file.

- scalar `$seqId` : the identifier of the sequence, but only if the `$hitId` is in the Rfam format, seqId/start-end. Otherwise, this variable will have the value `undef`.

- scalar `$start` : the nucleotide number of the 5′ end of the hit, but only if the `$hitId` is in the Rfam format, seqId/start-end. Otherwise, this variable will have the value `undef`.

- scalar `$end` : the nucleotide number of the 3′ end of the hit, but only if the `$hitId` is in the Rfam format, seqId/start-end. Otherwise, this variable will have the value `undef`.

NOTE: predicate names cannot contain an underscore ('_'). (The problem is that it would be ambiguous in the `SUBFAM_`*pred_label* substitution code.)

For example,

```
#=GF SUBFAM_PERL_PRED other return !($predValue{GYRA} || $predValue{GRRA});
```

Perl predicates also have access to any `#=GS` tags for the current hit, in the map `%gsTag`. This feature is used for GOLLD (see `demo/exceptional/GOLLD.sto`) to eliminate sequences with regions that could not be structurally aligned given the limits of other sequences available for comparative analysis.

The variable `%labelToSeq` is a hash mapping label lines (specified with `#=GC SUBFAM_LABEL_...`) to the sequence of nucleotides or gaps for the current sequence.

The following functions are defined:

- `IsWatsonCrickOrGU(`*$X*`,`*$Y*`)`, where *$X* and *$Y* are strings containing a single character each (i.e., strings of length 1). Returns true iff *$X* and *$Y* correspond to a Watson-Crick or a G-U base pair. (If either character is a gap, then the function returns false.)

- `IsGap(`*$X*`)`, where *$x* is a single-character string (of length 1). Returns true if *$x* is a gap symbol, which is interpreted broadly. *$x* is a gap if it is not an upper- or lowercase letter.

6.2.3 How the file is modified by applying a predicate

Obviously all sequences that match the predicate will be retained, but all sequences that do not match the predicate will be removed. Also the `#=GR` and `#=GS` tags for discarded sequences will also be discarded (caveat: if you use the `SUBFAM_KEEPALL` to force keeping a hit, things might get inconsistent due to a bug in the script).

Also, all lines that begin

```
#=GC R2R
```

or

```
#=GF R2R
```

are removed.

Lines of the form

`#=GC SUBFAM_`*predicate-name*`_`*other-stuff text*

have the "`SUBFAM_`*predicate-name*`_`" part removed, and same thing for

`#=GF SUBFAM_`*predicate-name*`_`*other-stuff text*

The special code

```
#=GF SUBFAM_KEEPALL 1
```

means that all subsequent lines will be kept no matter what. This is turned off with

```
#=GF SUBFAM_KEEPALL 0
```

Also,

`#=GF SUBFAM_`*predicate_name*`_KEEPALL`

keeps lines for the given predicate.

To show pseudoknot pairs as a special other diagram, just define a predicate that matches everything, but use the modifications to define a new `#=GC R2R_LABEL` line, and probably other `#=GF R2R` commands.

You can also override the substitution string using

`#=GF SUBFAM_SUBST_STRING` *predicate-name other-string*

In this case, the script will look for #=GC `SUBFAM_`*other-string*... instead of `#=GC SUBFAM_`*predicate-name*... (and same for `#=GF`). This is useful for multiple predicates that all get drawn the same way.

Note: the `SUBFAM_SUBST_STRING` command must precede the references – the computer does not do multiple passes through the file.

Chapter 7

Meta-Makefile

This chapter explains the use of the `MetamakeDemos.pl` script, which helps to automate the application of the `r2r` command. This chapter assumes that you know what a Makefile is and basically what the `make` command does. It is not necessary to use the `MetamakeDemos.pl` script, since `r2r` commands can be given at the UNIX command prompt directly.

The `MetamakeDemos.pl` script searches for `.sto` files and generates appropriate `.r2r_meta` files, and a `Makefile` to build PDF or SVG output. In order to create `.r2r_meta` files, in some cases you must add additional markup to your `.sto` files. This directs the script to add `oneseq` mode lines, or modular sub-structures, etc.

You can run the resulting Makefile as follows. To create all PDF output files:

```
make all-pdf
```

To create all SVG output files:

```
make all-svg
```

7.1 Running the script

To run the script, you need to designate a directory where you will keep your `.sto` files and `cd` into that directory.

The script is normally run like this:

```
perl MetamakeDemos.pl
```

and must be run to initialize the directory and create a Makefile.

It searches the current directory and its subdirectories for `.sto` files, using the standard UNIX `find` command. However, it does not search the `intermediate` subdirectory (because that is where intermediate files generated by the Makefile will go).

It will create a new file in the current directory that by default is called `Makefile`. It will also create an appropriate `.r2r_meta` file corresponding to each `.sto` file it sees.

7.1.1 Script options

The script accepts the following command-line options, which are not normally used:

- `-srcFileList` *filename* : directs the script not to search for `.sto` files. Instead *filename* gives the list of input `.sto` files. Each line in *filename* specifies an input file, and is tab-delimited. The first field is an actual source `.sto` file. For convenience of Windows/CygWin users, any backslashes in the file name are converted to frontslashes. The second field in the line is a name for the file. The Makefile will copy the source `.sto` file into a new file called `intermediate/`*name*`.sto`, where *name* is the second field.

- `-makefile` *filename* : creates the new Makefile with the name *filename*. (The default is `-makefile Makefile`.).

- `-r2r` *executablefilename* : specifies the command to run for `r2r`. (Default is `-r2r ../src/r2r`.)

- `-subfam` *filename* : references the script `SelectSubFamilyFromStockholm.pl`. (Default is `-subfam ../src/SelectSubFamilyFromStockholm.pl`.)

- `-noDumpFile` : do not get `r2r` to dump solutions of multistem-junctions.

- `-noall` : do not create an `all` target in the new Makefile. This is useful if you want to `include` the Makefile generated by `MetamakeDemos.pl` into some larger Makefile.

- `-doStoTex` : experimental feature.

- `-doStoOutput` : experimental feature.

- `-pubsto` *perl-file* : experimental feature.

- `-cleansto` *perl-file* : experimental feature.

- `-covaryWithRscape` : experimental feature.

- `-covaryUseSsConsWithRscape` : experimental feature, pass `-s` to R-scape to get it to restrict the base pair analysis to the base pairs in `#=GC SS_cons`.

- `-setDrawingParam "`*params*`"` : set drawing parameters (see Section 5.5.3) to different values for all files that the script is aware of. This effectively changes the default values from those that are hardcoded in the R2R source code. The parameter names and values should be separated by space characters. You'll usually need to put this value in quotes because of the spaces. For example:

  ```
  -setDrawingParam "varHairpinNumFakePairs 2 varTerminalLoopRadius 0.14"
  ```

- `-setDrawingParamFile` *file-name* : similar to `-setDrawingParam`, but get the parameters from a file. The parameter names and values should be separated by tab characters (this is different from the case with the `-setDrawingParam` flag). The file must consist of one line (but additional, blank lines are permitted).

- `-inkscapeHelveticaFontName` *fontName* : use this font name in output SVG files. Inkscape is sometimes happier if this name matches a font that it has. The default is "`Bitstream Vera Sans`". This flag arranges for the commands to run `r2r` to add "`--inkscape-helvetica-font-name` *fontName*".

7.1.2 Makefile targets

The script generates a Makefile with the following targets:

- `all` : equivalent to `all-pdf` and `all-svg`. Not recommended in a multi-processor make session because of possible interactions between the solver. Disabled by the `-noall` flag.
- `all-pdf` : generate all PDF output files.
- `all-svg` : generate all SVG output files.
- `clean` : remove intermediate and output files (but keep the `.r2r_meta` files and `Makefile` that were created by the script.).
- `clear-solver-cache` : remove cache files used by the CFSQP solver.
- `solver-pdf` : generate only PDF output files that use the CFSQP solver (and maybe a few that don't, but fool the script). Used for debugging.
- `solver-svg` : analogous to `solver-pdf`, but for SVG output.
- `concat.pdf` : generate `all-pdf` and concatenate the output files together so you can look at them quickly. Mainly just useful for debugging. Requires Ghostscript's `gs` command, which is not distributed with R2R.
- `solver.pdf` : same as `concat.pdf`, but only concatenate the PDF output specified by the `solver-pdf` target.

7.2 Additional markup for .sto files

The script creates an `.r2r_meta` file for each `.sto` input file. The `.r2r_meta` file will always include the `.sto` file in R2R's default consensus mode, but can include other types of runs.

7.2.1 Implicit rules

If you use a `#=GR ... DEL_COLS` tag for any sequence in the alignment, the script will draw that sequence in `oneseq` mode (adding a line to the `.r2r_meta` file).

If you use a `#=GF SUBFAM_PERL_PRED` or `#=GF SUBFAM_REGEX_PRED` directive, the script will instantiate the subfamily alignment using `SelectSubFamilyFromStockholm.pl` (adding a command to the Makefile), and draw it (adding a line to the `.r2r_meta` file). However, you can disable this on a per-predicate basis by adding the line

`#=GF Makefile disablepred` *predname*

7.2.2 Explicit rules

You can explicitly direct the creation of a new type of drawing within the `.r2r_meta` file as follows. This also allows you to `define` additional symbols for use with R2R's `ifdef` and related commands. Note that all fields are space-separated in this file, but the spaces will be converted to tab characters for the `.r2r_meta` file. The file `demo/104/crcB.sto` contains a particularly overwrought array of example usages of these directives.

7.2.2.1 Oneseq

```
#=GF Makefile oneseq ...
```

Directs the script to make a `oneseq`-mode output. For example:

```
#=GF Makefile oneseq NC_003888.3/314159-2653589 define alt-style 1
```

7.2.2.2 Skeleton

```
#=GF Makefile skeleton...
```

Directs the script to make a `skeleton` or `skeleton-with-pairbonds` drawing. As above, additional `define` commands can be given.

7.2.2.3 Defines for consensus diagrams

```
#=GF Makefile define ...
```

Directs the script to make a drawing of the consensus with additional `define` commands.

7.2.2.4 Using define directive with modular structure

`#=GF Makefile pred` *predname* ...

Directs the script to draw a subfamily defined by predicate *predname*, with additional `define` commands (after *predname*). The predicate *predname* must be defined earlier in the file.

7.2.2.5 Oneseq for a predicate defining a modular structure

`#=GF Makefile oneseqpred` *hit predname* ...

Directs the script to draw a subfamily defined by the predicate *predname* in `oneseq` mode for the sequence named *hit*. Optionally, `define` directives can appear after *predname*. The predicate *predname* must be defined earlier in the file.

7.2.2.6 Using a different sequence-weighting algorithm

WARNING: In general, the GSC algorithm seems to be the best available sequence-weighting method. Please read section 4.3.1, which explains the importance of sequence weighting.

By default, R2R will weight sequences using the GSC algorithm. However, with very large alignments, this algorithm can be slow. (In computer science terms, it its time complexity is $O(n^3)$, for n sequences in the alignment.)

To make this faster, especially if precise weighting is not important, you can change this algorithm, by causing the Makefile script to give R2R other flags.

If you add the following to the `.sto` file:

```
#=GF Makefile_Weight_Flag --position-based-weights
```

then R2R will use a much faster weighting algorithm.

You can pass arbitrary flags (in place of `--position-based-weights`), which will be added between `r2r` and `--GSC-weighted-consensus` on the command line.

7.2.2.7 Weighting sequences using your own algorithms with MetamakeDemos

WARNING: please read section 4.3.1 on why you probably should not use this functionality.

To use manual weighting for a given Stockholm file, add a line like

```
#=GF Makefile_SeqWeighting command
```

where *command* is a UNIX command to create a new Stockholm file with the weights. Within *command*, the special text `INPUTSTO` will be changed to the input Stockholm file, and similarly `OUTPUTSTO` will be changed to the output Stockholm file. The result of the command should add the `#=GF USE_THIS_WEIGHT_MAP` tag to the output Stockholm file, as described in Section 4.3. If your Stockholm file already has a valid `#=GF USE_THIS_WEIGHT_MAP` tag in it, you can use the following line:

```
#=GF Makefile_SeqWeighting cp INPUTSTO OUTPUTSTO
```

which just copies the input file (that already has the tag) to the output file.

Chapter 8

R2R source code

8.1 Summary of C++ and Perl source code files

- AdobeGraphics.cpp : Implements generic aspects of interface to draw in graphical files such as PDF-format files.
- AdobeGraphics.h : Interface to draw in graphical files.
- AdobeGraphicsLayout.cpp : Implements generic utility classes to assist in drawing and layout.
- AdobeGraphicsLayout.h : Header file for above.
- AdobeGraphicsPdfLike.cpp : Generic class to factor out similarities between drawing in PDF and SVG.
- AdobeGraphicsPdfLike.h : Header file for above.
- Cm2HmmOptimize_cfsqp.cpp : Implements SolverWrapper interface (see Optimize.h) for CFSQP. (File is originally from my earlier rigorous filter work.)
- CommaSepFileReader.cpp : Reading of files delimited by a single character (e.g., commas). It is used to read tab-delimited .r2r_meta files and space-separated .sto files.
- CommaSepFileReader.h : Header file for above.
- GSCConsensus.cpp : Implements the --GSC-weighted-consensus functionality. The GSC algorithm itself is implemented by the code in NotByZasha/infernal-0.7/squid.
- LabelsAndProjection.cpp : Functions related to parsing .sto files related to projection of columns in an alignment or labeling of columns.
- MiscExceptions.cpp : Generic exception classes, for error handling.
- MiscExceptions.h : Header file for above.
- NoUnderflowDouble.h : Implements real numbers that have a greatly expanded range for their exponent. Used by code related to CFSQP.
- Optimize.cpp : Generic functions for non-linear optimization. Also implements most of the functionality needed for the solver cache.

- Optimize_cfsqp.cpp : Implements a non-linear optimization solver for CFSQP.
- Optimize_nltop.cpp : Implements a non-linear optimization solver for NLOPT.
- Optimize.h : Header file for above.
- ParseOneStockholm.cpp : Functions for interpreting R2R commands in a .sto file. Note: parsing happens in three passes. Pass 1, in which the alignment and column-annotation data is read into data structures, is in the function OneStockholm_try_Pass1. The function OneStockholm_try implements passes 2 (process commands that remove nucleotide positions, e.g. var_backbone_range) and 3 (all other commands).
- ParseSs.cpp : Functions related to parsing RNA secondary structures in bracket notation (i.e. the SS_cons lines).
- PdfGraphics.cpp : Drawing into PDF files. Concrete implementation of AdobeGraphics interface.
- PdfGraphics.h : Header file for above.
- PositionBackbone.cpp : Implements most functions for determining the position of nucleotides within a drawing.
- PositionBackbone_MultiStemCircular.cpp : Implements various methods to draw multistem junctions, except solvers.
- PositionBackbone_MultiStemCircularSolver.cpp : Implements solvers for multistem junctions.
- R2R-Utils.cpp : Utility functions related to parsing .sto files and positioning nucleotides.
- R2R.cpp : Contains main function, with top-level functions. Sets up default values, parses .r2r_meta files, dispatches to other code.
- R2R.h : Declares data structures, classes and functions used by multiple files.
- RnaDrawer.cpp : Classes to actually draw RNA diagrams, based on already-determined nucleotide positions. Includes code to find paths for the shade_along_backbone command or skeleton mode drawings.
- SelectSubFamilyFromStockholm.pl : Perl script to support extraction of a specified subset of sequences from a .sto file, for modular structures (Chapter 6).
- SvgGraphics.cpp : Drawing into SVG files. Concrete implementation of AdobeGraphics interface.
- SvgGraphics.h : Header file for above.
- SymbolicMath.cpp : Implements symbolic expressions, used for CFSQP-based solver of multistem junctions.
- SymbolicMath.h : Header file for above.
- UseDebugNew.h : Header file used in debugging.

- `multiDimVector.h` : STL-style classes for multi-dimensional arrays.
- `stdafx.h` : Include standard system headers.
- `vectorPlus.h` : Extension of STL `vector` class to detect array bounds errors.

8.2 Other information

8.2.1 Overall layout of RNA

To position the nucleotides in an RNA structure, the structure is first decomposed into structural elements: consecutive base pairs, internal loops or consecutive unpaired nucleotides. Default constraints define each element's orientation relative to another element. To apply user-defined constraints in an intuitive manner, a graph is defined whose nodes are structural elements and whose edges correspond to constraints. This graph's minimum spanning tree, with user-defined constraints prioritized over default constraints, yields the positioning constraints to apply. However, some rules direct nucleotides to be drawn on a circle connecting two already-positioned nucleotides, and these rules are applied after the nucleotides are positioned.

Constraints in the graph correspond to `place_explicit` commands. This drawing order logic is applied after any `multistem_...` commands have been resolved into `place_explicit` commands, and is implemented in the file `PositionBackbone.cpp`.

8.2.2 Inferring a path for the backbone

In order to draw a `skeleton`-style schematic drawing, or for shading consecutive nucleotides (e.g., using the `shade_along_backbone` command), it is necessary to determine a path corresponding to the RNA's backbone. In R2R, this path is composed of straight lines or arcs. The path largely follows the lines and arcs used to position nucleotides.

Except when certain commands are used, all nucleotide positions in R2R are laid out either on a straight line, or along a circle, where each nucleotide is centered on the endpoint of the line/arc. For each position, the computer stores the line or circle defined at that point. However, it is not sufficient to use these path segments to connect the diagram, as they are only defined at the nucleotide itself and not for the connection to the next nucleotide. Moreover, even when two nucleotides are connected, the connection might not be aesthetic.

Some nucleotide positions are not really points. For example, the `var_backbone_range` command results in a line or arc, even though it is represented as a nucleotide position. Therefore, the first step is to expand nucleotides into separate points. We call these anchor points, by analogy with the drawing of complex curves in programs such as Adobe Illustrator.

Thus, we are given a set of anchor points $a_1, a_2, \ldots, a_n$. Each anchor point a_i is associated with a specific position on the page, and is also associated with either a line or an arc that intersects that position. However, the line or arc does not necessarily intersect a_{i-1} or a_{i+1}. We wish to find connectors from a_i to a_{i+1} for all i.

If a_i and a_{i+1} are both associated with lines and if the lines are coincident (i.e. the lines are an extension of one another), then we connect a_i and a_{i+1} with that line. Similarly, if they are both associated with arcs that are coincident, we connect the anchor points with that arc.

If a_i is associated with an arc, and if the arc of a_i intersects the position of a_{i+1}, then we connect a_i to a_{i+1} using that arc. In this case, it does not matter whether a_{i+1} is associated with a line or

an arc. Analogous logic applies to the case where a_{i+1} is associated with an arc, and a_i is associated with a line, and the arc of a_{i+1} intersects the position of a_i.

The arc of a_i might not intersect the position of a_{i+1}. For example, the `multistem_junction_circular_solver` leads to bulges that are drawn on a circle but do not (in general) intersect the adjacent paired nucleotides. In cases like this, we declare a join error. A join error denotes cases where R2R is not implemented with logic to join the anchor points nicely. In the event of a join error, a_i is connected to a_{i+1} with a straight line. In practice, these straight lines are not very problematic, because join errors are relatively rare, and because the deviation of the straight lines from an ideal connector is not usually very visible at the scale at which diagrams are drawn.

If anchor points a_i and a_{i+1} are associated with lines, we calculate their intersection. If the intersection is equidistant from the positions of a_i and a_{i+1}, then we join a_i to a_{i+1} using an arc that runs through a_i and a_{i+1}. The radius of the arc is the distance between a_i (or equivalently a_{i+1}) and the intersection point. This case frequently arises in R2R's default layout of stems (e.g., at the $5'$ end of the RNA in Figure 3.3), or when the `turn_ss` command (or equivalent `place_explicit` command) is used. If the intersection point is not equidistant from the two anchor points, we declare a join error. In this case, it is likely possible to extend one of the lines associated with a_i or a_{i+1} in order to lead to an equidistant intersection, but R2R does not implement this logic.

This functionality is implemented in `RnaDrawer.cpp`.

Bibliography

[1] R. Durbin, S. R. Eddy, A. Krogh, and G. Mitchison. *Biological Sequence Analysis: Probabilistic models of proteins and nucleic acids*. Cambridge, 1998.

[2] S. R. Eddy. *Infernal User's Guide*, 2003. ftp://ftp.genetics.wustl.edu/pub/eddy/software/infernal/Userguide.pdf.

[3] M. Gerstein, E. L. L. Sonnhammer, and C. Chothia. Volume changes in protein evolution. *Journal of Molecular Biology*, 236(4):1067–78, 1994.

[4] S. Griffiths-Jones. RALEE—RNA ALignment Editor in Emacs. *Bioinformatics*, 21:257–9, 2005.

[5] C. Lawrence, J. L. Zhou, and A. L. Tits. User's guide for CFSQP version 2.5: A C code for solving (large scale) constrained nonlinear (minimax) optimization problems, generating iterates satisfying all inequality constraints. Technical report, Institute for Systems Research, University of Maryland, College Park, 1997. TR-94-16r1.

[6] E. R. Lee, J. L. Baker, Z. Weinberg, N. Sudarsan, and R. R. Breaker. An allosteric self-splicing ribozyme triggered by a bacterial second messenger. *Science*, in press.

[7] J. Perreault, Z. Weinberg, A. Roth, O. Popescu, P. Chartrand, G. Ferbeyre, and R. R. Breaker. Identification of hammerhead ribozymes in all domains of life reveals novel structural variations. *PLoS Comput Biol*, 7:e1002031, 2011.

[8] E. Poiată, M. M. Meyer, T. D. Ames, and R. R. Breaker. A variant riboswitch aptamer class for *S*-adenosylmethionine common in marine bacteria. *RNA*, 15:2046–2056, 2009.

[9] E. E. Regulski, R. H. Moy, Z. Weinberg, J. E. Barrick, Z. Yao, W. L. Ruzzo, and R. R. Breaker. A widespread riboswitch candidate that controls bacterial genes involved in molybdenum cofactor and tungsten cofactor metabolism. *Mol Microbiol.*, 68(4):918–932, 2008.

[10] G. A. Soukup and R. R. Breaker. Relationship between internucleotide linkage geometry and the stability of RNA. *RNA*, 5:1308–25, 1999.

[11] N. Sudarsan, E. Lee, Z. Weinberg, R. Moy, J. Kim, K. Link, and R. Breaker. Riboswitches in eubacteria sense the second messenger cyclic di-GMP. *Science*, 321:411–413, 2008.

[12] J. X. Wang, E. R. Lee, D. R. Morales, J. Lim, and R. R. Breaker. Riboswitches that sense *S*-adenosylhomocysteine and activate genes involved in coenzyme recycling. *Mol Cell*, 29:691–702, 2008.

[13] Z. Weinberg, J. E. Barrick, Z. Yao, A. Roth, J. N. Kim, J. Gore, J. X. Wang, E. R. Lee, K. F. Block, N. Sudarsan, S. Neph, M. Tompa, W. L. Ruzzo, and R. R. Breaker. Identification of 22 candidate structured RNAs in bacteria using the CMfinder comparative genomics pipeline. *Nucleic Acids Res.*, 35(14):4809–4819, 2007.

[14] Z. Weinberg and R. R. Breaker. R2R—software to speed the depiction of aesthetic consensus RNA secondary structures. *BMC Bioinformatics*, 21:3, 2011.

[15] Z. Weinberg, J. Perreault, M. M. Meyer, and R. R. Breaker. Exceptional structured noncoding RNAs revealed by bacterial metagenome analysis. *Nature*, 462:656–659, 2009.

[16] Z. Weinberg, E. E. Regulski, M. C. Hammond, J. E. Barrick, Z. Yao, W. L. Ruzzo, and R. R. Breaker. The aptamer core of SAM-IV riboswitches mimics the ligand-binding site of SAM-I riboswitches. *RNA*, 14(5):822–828, 2008.

[17] Z. Weinberg, J. X. Wang, J. Bogue, J. Yang, K. Corbino, R. Moy, and R. R. Breaker. Comparative genomics reveals 104 candidate structured RNAs from bacteria, archaea and their metagenomes. *Genome Biol*, 11:R31, 2010.
